# Synthesis and Biological Evaluation of Novel 1,2,4-Triazole Derivatives Containing Amino Acid Fragments

**DOI:** 10.3390/molecules30081692

**Published:** 2025-04-10

**Authors:** Haoran Shi, Mingxu Li, Zhenghong Zhou, Aidang Lu, Ziwen Wang

**Affiliations:** 1 School of Chemical Engineering and Technology, Hebei University of Technology, Tianjin 300401, China; shr15130128422@163.com (H.S.); lmxlmx202412@163.com (M.L.); 2 State Key Laboratory of Elemento-Organic Chemistry, Research Institute of Elemento-Organic Chemistry, College of Chemistry, Nankai University, Tianjin 300071, China; z.h.zhou@nankai.edu.cn; 3 Tianjin Key Laboratory of Structure and Performance for Functional Molecules, College of Chemistry, Tianjin Normal University, Tianjin 300387, China

**Keywords:** plant diseases, novel fungicides, 1,2,4-triazole derivatives, amino acid fragments, antifungal activities, *Physalospora piricola*

## Abstract

Triazoles are important fragments in the development of fungicidal compounds. Fungi have gradually developed drug resistance against traditional fungicides due to long-term overuse. Therefore, there is an urgent need to discover new candidate compounds. A series of 1,2,4-triazole derivatives containing amino acid fragments were designed and synthesized based on mefentrifluconazole. All the target compounds were characterized by ^1^H-NMR, ^13^C-NMR, and HRMS techniques. Their antifungal activities against five kinds of phytopathogenic fungi were evaluated in vitro. The results revealed that most compounds had broad-spectrum fungicidal activities at 50 μg/mL and four compounds exhibited better antifungal activity than the control drug mefentrifluconazole. Interestingly, the synthesized compounds **8d** and **8k** exhibited exceptional antifungal activity against *Physalospora piricola*, with EC_50_ values of 10.808 µg/mL and 10.126 µg/mL, respectively. Molecular docking studies demonstrate that the 1,2,4-triazole derivatives **8d** and **8k**, which incorporate amino acid groups, exhibit strong binding affinity to 14α-demethylase (CYP51). These findings highlight the potential of these compounds as effective antifungal agents.

## 1. Introduction

The emergence of plant diseases caused by pathogenic fungi represents a significant threat to global food security [1]. Phytopathogenic fungi possess the remarkable ability to infect various plant tissues at all developmental stages, thereby disrupting crop growth and resulting in substantial reductions in yield and quality [2]. In addition, certain fungal infections are associated with the production of mycotoxins, which pose risks to mammalian health [3]. Despite these challenges, currently available antifungal products are limited by several critical shortcomings, including high toxicity, narrow spectrum of activity, safety concerns, and suboptimal pharmacokinetic properties [4,5]. As a result, there is an urgent need to develop novel, low-toxicity, and eco-friendly fungicides to support sustainable agricultural practices worldwide [6].

**Figure 1:**
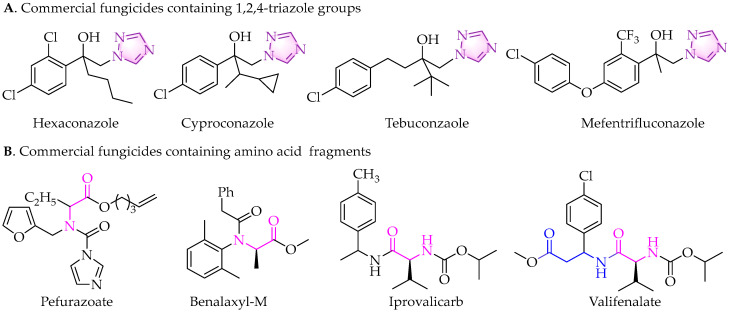
Chemical structures of some commercial fungicides containing triazole or amino acid fragments.

The triazole group is an important five-membered heterocyclic ring, which plays an important role in the construction of agricultural bioactive molecules. After decades of development, triazole derivatives have facilitated remarkable achievements in the discovery of pesticides, especially fungicides. Triazole units have two isomers: 1,2,3-triazole and 1,2,4-triazole [5,7]. Notably, 1,2,4-triazole serves as a key component in a variety of agricultural products, including fungicides, insecticides, and herbicides. Triazole fungicides mainly belong to sterol demethylation inhibitor (DMI) fungicides, a class of nitrogen-containing heterocyclic compounds that contain hydroxyl, substituted phenyl, and 1,2,4-triazole groups on the main chain of their chemical structures [5,7,8,9]. They inhibit the biosynthesis of ergosterol in fungi, causing abnormal membrane function and cell wall damage, thus achieving the effect of inhibiting and killing fungi [8]. Triadimefon is the first widely used triazole fungicide, characterized by its high efficiency, low toxicity, low residue, etc. Hexaconazole, cyproconazole, tebuconazole, mefentrifluconazole, and other similar compounds were subsequently developed and put into use (Figure 1A). [5,9] Mefentrifluconazole, developed by Badische Anilin-und-Soda-Fabrik (BASF), is the first isopropanol triazole broad-spectrum fungicide. It exhibited inhibitory activities against more than 60 difficult-to-control plant pathogens, such as *Alternaria alternata*, *Botrytis cinerea*, *Fusarium pseudograminearum*, *Monilinia fructicola*, *Colletotrichum scovillei*, etc. [10,11,12,13,14,15,16]. While the risk of plant pathogens developing resistance to mefentrifluconazole is relatively low-to-medium, long-term use of triazole fungicide still poses a risk of resistance development [17,18,19]. Consequently, it is imperative to continue developing novel pesticides to address this challenge.

The active substructure splicing strategy is one of the significant approaches in pesticide discovery research [20]. Based on this strategy in our previous work, a series of novel 1,2,4-triazole derivatives containing carboxamide fragments were designed and synthesized, and displayed outstanding anti-oomycete activity against Phytophthora capsici far superior to that of mefentrifluconazoles [21]. Amino acids not only serve as the fundamental building blocks of proteins but also are a type of important natural active substance. Naturally occurring amino acids exist widely in plants and microorganisms, and some of them exhibit significant pesticidal activities, including herbicidal, plant growth regulating, antibacterial, fungicidal, insecticidal, acaricidal, and nematocidal activities [22]. While some amino acids have been elaborated into agricultural chemicals, their structural fragments are also widely employed in the design of novel pharmaceuticals, such as pefurazoate, benalaxy-M, iprovalicard, valifenalate, etc. (Figure 1B) [22,23,24]. Introducing amino acid fragments into compounds can enhance their bioactivities, while simultaneously altering their physicochemical properties [25,26,27].

**Figure 2:**
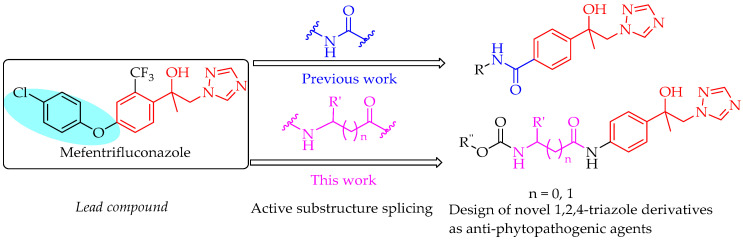
Design of 1,2,4-triazole derivatives containing amino acid fragments.

Encouraged by our previous work [21,27], a series of triazole compounds containing α/β amino acid fragments were designed based on the commercial fungicide mefentrifluconazole (Figure 2). Their antifungal activities against five kinds of phytopathogenic fungi (*Alternaria solani*, *Pyricularia oryzae*, *Sclerotinia sclerotiorum*, *Physalospora piricola*, and *Rhizoctonia cerealis*) were evaluated in vitro. Subsequently, the mechanisms of action of the two highly effective compounds **8d** and **8k** were preliminarily studied.

## 2. Results

### 2.1. Synthesis

**Figure 3:**
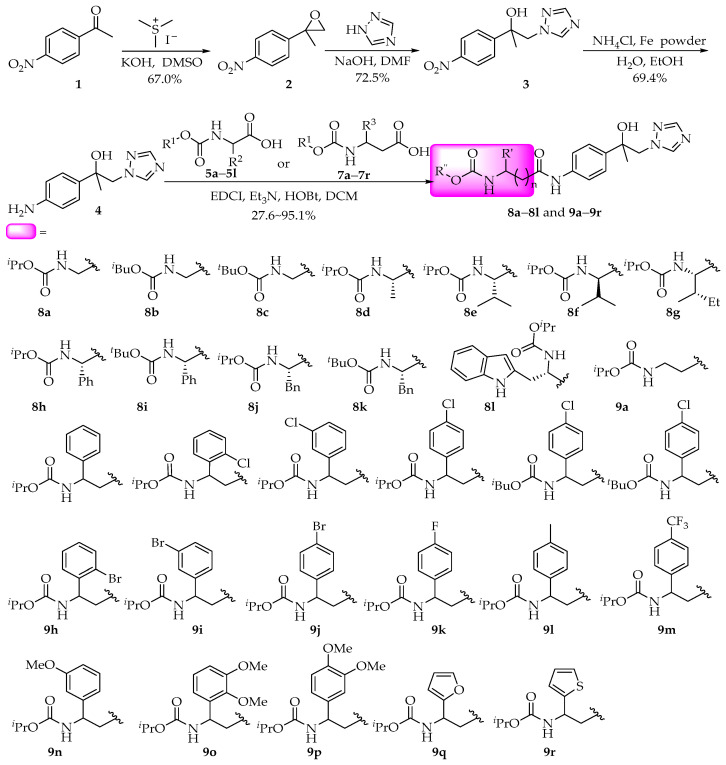
Synthesis of the compounds **8a**–**8l** and **9a**–**9r**.

Following the method described in Figure 3, 4-nitroacetophenone was used as the starting material. Through a four-step process involving carbonyl epoxidation, substitution, reduction, and amidation reaction, 30 1,2,4-triazole derivatives containing amino acid fragments were successfully synthesized. All the compounds were confirmed by ^1^H NMR, ^13^C NMR, and HRMS. Mefentrifluthconazole was synthesized according to the reported methods [21].

### 2.2. In Vitro Fungicidal Activities of Target Compounds ***8a***–***8l*** and ***9a***–***9r***

The target compounds **8a**–**8l** and **9a**–**9r** were assessed for their in vitro antifungal activity against five plant pathogenic fungi at 50 mg/L. As delineated in Table 1, four synthesized compounds (**8c**, **8d**, **8k**, **9e**) exhibited superior in vitro fungicidal efficacy against *Physalospora piricola* when benchmarked against the agricultural control agent mefentrifluconazole. At the standardized concentration of 50 mg/L, these derivatives demonstrated inhibition rates of >90% enhanced mycelial growth inhibition relative to the commercial mefentrifluconazole. Compound **8c** showed an 85% inhibition rate against *Rhizoctonia cerealis,* and compound **9e** exhibited an 85% inhibition rate against *Sclerotinia sclerotiorum*.

To understand the fungicidal activities of compounds **8d** and **8k** more clearly and intuitively, we determined the half maximal effective concentration (EC_50_) values for compounds **8d** and **8k,** and the results are shown in Table 2. Compounds **8d** and **8k** exhibited excellent in vitro activity against *Physalospora piricola*, with EC_50_ values of 10.808 and 10.126 μg/mL, superior to the intrinsic activity of mefentrifluconazole (EC_50_ = 14.433 μg/mL).

### 2.3. Molecular Docking Research

To investigate the molecular basis of antifungal activity, we performed rigid-receptor docking simulations using AutoDock Vina 1.1.2 on selected amino acid-functionalized 1,2,4-triazole derivatives against the lanosterol 14α-demethylase (CYP51) active site [28]. Comparative analysis revealed distinct binding modalities for compounds **8d** and **8k**, demonstrating Gibbs free energy values of −7.7 kcal/mol and −8.8 kcal/mol, respectively.

## 3. Discussion

### 3.1. Discussion of Synthesis

According to a similar approach reported, the 4-position acetyl of 4-nitroacetophenone was epoxidized with trimethylsulfonium iodide in the presence of NaH to obtain compound **2** starting from 4-nitroacetophenone (**1**), as shown in Figure 3. Compound **2** reacted with 1,2,4-triazole in DMF to obtain compound **3** through a substitution reaction [21,29]. Key intermediate **4** was obtained by the reduction of the nitro group from **3** in the presence of iron powder and NH_4_Cl. Compounds **5a**–**5l** were prepared by our reported methods [27]. Compounds **7a**–**7r** were prepared by the reported methods from aromatic aldehydes **6a**–**6r** with succinic acid and ammonium acetate [30]. The detailed synthetic procedure can be found in the Supplementary Materials. Compound **4** was amidated with α/β-amino acid derivatives **5a**–**5l** or **7a**–**7r** in the presence of 1-(3-dimethylaminopropyl)-3-ethylcarbodiimide hydrochloride (EDCI) and 1-hydroxybenzotriazole (HOBt) to obtain the target compounds **8a**–**8l** and **9a**–**9r**. The structures of obtained compounds were confirmed by ^1^H and ^13^C NMR spectroscopy and HRMS analysis.

### 3.2. Structure–Activity Relationship (SAR) for the Antifungal Activity In Vitro

The preliminary in vitro antifungal activities of target compounds **8a**–**8l** and **9a**–**9r** µg/mL are shown in Table 1, with commercial fungicide mefentrifluconazole as control. The results revealed that the target compounds showed broad antifungal activities against *Alternaria solani*, *Pyricularia oryzae*, *Sclerotinia sclerotiorum*, *Physalospora piricola*, and *Rhizoctonia cerealis* at the concentration of 50 µg/mL.

Various fungicides have amino acid structural units, such as pefurazoate, benalaxy-M, iprovalicard, valifenalate, etc. (Figure 1B). In our preliminary work, amino acid structural fragments introduced into flavone structural units significantly improved their antiviral activity [27]. Encouraged by these results, α-amino acid structural fragments were first introduced into the 4-position of the phenyl ring of mefentrifluconazole to replace the original ether group. To systematically investigate the effect of amino acid fragments with different structures on antifungal activity, 16 kinds of cheap and commercial amino acid derivatives were used as starting materials to react with compound **4** to obtain the target compounds **8a**–**8l**. In general, compared with compounds **8a**–**8l** containing amino acid fragments and mefentrifluconazole, some compounds showed higher inhibition rates against *Physalospora piricola*, whereas the inhibitory rates against *Alternaria solani*, *Pyricularia oryzae*, *Sclerotinia sclerotiorum*, and *Rhizoctonia cerealis* were not improved. Among compounds **8a**, **8d**–**8h**, **8j**, and **8l**, protected by the isopropoxycarbonyl group, *L*-alanine derivative **8d** had the best antifungal activity. *L*-glycine derivative (**8a**), *L*-valine derivative (**8e**), *L*-isoleucine derivative (**8g**), *L*-2-phenylglycine derivative (**8h**), *L*-phenylalanine derivative (**8j**), and *L*-tryptophan derivative (**8l**) were not conducive to antifungal activity (**8d** > **8a** > **8l** > **8j** > **8g** > **8h** > **8e**). In particular, the inhibition rate of compound **8e** was lower than 28%. The different protective groups of amino acids also had certain effects on antifungal activity. Compared with compounds **8a**, **8b**, and **8c**, when the amino protecting group was tert-butyloxycarbonyl, it was superior to isopropoxycarbonyl and iso-butyloxycarbonyl. This rule was consistent with the antifungal activity of compounds **8j** and **8k**. Notably, compound **8k** exhibited the highest inhibition rate (99%) against *Physalospora piricola* at 50 µg/mL. However, compared with compounds **8j** and **8k**, the antifungal activity of the amino protecting group with isopropoxycarbonyl was superior to tert-butyloxycarbonyl. By comparing the antifungal activity results of compounds **8e** and **8f**, it was found that the *D* configuration of the amino acid fragment was more favorable.

To further investigate the structure–activity relationship of these compounds, compounds **9a**–**9r** incorporating *β*-amino acid fragments were designed and synthesized. These amino acid fragments featured diverse substituents at the α position of the amino group, including a hydrogen atom and aromatic groups. As shown in Table 1, compounds **9a**–**9r** exhibited moderate to excellent antifungal activities against five plant pathogens: *Alternaria solani*, *Pyricularia oryzae*, *Sclerotinia sclerotiorum*, *Physalospora piricola*, and *Rhizoctonia cerealis*. Especially, compounds **9e**, **9f**, and **9o** demonstrated excellent antifungal activities of 85% or more against *Physalospora piricola*. The results revealed that the phenyl-substituted product (**9b**) exhibited significantly enhanced antifungal activity (56%) compared to the hydrogen-substituted compound (**9a**) (28%) against *Physalospora piricola*. Furthermore, introducing different substituents at the 4-position of the benzene ring group of *β*- amino acid fragments had a significant impact on antifungal activity against *Physalospora piricola*. The introduction of a chlorine atom (**9e**) at the 4-position significantly improved activity over that of **9b** (from 56% to 92%), whereas fluorine substitution (**9h**) slightly reduced activity, and bromine substitution (**9j**) had almost no significant effect. Interestingly, the incorporation of electron-donating groups, such as methyl (**9l**) and trifluoromethyl (**9m**), into the benzene ring of the amino acid fragments significantly reduced antifungal activity (inhibition rate: **9b** > **9m** > **9l**). As exhibited in Table 1, substituent position on the benzene ring also played a critical role in their antifungal activity. By comparing compounds **9c**–**9e** (inhibition rate: **9e** > **9d** > **9c**) and compounds **9h**–**9j** (inhibition rate: **9j** > **9i** > **9h**), the results demonstrated that the substitution at the 4-position of the benzene ring was most favorable for antifungal activity, whereas substitution at the 2-position of the benzene ring was disadvantageous. Substitution at the 3-position of the benzene ring had minimal impact. Additionally, compounds **9b**, **9d**, **9i**, and **9n** revealed that introducing different substituents at the 3-position of the benzene ring could only achieve a moderate inhibition rate (~50%). However, compounds **9n**, **9o**, and **9p** showed that the presence of two methoxy substituents at the 2,3 position of the benzene ring (**9o**) achieved an inhibition rate of 85% against *Physalospora piricola*. Among compounds containing different protecting groups (**9e**, **9f**, and **9g**) of amino groups, it was found that isopropoxycarbonyl protection had the highest antifungal activity (inhibition rate: **9e** > **9f** > **9g**). Conversely, compounds **9q** and **9r**, containing furyl and thienyl groups, exhibited relatively low to moderate antifungal activity.

To further evaluate the antifungal activity, the EC_50_ values of compounds **8d** and **8k** against *Physalospora piricola* were measured (Table 2). Compound **8d** exhibited remarkable antifungal activity with an EC_50_ value of 10.808 μg/mL against *Physalospora piricola*, and compound **8k** possessed an EC_50_ of 10.126 μg/mL. Both compounds outperformed mefentrifluconazole (EC_50_ = 14.433 μg/mL) under the same conditions. These results suggest that compounds incorporating amino acid fragments could potentially be promising candidates for controlling *Physalospora piricola*.

### 3.3. Molecular Docking

Given the structural homology of newly synthesized 1,2,4-triazole derivatives with mefentrifluconazole, a known CYP51 inhibitor that coordinates with the heme-iron cofactor in the enzyme’s active site, we conducted molecular docking studies to investigate their potential binding modes with 14α-demethylase (CYP51) [31]. Using the crystallographic coordinates of CYP51 (PDB: 3L4D), comparative docking simulations were performed for compounds **8d**, **8k**, and mefentrifluconazole [21].

**Figure 4:**
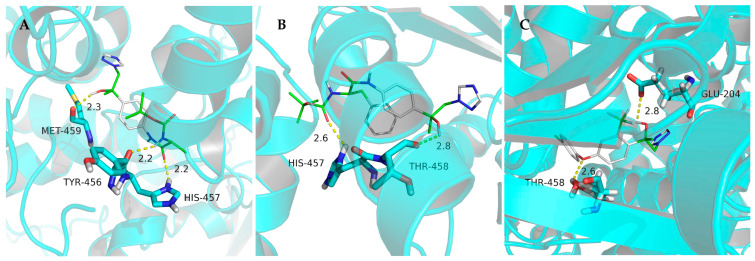
Molecular docking results for **8d** (**A**), **8k** (**B**), and mefentrifluconazole (**C**) with TMV CP.

Analysis of hydrogen bonding interactions revealed distinct binding patterns (Figure 4). For compound **8d**, the hydroxyl proton formed a 2.3 Å H-bond with the MET-459 hydroxyl group, while its amino acid substituent established dual interactions: the carbonyl oxygen engaged in a 2.2 Å hydrogen bond with HIS-457’s imidazole ring, and the amino group formed a 2.2 Å interaction with TYR-456 (Figure 4A). Compound **8k** exhibited bifurcated hydrogen bonding with catalytic residues THR-458 (2.8 Å) and HIS-457 (2.6 Å) (Figure 4B), contrasting with mefentrifluconazole’s binding geometry that involved GLU-204 (2.8 Å) and THR-458 (2.6 Å) (Figure 4C).

These calculation results are consistent with experimental antifungal activity data, indicating that the introduction of amino acid structural fragments can enhance the interaction force between drug molecules and CYP51. In addition, amino groups can also regulate the physical and chemical properties of compounds. This dual functional modification strategy explains the observed improvement in antifungal efficacy compared to the parent structure.

## 4. Materials and Methods

### 4.1. Chemicals

All chemical reagents were procured from Tianjin Guangda Chemical Reagents Ltd. (Tianjin, China) and used without further purification, with purity grades meeting or exceeding analytical reagent (AR) specifications. All anhydrous solvents were dried and purified by standard techniques before use. 1,2,4-Triazole derivatives containing amino acid fragments were prepared by the method as shown in Figure 3.

### 4.2. Instruments

Melting point determinations were performed using an X-4 binocular microscope system (Beijing Zhongke Instrument Co., Ltd., Beijing, China), with temperature calibration conducted before measurements. Structural characterization was accomplished through nuclear magnetic resonance spectroscopy using a Bruker 400 MHz (^1^H: 400 MHz; ^13^C: 100 MHz) spectrometer (Bruker, Billerica, MA, USA). Chemical shifts were internally referenced to residual solvent signals: CDCl_3_ (δ 7.26 ppm for ^1^H, *δ* 77.0 ppm for ^13^C) and DMSO-d_6_ (*δ* 2.5 ppm for ^1^H, *δ* 3.3 ppm for H_2_O, δ 39.9 ppm for ^13^C). Spectral multiplicity notations follow standard conventions: s (singlet), d (doublet), dd (double doublet), t (triplet), m (multiplet), and brs (broad singlet). High-resolution mass spectrometry (HRMS) data were acquired using a Varian QFT-ESI mass spectrometer equipped with Fourier transform capabilities (Varian, Palo Alto, CA, USA).

### 4.3. In Vitro Target Compounds Against Five Phytopathogenic Fungi

*Alternaria solani*, *Pyricularia oryzae*, *Sclerotinia sclerotiorum*, *Physalospora piricola*, and *Rhizoctonia cerealis* were selected to evaluate the antifungal activity of the target compounds, with the commercial fungicide mefentrifluconazole as the control. The in vitro antifungal activity test was performed using the mycelial growth rate method to evaluate the activity of the target compounds. Detailed procedures are provided in the Supplementary Materials.

### 4.4. Calculation Procedures for Molecular Docking Research

The 3D crystal structure of C-14α demethylase (PDB code: 3L4D) was downloaded from the protein data bank (PDB). Detailed procedures are provided in the Supplementary Materials.

### 4.5. Preparation of Compounds ***8***–***9***

A nitrogen-purged solution of KOH (1.529 g, 27.248 mmol) in anhydrous dimethyl sulfoxide (DMSO) (15 mL) was charged with trimethylsulfonium iodide (Me_3_S^+^I^−^) (7.414 g, 36.331 mmol) dissolved in anhydrous DMSO (75 mL) via cannula transfer. After 1 h of vigorous stirring at ambient temperature, compound **1** (3.000 g, 18.165 mmol) in anhydrous DMSO (30 mL) was added dropwise over 15 min. The resulting mixture was agitated for 2 h before being quenched with saturated NH_4_Cl solution (50 mL). Subsequent liquid–liquid extraction with EtOAc (3 × 100 mL), solvent evaporation under vacuum, and silica gel chromatography (hexane/EtOAc 3:1) afforded compound **2** as white crystals. The crude product was purified by column chromatography to obtain compound **2**. Light yellow solid, 67.0% yield, m.p. 33–34 °C; ^1^H NMR (400 MHz, DMSO-*d*_6_) *δ* 8.20 (d, *J* = 8.5 Hz, 2H), 7.62 (d, *J* = 8.4 Hz, 2H), 3.09 (d, *J* = 5.5 Hz, 1H), 2.82 (d, *J* = 5.1 Hz, 1H), 1.70 (s, 3H); ^13^C NMR (100 MHz, DMSO-*d*_6_) *δ* 149.3, 147.3, 127.2, 124.0, 57.2, 56.6, 21.3.

A suspension containing compound **2** (2.000 g, 11.162 mmol), 1,2,4-triazole (3.084 g, 44.648 mmol), and NaOH (0.893 g, 22.324 mmol) in anhydrous *N*,*N*-dimethylformamide (DMF) (40 mL) was refluxed at 110 °C for 4 h under N_2_. Following aqueous workup with saturated NH_4_Cl (30 mL), the product underwent sequential extraction (EtOAc 5 × 30 mL), brine wash, and desiccation over MgSO_4_. Recrystallization from the mixtures of CH_2_Cl_2_/diethyl ether yielded compound **3**. White solid, 72.5% yield, m.p. 148–150 °C; ^1^H NMR (400 MHz, DMSO-*d*_6_) *δ* 8.27 (s, 1H), 8.15 (d, *J* = 8.4 Hz, 2H), 7.81 (s, 1H), 7.70 (d, *J* = 8.5 Hz, 2H), 5.90 (s, 1H), 4.47 (s, 2H), 1.49 (s, 3H); ^13^C NMR (100 MHz, DMSO-*d*_6_) *δ* 154.0, 151.0, 146.9, 145.4, 127.3, 123.5, 73.3, 59.5, 27.6.

Compound **3** (3.000 g, 12.084 mmol), NH_4_Cl (0.646 g, 12.084 mmol), and H_2_O (10 mL) were added into anhydrous ethanol (60 mL). Iron powder (2.025 g, 36.252 mmol) was added, and the mixture stirred at 80 °C for 4 h. After cooling to room temperature, the solid was filtered, and the filtrate was extracted with ethyl acetate (3 × 15 mL). After evaporating the organic solvent, chromatographic purification gave compound **4** as a pale brown liquid with 69.4% yield. ^1^H NMR (400 MHz, DMSO-*d*_6_) *δ* 8.14 (s, 1H), 7.86 (s, 1H), 7.06 (d, *J* = 8.0 Hz, 2H), 6.49 (d, *J* = 7.9 Hz, 2H), 5.25 (s, 1H), 4.94 (s, 2H), 4.24 (s, 2H), 1.31 (s, 3H); ^13^C NMR (100 MHz, DMSO-*d*_6_) *δ* 150.7, 147.7, 145.2, 133.5, 126.2, 113.9, 72.7, 60.5, 27.4.

To a stirred solution of compound **4** (0.218 g, 1.0 mmol) in CH_2_Cl_2_ (5 mL) was sequentially added Et_3_N (0.202 g, 2.0 mmol), 1-hydroxybenzotriazole (HOBt) (0.203 g, 1.5 mmol), and 1-(3-dimethylaminopropyl)-3-ethylcarbodiimide hydrochloride (EDCI) (0.288 g, 1.5 mmol). After 30 min activation, amino acid derivatives **5a**–**5l** or **7a**–**7r** (1.1 mmol) were introduced portions. The reaction was monitored by TLC until completion (~12 h), then subjected to aqueous extraction (H_2_O/EtOAc 10 mL/15 mL ×3). The organic phase was dried over Na_2_SO_4_ and concentrated to afford crude product for subsequent characterization. After evaporating the solvent, the crude product was purified by column chromatography to obtain compounds **8a**–**8l** and **9a**–**9r**.

*Isopropyl (2-((4-(2-hydroxy-1-(1H-1,2,4-triazol-1-yl)propan-2-yl)phenyl)amino)-2-oxoethyl)carbamate* (**8a**). White solid, 50.0% yield, m.p. 176–178 °C; ^1^H NMR (400 MHz, DMSO-*d*_6_) *δ* 9.90 (s, 1H), 8.19 (s, 1H), 7.85 (s, 1H), 7.50 (d, *J* = 8.4 Hz, 2H), 7.34 (d, *J* = 8.3 Hz, 2H), 7.25 (t, *J* = 5.7 Hz, 1H), 5.49 (s, 1H), 4.79–4.72 (m, 1H), 4.37–4.29 (m, 2H), 3.75 (d, *J* = 6.1 Hz, 2H), 1.39 (s, 3H), 1.18 (d, *J* = 6.2 Hz, 6H); ^13^C NMR (100 MHz, DMSO-*d*_6_) *δ* 168.5, 156.9, 150.8, 145.3, 141.1, 138.0, 126.1, 119.0, 72.9, 67.6, 60.1, 44.4, 27.5, 22.5; HR-MS (ESI): calcd for C_17_H_23_N_5_O_4_ [M + H]^+^ 362.1823, found (ESI^+^) 362.1827.

*Isobutyl (2-((4-(2-hydroxy-1-(1H-1,2,4-triazol-1-yl)propan-2-yl)phenyl)amino)-2-oxoethyl)carbamate* (**8b**). White solid, 53.0% yield, m.p. 74–76 °C; ^1^H NMR (400 MHz, DMSO-*d*_6_) *δ* 9.93 (s, 1H), 8.20 (s, 1H), 7.85 (s, 1H), 7.50 (d, *J* = 8.5 Hz, 2H), 7.42–7.32 (m, 3H), 5.50 (s, 1H), 4.37–4.28 (m, 2H), 3.77–3.74 (m, 4H), 1.92–1.75 (m, 1H), 1.39 (s, 3H), 0.89 (d, *J* = 6.7 Hz, 6H); ^13^C NMR (100 MHz, DMSO-*d*_6_) *δ* 168.5, 157.4, 150.8, 145.3, 141.1, 138.0, 126.1, 119.0, 72.9, 70.5, 60.1, 44.4, 28.1, 27.5, 19.4; HR-MS (ESI): calcd for C_18_H_25_N_5_O_4_ [M + H]^+^ 376.1980, found (ESI^+^) 376.1986.

*Tert-butyl (2-((4-(2-hydroxy-1-(1H-1,2,4-triazol-1-yl)propan-2-yl)phenyl)amino)-2-oxoethyl)carbamate* (**8c**). White solid, 64.0% yield, m.p. 109–111 °C; ^1^H NMR (400 MHz, DMSO-*d*_6_) *δ* 9.89 (s, 1H), 8.20 (s, 1H), 7.85 (s, 1H), 7.50 (d, *J* = 8.6 Hz, 2H), 7.34 (d, *J* = 8.7 Hz, 2H), 7.08–7.02 (m, 1H), 5.49 (s, 1H), 4.37–4.28 (m, 2H), 3.70 (d, *J* = 6.1 Hz, 2H), 1.39 (s, 12H); ^13^C NMR (100 MHz, DMSO-*d*_6_) *δ* 168.6, 156.4, 150.9, 145.3, 141.1, 138.1, 126.1, 119.0, 78.6, 72.7, 60.1, 44.2, 28.7, 27.5; HR-MS (ESI): calcd for C_18_H_25_N_5_O_4_ [M + H]^+^ 376.1980, found (ESI^+^) 376.1983.

*Isopropyl ((2S)-1-((4-(2-hydroxy-1-(1H-1,2,4-triazol-1-yl)propan-2-yl)phenyl)amino)-1-oxopropan-2-yl)carbamate* (**8d**). White solid, 37.0% yield, m.p. 86–88 °C; ^1^H NMR (400 MHz, DMSO-*d*_6_) *δ* 9.92 (s, 1H), 8.20 (s, 1H), 7.85 (s, 1H), 7.51 (d, *J* = 7.8 Hz, 2H), 7.32 (m, 3H), 5.50 (s, 1H), 4.75–4.70 (m, 1H), 4.37–4.28 (m, 2H), 4.19–4.11 (m, 1H), 1.39 (s, 3H), 1.26 (d, *J* = 6.8 Hz, 3H), 1.16 (d, *J* = 6.3 Hz, 6H); ^13^C NMR (100 MHz, DMSO-*d*_6_) *δ* 172.2, 156.1, 150.9, 145.3, 141.1, 138.2, 126.0, 119.1, 72.9, 67.4, 60.1, 51.1, 27.6, 22.6, 18.6. HR-MS (ESI): calcd for C_18_H_25_N_5_O_4_ [M + H]^+^ 376.1980, found (ESI^+^) 376.1980.

*Isopropyl ((2S)-1-((4-(2-hydroxy-1-(1H-1,2,4-triazol-1-yl)propan-2-yl)phenyl)amino)-3-methyl-1-oxobutan-2-yl)carbamate* (**8e**). White solid, 66.0%yield, m.p. 91–93 °C; ^1^H NMR (400 MHz, DMSO-*d*_6_) *δ* 9.98 (s, 1H), 8.22 (s, 1H), 7.86 (s, 1H), 7.52 (d, *J* = 8.2 Hz, 2H), 7.35 (d, *J* = 8.3 Hz, 2H), 7.14 (d, *J* = 8.5 Hz, 1H), 5.49 (s, 1H), 4.78–4.71 (m, 1H), 4.37–4.29 (m, 2H), 3.94 (t, *J* = 8.1 Hz, 1H), 2.01–1.94 (m, 1H), 1.38 (s, 3H), 1.17 (t, *J* = 6.0 Hz, 6H), 0.89 (d, *J* = 6.8 Hz, 6H); ^13^C NMR (100 MHz, DMSO-*d*_6_) *δ* 171.1, 156.5, 150.9, 145.3, 141.3, 137.9, 126.1, 119.2, 72.9, 67.5, 61.4, 60.1, 30.8, 27.6, 22.6, 19.7, 19.0; HR-MS (ESI): calcd for C_20_H_29_N_5_O_4_ [M + H]^+^ 404.2293, found (ESI^+^) 404.2296.

*Isopropyl ((2R)-1-((4-(2-hydroxy-1-(1H-1,2,4-triazol-1-yl)propan-2-yl)phenyl)amino)-3-methyl-1-oxobutan-2-yl)carbamate* (**8f**). White solid, 64.0% yield, m.p. 90–92 °C; ^1^H NMR (400 MHz, DMSO-*d*_6_) *δ* 9.98 (s, 1H), 8.22 (s, 1H), 7.86 (s, 1H), 7.52 (d, *J* = 8.1 Hz, 2H), 7.35 (d, *J* = 8.3 Hz, 2H), 7.14 (d, *J* = 8.5 Hz, 1H), 5.49 (s, 1H), 4.38–4.29 (m, 1H), 4.75–4.70 (m, 1H), 3.94 (t, *J* = 8.0 Hz, 1H), 2.03–1.94 (m, 1H), 1.38 (s, 3H), 1.17 (t, *J* = 6.0 Hz, 6H), 0.89 (d, *J* = 6.9 Hz, 6H); ^13^C NMR (100 MHz, DMSO-*d*_6_) *δ* 171.1, 156.5, 150.9, 145.3, 141.3, 137.9, 126.1, 119.2, 72.9, 67.5, 61.4, 60.1, 30.8, 27.6, 22.6, 19.7, 19.0; HR-MS (ESI): calcd for C_20_H_29_N_5_O_4_ [M + H]^+^ 404.2293, found (ESI^+^) 404.2292.

*Isopropyl ((2S,3S)-1-((4-(2-hydroxy-1-(1H-1,2,4-triazol-1-yl)propan-2-yl)phenyl)amino)-3-methyl-1-oxopentan-2-yl)carbamate* (**8g**). White solid, 56.0% yield, m.p. 85–87 °C; ^1^H NMR (400 MHz, DMSO-*d*_6_) *δ* 9.99 (s, 1H), 8.22 (s, 1H), 7.86 (s, 1H), 7.52 (d, *J* = 8.2 Hz, 2H), 7.35 (d, *J* = 8.3 Hz, 2H), 7.15 (d, *J* = 7.8 Hz, 1H), 5.50 (s, 1H), 4.79–4.67 (m, 1H), 4.34–4.33 (m, 2H), 3.99–3.96 (m, 1H), 1.76 (s, 1H), 1.48 (s, 1H), 1.38 (s, 3H), 1.18–1.15 (m, 6H), 0.86–0.83 (m, 6H); ^13^C NMR (100 MHz, DMSO-*d*_6_) *δ* 171.3, 156.4, 150.8, 145.3, 141.3, 137.9, 126.1, 119.2, 72.9, 67.6, 60.2, 60.0, 36.8, 27.6, 25.1, 22.5, 15.8, 11.3; HR-MS (ESI): calcd for C_21_H_31_N_5_O_4_ [M + H]^+^ 418.2449, found (ESI^+^) 418.2446.

*Isopropyl ((1S)-2-((4-(2-hydroxy-1-(1H-1,2,4-triazol-1-yl)propan-2-yl)phenyl)amino)-2-oxo-1-phenylethyl)carbamate* (**8h**). White solid, 43.0% yield, m.p. 111–114 °C; ^1^H NMR (400 MHz, DMSO-*d*_6_) *δ* 10.25 (s, 1H), 8.20 (s, 1H), 7.84 (s, 1H), 7.78 (d, *J* = 8.3 Hz, 1H), 7.50 (d, *J* = 4.8 Hz, 2H), 7.37–7.27 (m, 5H), 5.50 (s, 1H), 5.40 (d, *J* = 8.4 Hz, 1H), 4.81–4.74 (m, 1H), 4.36–4.28 (m, 1H), 1.38 (s, 3H), 1.16–1.12 (m, 6H); ^13^C NMR (100 MHz, DMSO-*d*_6_) *δ* 169.4, 156.1, 150.8, 145.3, 141.5, 138.4, 137.8, 128.9, 128.4, 127.9, 126.1, 119.1, 72.9, 67.9, 60.1, 59.2, 27.6, 22.5; HR-MS (ESI): calcd for C_23_H_27_N_5_O_4_ [M + H]^+^ 438.2136, found (ESI^+^) 438.2134.

*Tert-butyl ((1S)-2-((4-(2-hydroxy-1-(1H-1,2,4-triazol-1-yl)propan-2-yl)phenyl)amino)-2-oxo-1-phenylethyl)carbamate* (**8i**). White solid, 80.0% yield, m.p. 110–113 °C; ^1^H NMR (400 MHz, DMSO-*d*_6_) *δ* 10.22 (s, 1H), 8.20 (s, 1H), 7.84 (s, 1H), 7.49 (d, *J* = 8.8 Hz, 5H), 7.37–7.27 (m, 5H), 5.49 (s, 1H), 5.34 (d, *J* = 8.3 Hz, 1H), 4.31 (d, *J* = 6.2 Hz, 2H), 1.39 (s, 9H), 1.37 (s, 3H); ^13^C NMR (100 MHz, DMSO-*d*_6_) *δ* 169.5, 155.6, 150.9, 145.3, 141.5, 138.6, 137.9, 128.9, 128.3, 127.9, 126.1, 119.1, 79.0, 72.9, 60.1, 59.0, 28.7, 27.6; HR-MS (ESI): calcd for C_24_H_29_N_5_O_4_ [M + H]^+^ 452.2293, found (ESI^+^) 452.2299.

*Isopropyl ((2S)-1-((4-(2-hydroxy-1-(1H-1,2,4-triazol-1-yl)propan-2-yl)phenyl)amino)-1-oxo-3-phenylpropan-2-yl)carbamate* (**8j**). White solid, 45.0% yield, m.p. 96–98 °C; ^1^H NMR (400 MHz, DMSO-*d*_6_) *δ* 10.06 (s, 1H), 8.21 (s, 1H), 7.86 (s, 1H), 7.51 (d, *J* = 8.1 Hz, 2H), 7.36–7.26 (m, 7H), 7.21–7.17 (m, 1H), 5.51 (s, 1H), 4.68–4.62 (m, 1H), 4.38–4.33 (m, 3H), 3.01–2.95 (m, 1H), 2.83 (t, *J* = 12.0 Hz, 1H), 1.39 (s, 3H), 1.13 (d, *J* = 6.2 Hz, 3H), 1.07 (d, *J* = 6.2 Hz, 3H); ^13^C NMR (100 MHz, DMSO-*d*_6_) *δ* 171.2, 156.3, 150.9, 145.3, 141.3, 138.5, 138.0, 129.8, 128.6, 126.8, 126.1, 119.2, 72.9, 67.5, 60.1, 57.3, 38.0, 27.6, 22.5; HR-MS (ESI): calcd for C_24_H_29_N_5_O_4_ [M + H]^+^ 452.2293, found (ESI^+^) 452.2294.

*Tert-butyl ((2S)-1-((4-(2-hydroxy-1-(1H-1,2,4-triazol-1-yl)propan-2-yl)phenyl)amino)-1-oxo-3-phenylpropan-2-yl)carbamate* (**8k**). White solid, 78.0% yield, m.p. 100–102 °C; ^1^H NMR (400 MHz, DMSO-*d*_6_) *δ* 10.02 (s, 1H), 8.22 (s, 1H), 7.86 (s, 1H), 7.51 (d, *J* = 8.7 Hz, 2H), 7.39–7.25 (m, 6H), 7.19 (t, *J* = 7.1 Hz, 1H), 7.11 (d, *J* = 8.3 Hz, 1H), 5.50 (s, 1H), 4.34–4.28 (m, 3H), 3.01–2.96 (m, 1H), 2.86–2.80 (m, 1H), 1.39 (s, 3H), 1.32 (s, 9H); ^13^C NMR (100 MHz, DMSO-*d*_6_) *δ* 171.2, 155.9, 150.9, 145.3, 141.3, 138.5, 138.0, 129.7, 128.6, 126.8, 126.1, 119.2, 78.6, 72.9, 60.1, 57.0, 38.0, 28.7, 27.6. HR-MS (ESI): calcd for C_25_H_31_N_5_O_4_ [M + H]^+^ 466.2449, found (ESI^+^) 466.2447.

*Isopropyl ((2S)-1-((4-(2-hydroxy-1-(1H-1,2,4-triazol-1-yl)propan-2-yl)phenyl)amino)-3-(1H-indol-3-yl)-1-oxopropan-2-yl)carbamate* (**8l**). White solid, 55.0% yield, m.p. 125–127 °C; ^1^H NMR (400 MHz, DMSO-*d*_6_) *δ* 10.82 (s, 1H), 10.09 (s, 1H), 8.22 (s, 1H), 7.86 (s, 1H), 7.68 (d, *J* = 7.8 Hz, 1H), 7.53 (d, *J* = 8.2 Hz, 2H), 7.39–7.30 (m, 3H), 7.26–7.16 (m, 2H), 7.06 (t, *J* = 7.6 Hz, 1H), 6.98 (t, *J* = 7.5 Hz, 1H), 5.51 (s, 1H), 4.69–4.65 (m, 1H), 4.42–4.33 (m, 3H), 3.12 (m, 1H), 3.05–2.93 (m, 1H), 1.40 (s, 3H), 1.15 (d, *J* = 6.2 Hz, 3H), 1.08 (d, *J* = 6.2 Hz, 3H); ^13^C NMR (100 MHz, DMSO-*d*_6_) *δ* 171.5, 156.2, 150.9, 145.3, 141.3, 138.1, 136.5, 127.8, 126.0, 124.4, 121.4, 119.4, 119.2, 118.7, 111.8, 110.4, 72.9, 67.5, 60.1, 56.5, 28.3, 27.6, 22.5; HR-MS (ESI): calcd for C_26_H_30_N_6_O_4_ [M + H]^+^ 491.2402, found (ESI^+^) 491.2401.

*Isopropyl (3-((4-(2-hydroxy-1-(1H-1,2,4-triazol-1-yl)propan-2-yl)phenyl)amino)-3-oxopropyl)carbamate* (**9a**). Yellow viscous solid, 27.6% yield, m.p. 79–81 °C; ^1^H NMR (400 MHz, DMSO-*d*_6_) *δ* 9.90 (s, 1H), 8.19 (s, 1H), 7.85 (s, 1H), 7.50 (d, *J* = 8.3 Hz, 2H), 7.32 (d, *J* = 8.1 Hz, 2H), 7.05 (s, 1H), 5.48 (s, 1H), 4.75–4.72 (m, 1H), 4.36–4.28 (m, 2H), 3.24 (m, 2H), 2.46 (t, *J* = 7.2 Hz, 2H), 1.38 (s, 3H), 1.15 (d, *J* = 6.2 Hz, 6H). ^13^C NMR (100 MHz, DMSO-*d*_6_) *δ* 169.9, 156.5, 150.8, 145.2, 140.9, 138.2, 126.0, 119.1, 79.7, 79.4, 79.0, 72.9, 60.1, 37.2, 27.5, 22.5; HR-MS (ESI): calcd for C_18_H_25_N_5_O_4_ [M + H]^+^ 376.1980, found (ESI^+^) 376.1986.

*Isopropyl (3-((4-(2-hydroxy-1-(1H-1,2,4-triazol-1-yl)propan-2-yl)phenyl)amino)-3-oxo-1-phenylpropyl)carbamate* (**9b**). White solid, 41.9% yield, m.p. 113–115 °C; ^1^H NMR (400 MHz, DMSO-*d*_6_) *δ* 9.84 (s, 1H), 8.19 (s, 1H), 7.85 (s, 1H), 7.72 (d, *J* = 7.7 Hz, 1H), 7.44 (d, *J* = 8.5 Hz, 2H), 7.31 (d, *J* = 8.6 Hz, 6H), 7.22 (t, *J* = 6.9 Hz, 1H), 5.47 (s, 1H), 5.05 (d, *J* = 8.2 Hz, 1H), 4.71–4.64 (m, 1H), 4.36–4.27 (m, 2H), 2.72 (d, *J* = 7.5 Hz, 2H), 1.37 (s, 3H), 1.15–1.10 (m, 6H); ^13^C NMR (100 MHz, DMSO-*d*_6_) *δ* 168.7, 155.6, 150.8, 145.2, 143.7, 141.1, 138.1, 128.7, 127.9, 127.4, 126.8, 126.0, 119.1, 110.1, 79.7, 79.4, 79.1, 72.9, 67.3, 60.1, 52.1, 44.0, 27.5, 22.5; HR-MS (ESI): calcd for C_24_H_29_N_5_O_4_ [M + H]^+^ 452.2293, found (ESI^+^) 452.2290.

*Isopropyl (1-(2-chlorophenyl)-3-((4-(2-hydroxy-1-(1H-1,2,4-triazol-1-yl)propan-2-yl)phenyl)amino)-3-oxopropyl)carbamate* (**9c**). White solid, 54.6% yield, m.p. 132–134 °C; ^1^H NMR (400 MHz, DMSO-*d*_6_) *δ* 9.84 (s, 1H), 8.19 (s, 1H), 7.85 (s, 1H), 7.77 (d, *J* = 8.0 Hz, 1H), 7.47 (d, *J* = 8.1 Hz, 3H), 7.40 (d, *J* = 7.8 Hz, 1H), 7.36–7.32 (m, 3H), 7.26 (t, *J* = 7.7 Hz, 1H), 5.48 (s, 1H), 5.42 (d, *J* = 6.7 Hz, 1H), 4.71–4.66 (m, 1H), 4.36–4.28 (m, 2H), 1.38 (s, 3H), 1.15 (d, *J* = 5.6 Hz, 6H), 1.12 (d, *J* = 6.4 Hz, 3H); ^13^C NMR (100 MHz, DMSO-*d*_6_) *δ* 168.2, 155.5, 150.8, 145.2, 141.1, 138.1, 132.0, 129.8, 129.2, 128.0, 127.9, 126.0, 119.1, 72.9, 67.6, 60.1, 49.1, 42.1, 27.6, 22.5; HR-MS (ESI): calcd for C_24_H_28_ClN_5_O_4_ [M + H]^+^ 486.1903, found (ESI^+^) 486.1901.

*Isopropyl (1-(3-chlorophenyl)-3-((4-(2-hydroxy-1-(1H-1,2,4-triazol-1-yl)propan-2-yl)phenyl)amino)-3-oxopropyl)carbamate* (**9d**). Yellow solid, 95.1% yield, m.p. 144–146 °C; ^1^H NMR (400 MHz, DMSO-*d*_6_) *δ* 9.87 (s, 1H), 8.19 (s, 1H), 7.85 (s, 1H), 7.82–7.71 (m, 1H), 7.41 (t, *J* = 11.5 Hz, 3H), 7.33–7.27(m, 5H), 5.47 (s, 1H), 5.04 (q, *J* = 8.0 Hz, 1H), 4.75–4.63 (m, 1H), 4.34–4.27 (m, 2H), 2.73 (d, *J* = 7.5 Hz, 2H), 1.37 (s, 3H), 1.16–1.11 (m, 6H); ^13^C NMR (100 MHz, DMSO-*d*_6_) *δ* 168.5, 155.7, 150.8, 146.1, 145.2, 141.1, 138.0, 133.4, 130.8, 127.6, 126.7, 126.0, 125.7, 125.1, 119.2, 110.2, 72.9, 67.6, 60.0, 51.8, 43.6, 27.5, 22.5; HR-MS (ESI): calcd for C_24_H_28_ClN_5_O_4_ [M + H]^+^ 486.1903, found (ESI^+^) 486.1901.

*Isopropyl (1-(4-chlorophenyl)-3-((4-(2-hydroxy-1-(1H-1,2,4-triazol-1-yl)propan-2-yl)phenyl)amino)-3-oxopropyl)carbamate* (**9e**). White solid, 80.9% yield, m.p. 178–181 °C; ^1^H NMR (400 MHz, DMSO-*d*_6_) *δ* 9.85 (s, 1H), 8.19 (s, 1H), 7.85 (s, 1H), 7.74 (d, *J* = 8.6 Hz, 1H), 7.39–7.31 (m, 6H), 5.45 (s, 1H), 5.03 (q, *J* = 7.8 Hz, 1H), 4.72–4.66 (m, 1H), 4.36–4.27 (m, 2H), 2.74–2.72 (m, 2H), 1.37 (s, 3H), 1.2 (d, *J* = 6.4 Hz, 6H); ^13^C NMR (100 MHz, DMSO-*d*_6_) *δ* 168.6, 155.7, 150.7, 145.2, 142.5, 141.1, 137.9, 132.0, 128.8, 128.7, 126.0, 72.9, 67.6, 60.0, 51.6, 43.6, 27.5, 22.5; HR-MS (ESI): calcd for C_24_H_28_ClN_5_O_4_ [M + H]^+^ 486.1903, found (ESI^+^) 486.1906.

*Isobutyl (1-(4-chlorophenyl)-3-((4-(2-hydroxy-1-(1H-1,2,4-triazol-1-yl)propan-2-yl)phenyl)amino)-3-oxopropyl)carbamate* (**9f**). White solid, 50.0% yield, m.p. 168–170 °C; ^1^H NMR (400 MHz, DMSO-*d*_6_) *δ* 9.87 (s, 1H), 8.19 (s, 1H), 7.85 (s, 2H), 7.44 (d, *J* = 8.4 Hz, 2H), 7.39–7.34 (m, 5H), 7.32 (d, *J* = 8.4 Hz, 2H), 5.48 (s, 1H), 5.03 (q, *J* = 7.6 Hz, 1H), 4.36–4.31(m, 2H), 3.73–3.63 (m, 2H), 2.73 (d, *J* = 7.7 Hz, 2H), 1.82–1.76 (m, 1H), 1.37 (s, 3H), 0.83 (d, *J* = 6.6 Hz, 6H); ^13^C NMR (100 MHz, DMSO-*d*_6_) *δ* 168.5, 156.2, 150.8, 145.2, 142.5, 141.1, 138.0, 132.0, 130.0, 128.8, 128.7, 126.0, 119.2, 72.9, 70.4, 60.0, 51.7, 43.6, 28.1, 27.5, 19.3; HR-MS (ESI): calcd for C_25_H_30_ClN_5_O_4_ [M + H]^+^ 500.2060, found (ESI^+^) 500.2061.

*Tert-butyl (1-(4-chlorophenyl)-3-((4-(2-hydroxy-1-(1H-1,2,4-triazol-1-yl)propan-2-yl)phenyl)amino)-3-oxopropyl)carbamate* (**9g**). White solid, 72.5% yield, m.p. 196–198 °C; ^1^H NMR (400 MHz, DMSO-*d*_6_) *δ* 9.85 (s, 1H), 8.19 (s, 1H), 7.85 (s, 1H), 7.56 (d, *J* = 8.1 Hz, 1H), 7.44 (d, *J* = 8.3 Hz, 2H), 7.36–7.31 (m, 6H), 5.46 (s, 1H), 5.00 (d, *J* = 8.1 Hz, 1H), 4.32 (dd, *J* = 20.4,14.0 Hz, 2H), 2.71 (d, *J* = 7.5 Hz, 2H), 1.37 (s, 3H), 1.34 (s, 9H); ^13^C NMR (100 MHz, DMSO-*d*_6_) *δ* 168.5, 155.2, 150.9, 145.3, 142.9, 141.1, 138.1, 131.9, 128.8, 128.7, 126.0, 119.1, 78.5, 72.9, 60.1, 51.3, 43.8, 28.7, 27.5; HR-MS (ESI): calcd for C_25_H_30_ClN_5_O_4_ [M + H]^+^ 500.2060, found (ESI^+^) 500.2063.

*Isopropyl (1-(2-bromophenyl)-3-((4-(2-hydroxy-1-(1H-1,2,4-triazol-1-yl)propan-2-yl)phenyl)amino)-3-oxopropyl)carbamate* (**9h**). Yellow solid, 34.3% yield, m.p. 128–130 °C; ^1^H NMR (400 MHz, DMSO-*d*_6_) *δ* 9.83 (s, 1H), 8.18 (s, 1H), 7.84 (s, 1H), 7.78 (d, *J* = 8.0 Hz, 1H), 7.57 (d, *J* = 8.0 Hz, 1H), 7.46 (t, *J* = 7.6 Hz, 4H), 7.33 (d, *J* = 8.3 Hz, 2H), 7.18 (t, *J* = 7.8 Hz, 1H), 5.49 (s, 1H), 5.36 (q, *J* = 8.0 Hz, 1H), 4.71–4.65 (m, 1H), 4.36–4.27 (m, 2H), 2.65–2.67(m, 2H), 1.38 (s, 3H), 1.15 (d, *J* = 5.6 Hz, 3H); 1.12 (d, *J* = 6.0 Hz, 3H); ^13^C NMR (100 MHz, DMSO-*d*_6_) *δ* 168.2, 155.5, 150.8, 145.2, 142.8, 141.1, 138.1, 133.1, 110.2, 129.4, 128.5, 128.0, 126.2, 126.0, 122.5, 119.1, 118.6, 113.9, 79.7, 79.4, 79.1, 72.9, 67.5, 60.1, 51.4, 42.1, 27.5, 22.5; HR-MS (ESI): calcd for C_24_H_28_BrN_5_O_4_ [M + H]^+^ 530.1398, found (ESI^+^) 530.1397.

*Isopropyl (1-(3-bromophenyl)-3-((4-(2-hydroxy-1-(1H-1,2,4-triazol-1-yl)propan-2-yl)phenyl)amino)-3-oxopropyl)carbamate* (**9i**). White solid, 54.9% yield, m.p. 97–99 °C; ^1^H NMR (400 MHz, DMSO-*d*_6_) *δ* 9.87 (s, 1H), 8.19 (s, 1H), 7.84 (s, 1H), 7.77 (d, *J* = 8.6 Hz, 1H), 7.54 (s, 1H), 7.43 (t, *J* = 8.5 Hz, 3H), 7.33–7.27 (m, 4H), 5.48 (s, 1H), 5.02 (q, *J* = 8.1 Hz, 1H), 4.71–4.67 (m, 1H), 4.36–4.27 (m, 2H), 2.73 (d, *J* = 7.3 Hz, 2H), 1.37 (s, 3H), 1.15 (d, *J* = 5.6 Hz, 3H); 1.12 (d, *J* = 6.4 Hz, 3H); ^13^C NMR (100 MHz, DMSO-*d*_6_) *δ* 168.4, 155.7, 150.8, 146.4, 145.2, 141.1, 138.0, 131.0, 130.3, 129.7, 126.0, 126.0, 122.1, 119.2, 118.6, 79.7, 79.4, 79.0, 72.9, 67.6, 60.1, 51.8, 43.7, 27.5, 22.5; HR-MS (ESI): calcd for C_24_H_28_BrN_5_O_4_ [M + H]^+^ 530.1398, found (ESI^+^) 530.1395.

*Isopropyl (1-(4-bromophenyl)-3-((4-(2-hydroxy-1-(1H-1,2,4-triazol-1-yl)propan-2-yl)phenyl)amino)-3-oxopropyl)carbamate* (**9j**). White solid, 29.8% yield, m.p. 195–198 °C; ^1^H NMR (400 MHz, DMSO-*d*_6_) *δ* 9.86 (s, 1H), 8.19 (s, 1H), 7.85 (s, 1H), 7.74 (dd, *J* = 23.2, 8.3 Hz, 1H), 7.51 (d, *J* = 8.0 Hz, 2H), 7.44 (d, *J* = 8.3 Hz, 2H), 7.30 (t, *J* = 9.7 Hz, 4H), 5.47 (s, 1H), 5.01 (d, *J* = 8.1 Hz, 1H), 4.70–4.67 (m, 1H), 4.36–4.27 (m, 2H), 2.72 (d, *J* = 7.6 Hz, 2H), 1.37 (s, 3H), 1.15 (d, *J* = 5.6 Hz, 3H); 1.12 (d, *J* = 6.4 Hz, 3H); ^13^C NMR (100 MHz, DMSO-*d*_6_) *δ* 168.5, 155.6, 150.8, 145.2, 143.1, 141.1, 138.0, 131.6, 129.2, 126.0, 120.5, 119.1, 72.9, 67.5, 60.1, 51.7, 43.6, 27.5, 22.5; HR-MS (ESI): calcd for C_24_H_28_BrN_5_O_4_ [M + H]^+^ 530.1398, found (ESI^+^) 530.1396.

*Isopropyl (1-(4-fluorophenyl)-3-((4-(2-hydroxy-1-(1H-1,2,4-triazol-1-yl)propan-2-yl)phenyl)amino)-3-oxopropyl)carbamate* (**9k**). White solid, 69.8% yield, m.p. 156–158 °C; ^1^H NMR (400 MHz, DMSO-*d*_6_) *δ* 9.85 (s, 1H), 8.19 (s, 1H), 7.85 (s, 1H), 7.74 (t, *J* = 8.6 Hz, 1H), 7.44 (d, *J* = 8.2 Hz, 2H), 7.38–7.30 (m, 4H), 7.13 (t, *J* = 8.4 Hz, 2H), 5.47 (s, 1H), 5.04 (q, *J* = 8.2 Hz, 1H), 4.71–4.67 (m, 1H), 4.36–4.27 (m, 2H), 2.72 (d, *J* = 7.6 Hz, 2H), 1.37 (s, 3H), 1.15 (d, *J* = 5.6 Hz, 3H); 1.12 (d, *J* = 6.4 Hz, 3H); ^13^C NMR (100 MHz, DMSO-*d*_6_) *δ* 168.6, 161.6 (d, *J* = 241.0 Hz), 155.6, 150.8, 145.2, 141.1, 139.8, 138.0, 128.8 (d, *J* = 8.0 Hz), 126.0, 119.2, 115.4 (d, *J* = 21.0 Hz), 72.9, 67.5, 60.1, 51.5, 43.9, 27.5, 22.5; HR-MS (ESI): calcd for C_24_H_29_FN_5_O_4_ [M + H]^+^ 470.2119, found (ESI^+^) 470.2117.

*Isopropyl (3-((4-(2-hydroxy-1-(1H-1,2,4-triazol-1-yl)propan-2-yl)phenyl)amino)-3-oxo-1-(p-tolyl)propyl)carbamate* (**9l**). White solid, 27.9% yield, m.p. 169–171 °C; ^1^H NMR (400 MHz, DMSO-*d*_6_) *δ* 9.83 (s, 1H), 8.19 (s, 1H), 7.85 (s, 1H), 7.65 (d, *J* = 8.3 Hz, 1H), 7.44 (d, *J* = 8.4 Hz, 2H), 7.31 (d, *J* = 8.3 Hz, 2H), 7.21 (d, *J* = 7.7 Hz, 2H), 7.10 (d, *J* = 7.7 Hz, 2H), 5.48 (s, 1H), 5.00 (q, *J* = 8.2 Hz, 1H), 4.68 (t, *J* = 6.8 Hz, 1H), 4.35–4.27 (m, 2H), 2.70 (d, *J* = 7.6 Hz, 2H), 2.25 (s, 3H), 1.37 (s, 3H), 1.15 (d, *J* = 5.6 Hz, 3H); 1.12 (d, *J* = 6.4 Hz, 3H); ^13^C NMR (100 MHz, DMSO-*d*_6_) *δ* 168.8, 155.7, 150.7, 145.2, 141.0, 140.5, 138.0, 136.6, 129.3, 126.8, 126.0, 119.2, 72.9, 67.4, 60.0, 51.9, 43.9, 27.6, 22.5, 21.1; HR-MS (ESI): calcd for C_25_H_31_N_5_O_4_ [M + H]^+^ 466.2449, found (ESI^+^) 466.2445.

*Isopropyl (3-((4-(2-hydroxy-1-(1H-1,2,4-triazol-1-yl)propan-2-yl)phenyl)amino)-3-oxo-1-(4-(trifluoromethyl)phenyl)propyl)carbamate* (**9m**). White solid, 21.2% yield, m.p. 200–202 °C; ^1^H NMR (400 MHz, DMSO-*d*_6_) *δ* 9.89 (s, 1H), 8.19 (s, 1H), 7.86 (d, *J* = 9.4 Hz, 2H), 7.70 (d, *J* = 7.9 Hz, 2H), 7.55 (d, *J* = 7.9 Hz, 2H), 7.45 (d, *J* = 8.4 Hz, 2H), 7.32 (d, *J* = 8.3 Hz, 2H), 5.47 (s, 1H), 5.15–5.11 (m, 1H), 4.71–4.67 (m, 1H), 4.36–4.27 (m, 2H), 2.77 (d, *J* = 7.5 Hz, 2H), 1.37 (s, 3H), 1.15 (d, *J* = 5.6 Hz, 3H); 1.12 (d, *J* = 6.4 Hz, 3H); ^13^C NMR (100 MHz, DMSO-*d*_6_) *δ* 168.4, 155.7, 150.8, 148.4, 145.2, 141.2, 138.0, 127.7, 126.0, 125.8, 125.7, 123.6, 119.2, 72.9, 67.6, 60.1, 51.9, 43.5, 27.5, 22.5; HR-MS (ESI): calcd for C_25_H_28_F_3_N_5_O_4_ [M + H]^+^ 520.2167, found (ESI^+^) 520.2173.

*Isopropyl (3-((4-(2-hydroxy-1-(1H-1,2,4-triazol-1-yl)propan-2-yl)phenyl)amino)-1-(3-methoxyphenyl)-3-oxopropyl)carbamate* (**9n**). Yellow solid, 57.6% yield, m.p. 105–108 °C; ^1^H NMR (400 MHz, DMSO-*d*_6_) *δ* 9.84 (s, 1H), 8.19 (s, 1H), 7.85 (s, 1H), 7.71 (d, *J* = 8.7 Hz, 1H), 7.45 (d, *J* = 8.4 Hz, 2H), 7.32 (d, *J* = 8.3 Hz, 2H), 7.21 (t, *J* = 7.8 Hz, 1H), 6.90 (d, *J* = 7.6 Hz, 2H), 6.78 (d, *J* = 7.9 Hz, 1H), 5.48 (s, 1H), 5.03 (m, 1H), 4.69 (t, *J* = 6.4 Hz, 1H), 4.364.28– (m, 2H), 3.72 (s, 3H), 2.71 (d, *J* = 7.4 Hz, 2H), 1.37 (s, 3H), 1.15 (d, *J* = 5.6 Hz, 3H); 1.12 (d, *J* = 6.4 Hz, 3H); ^13^C NMR (100 MHz, DMSO-*d*_6_) *δ* 168.7, 159.7, 155.7, 150.8, 145.3, 145.2, 141.1, 138.1, 129.8, 128.3, 127.8, 126.0, 125.1, 119.6, 119.2, 119.1, 118.6, 112.7, 112.6, 110.1, 79.7, 79.4, 79.1, 72.9, 67.4, 60.1, 55.4, 52.1, 44.0, 27.5, 22.5; HR-MS (ESI): calcd for C_25_H_31_N_5_O_5_ [M + H]^+^ 482.2398, found (ESI^+^) 482.2404.

*Isopropyl (1-(2,3-dimethoxyphenyl)-3-((4-(2-hydroxy-1-(1H-1,2,4-triazol-1-yl)propan-2-yl)phenyl)amino)-3-oxopropyl)carbamate* (**9o**). Yellow solid, 31.3% yield, m.p. 164–166 °C; ^1^H NMR (400 MHz, DMSO-*d*_6_) *δ* 9.77 (s, 1H), 8.18 (s, 1H), 7.84 (s, 1H), 7.71 (d, *J* = 8.4 Hz, 1H), 7.47 (d, *J* = 8.1 Hz, 2H), 7.32 (d, *J* = 8.2 Hz, 2H), 7.02 (t, *J* = 8.0 Hz, 1H), 6.91 (t, *J* = 6.3 Hz, 2H), 5.47 (s, 1H), 5.40–5.34 (m, 1H), 4.70–4.65 (m, 1H), 4.36–4.28 (m, 2H), 3.78 (s, 6H), 2.77–2.52 (m, 2H), 1.38 (s, 3H), 1.13 (d, *J* = 5.6 Hz, 3H); 1.10 (d, *J* = 6.4 Hz, 3H); ^13^C NMR (100 MHz, DMSO-*d*_6_) *δ* 169.3, 155.5, 152.7, 150.8, 146.7, 145.2, 141.0, 138.2, 137.3, 128.3, 127.9, 126.2, 126.0, 125.1, 124.4, 119.6, 119.2, 119.1, 119.0, 118.2, 111.9, 110.1, 79.7, 79.4, 79.1, 72.9, 67.3, 60.6, 60.1, 56.1, 46.5, 43.2, 27.5, 22.6; HR-MS (ESI): calcd for C_26_H_35_N_5_O_6_ [M + H]^+^ 512.2504, found (ESI^+^) 512.2498.

*Isopropyl (1-(3,4-dimethoxyphenyl)-3-((4-(2-hydroxy-1-(1H-1,2,4-triazol-1-yl)propan-2-yl)phenyl)amino)-3-oxopropyl)carbamate* (**9p**). White solid, 43.9% yield, m.p. 95–98 °C; ^1^H NMR (400 MHz, DMSO-*d*_6_) *δ* 9.83 (s, 1H), 8.19 (s, 1H), 7.85 (s, 1H), 7.61 (d, *J* = 8.8 Hz, 1H), 7.45 (d, *J* = 8.4 Hz, 2H), 7.31 (d, *J* = 8.4 Hz, 2H), 6.95 (s, 1H), 6.87–6.81 (m, 2H), 5.46 (s, 1H), 5.03–4.97 (m, 1H), 4.73–4.66(m, 1H), 4.36–4.27 (m, 2H), 3.72 (s, 3H), 3.70 (s, 3H), 2.70 (d, *J* = 7.1 Hz, 2H), 1.37 (s, 3H), 1.16 (d, *J* = 5.6 Hz, 3H), 1.12 (d, *J* = 6.0 Hz, 3H); ^13^C NMR (100 MHz, DMSO-*d*_6_) *δ* 168.8, 155.6, 150.8, 149.0, 148.2, 145.3, 141.1, 138.1, 136.0, 126.0, 119.1, 118.9, 112.0, 110.7, 72.9, 67.4, 60.1, 56.0, 55.9, 51.9, 44.2, 27.5, 22.5; HR-MS (ESI): calcd for C_26_H_35_N_5_O_6_ [M + H]^+^ 512.2504, found (ESI^+^) 512.2497.

*Isopropyl (1-(furan-2-yl)-3-((4-(2-hydroxy-1-(1H-1,2,4-triazol-1-yl)propan-2-yl)phenyl)amino)-3-oxopropyl)carbamate* (**9q**). Yellow solid, 69.0% yield, m.p. 134–136 °C; ^1^H NMR (400 MHz, DMSO-*d*_6_) *δ* 9.95 (s, 1H), 8.20 (s, 1H), 7.85 (s, 1H), 7.57 (d, *J* = 12.9 Hz, 2H), 7.48 (d, *J* = 8.3 Hz, 2H), 7.33 (d, *J* = 8.3 Hz, 2H), 6.36 (s, 1H), 6.21 (s, 1H), 5.47 (s, 1H), 5.17–5.11 (m, 1H), 4.77–4.72 (m, 1H), 4.36–4.28 (m, 2H), 2.90–2.69 (m, 2H), 1.38 (s, 3H), 1.15 (d, *J* = 6.3 Hz, 6H); ^13^C NMR (101 MHz, DMSO-*d*_6_) *δ* 168.4, 155.7, 155.5, 150.8, 145.2, 142.4, 141.1, 138.1, 126.0, 119.1, 110.8, 106.1, 79.7, 79.4, 79.1, 72.9, 67.5, 60.1, 46.1, 40.9, 27.5, 22.5; HR-MS (ESI): calcd for C_22_H_27_N_5_O_5_ [M + H]^+^ 442.2085, found (ESI^+^) 442.2090.

*Isopropyl (3-((4-(2-hydroxy-1-(1H-1,2,4-triazol-1-yl)propan-2-yl)phenyl)amino)-3-oxo-1-(thiophen-2-yl)propyl)carbamate* (**9r**). Yellow solid, 51.9% yield, m.p. 86–88 °C; ^1^H NMR (400 MHz, DMSO-*d*_6_) *δ* 9.97 (s, 1H), 8.18 (s, 1H), 7.83 (s, 1H), 7.70 (d, *J* = 7.6 Hz, 1H), 7.44 (d, *J* = 8.0 Hz, 2H), 7.31–7.29 (m, 3H), 6.95–6.93 (m, 2H), 5.59 (s, 1H), 5.32–5.25 (m, 1H), 4.74–4.68 (m, 1H), 4.32 (s, 2H), 2.89–2.77 (m, 2H), 1.37 (s, 3H), 1.12 (d, *J* = 6.0 Hz, 6H); ^13^C NMR (100 MHz, DMSO-*d*_6_) *δ* 168.6, 155.8, 150.7, 147.2, 145.2, 141.1, 138.0, 127.3, 126.0, 124.9, 124.2, 119.3, 79.6, 79.2, 78.9, 72.9, 67.8, 60.0, 47.8, 43.6, 27.5, 22.5, 22.4; HR-MS (ESI): calcd for C_22_H_27_N_5_O_4_S [M + H]^+^ 458.1857, found (ESI^+^) 458.1851.

## 5. Conclusions

In conclusion, a series of novel 1,2,4-triazole derivatives incorporating α/β-amino acid fragments were designed and synthesized with the aim of investigating their antifungal activities. Their fungicidal activities against five phytopathogenic fungi at a concentration of 50 µg/mL were systematically evaluated. The bioassay results indicated that most of the synthesized compounds exhibited antifungal activity against *Physalospora piricola*, with compounds **8d** and **8k** demonstrating superior antifungal activity compared to the commercial DMI fungicide mefentrifluconazole. Among the compounds **8a**–**8l** and **9a**–**9r**, compounds **8d** (EC_50_ = 10.808 µg/mL) and **8k** (EC_50_ = 10.126 µg/mL) exhibited significantly higher potency than mefentrifluconazole (EC_50_ = 14.433 µg/mL). Molecular docking analysis further confirmed that compounds **8d** and **8k** exhibited stronger binding affinities toward CYP51. According to the molecular docking results, it is preliminarily indicated that compounds **8d** and **8k** have excellent antifungal activity that is related to their strong binding with CYP51. These novel 1,2,4-triazole derivatives incorporating α/β-amino acid fragments could serve as promising candidates for the development of more potent fungicides to combat pathogenic fungi.

## Figures and Tables

**Table 1:** In vitro fungicidal activities of compounds **8a**–**8l**, **9a**–**9r**, and mefentrifluconazole at 50 µg/mL ^1^.

Comp.	Inhibition Rate (%)					Comp.	Inhibition Rate (%)				
	*A.s.* ^2^	*P.o.* ^2^	*S.s.* ^2^	*P.p.* ^2^	*R.c.* ^2^		*A.s.* ^2^	*P.o.* ^2^	*S.s.* ^2^	*P.p.* ^2^	*R.c.* ^2^
**8a**	17 ± 2	13 ± 1	46 ± 3	78 ± 3	67 ± 2	**9e**	19 ± 3	20 ± 3	85 ± 2	92 ± 3	62 ± 2
**8b**	29 ± 2	33 ± 1	21 ± 1	28 ± 2	31 ± 1	**9f**	14 ± 1	20 ± 3	33 ± 1	85 ± 3	62 ± 2
**8c**	24 ± 1	33 ± 1	38 ± 2	92 ± 3	80 ± 2	**9g**	29 ± 2	33 ± 1	46 ± 1	56 ± 1	46 ± 2
**8d**	24 ± 1	33 ± 1	38 ± 2	94 ± 1	37 ± 3	**9h**	29 ± 2	33 ± 1	44 ± 2	41 ± 1	22 ± 2
**8e**	19 ± 3	20 ± 3	25 ± 2	28 ± 2	19 ± 3	**9i**	33 ± 1	27 ± 2	25 ± 2	51 ± 2	35 ± 2
**8f**	19 ± 3	27 ± 2	31 ± 2	70 ± 1	62 ± 2	**9j**	24 ± 1	40 ± 3	48 ± 3	70 ± 1	25 ± 2
**8g**	17 ± 2	7 ± 2	6 ± 2	53 ± 3	31 ± 3	**9k**	24 ± 1	33 ± 1	33 ± 1	37 ± 2	19 ± 3
**8h**	24 ± 1	27 ± 2	31 ± 2	49 ± 1	43 ± 2	**9l**	10 ± 3	27 ± 2	33 ± 1	32 ± 1	22 ± 2
**8i**	19 ± 3	33 ± 1	53 ± 3	28 ± 2	31 ± 1	**9m**	10 ± 3	33 ± 1	33 ± 1	38 ± 3	25 ± 2
**8j**	14 ± 1	33 ± 1	61 ± 2	56 ± 1	43 ± 2	**9n**	19 ± 3	40 ± 3	61 ± 2	47 ± 3	31 ± 1
**8k**	14 ± 1	33 ± 1	74 ± 1	99 ± 2	74 ± 2	**9o**	24 ± 1	27 ± 2	25 ± 2	85 ± 3	62 ± 2
**8l**	14 ± 1	7 ± 2	48 ± 3	63 ± 1	49 ± 1	**9p**	24 ± 1	47 ± 2	69 ± 1	51 ± 2	53 ± 2
**9a**	24 ± 1	47 ± 2	71 ± 3	28 ± 2	31 ± 1	**9q**	14 ± 1	40 ± 3	33 ± 1	18 ± 1	12 ± 1
**9b**	24 ± 1	33 ± 1	36 ± 2	56 ± 1	37 ± 3	**9r**	19 ± 3	33 ± 1	25 ± 2	49 ± 1	49 ± 1
**9c**	43 ± 1	47 ± 2	33 ± 1	52 ± 2	22 ± 2	mefentrifluconazole	67 ± 2	100	100	85 ± 3	99 ± 1
**9d**	19 ± 3	33 ± 1	41 ± 3	58 ± 2	46 ± 2						

^1^ The experiments were repeated three times. All results are expressed as mean ± SD. ^2^ *A.s.* = *Alternaria solani*; *P.o.* = *Pyricularia oryzae*; *S.s.* = *Sclerotinia sclerotiorum*; *P.p.* = *Physalospora piricola*; *R.c.* = *Rhizoctonia cerealis*.

**Table 2:** In vitro EC_50_ values (µg/mL) of selected compounds against *Physalospora piricola* ^1^.

Strain	Compound	EC_50_ (μg/mL)	95% Confidence Interval	Regression Equation	*R* ^2^
*P. piricola*	**8d**	10.808	9.244–12.583	y = 3.203x − 3.311	0.989
	**8k**	10.126	7.205–13.911	y = 3.916x − 3.938	0.966
	mefentrifluconazole	14.433	12.179–17.207	y = 2.759x − 3.198	0.974

^1^ The experiments were repeated 3 times.

## Data Availability

All data used to support the findings of this study are included within the article and Supplementary Materials.

## Supplementary Materials

The following supporting information can be downloaded at https://www.mdpi.com/article/10.3390/molecules30081692/s1. Section S1. General synthetic procedures for target compound amino acid derivatives (Scheme S1: Synthesis of compounds **7a**–**7r**); Section S2: Detailed bioassay procedures for the in vitro antifungal activities; Section S3: Calculation procedures for molecular docking research; Section S4: Copies of NMR spectra (Figures S1–S66). References [32,33] are cited in Supporting Information.
