# Supplementary material for: Synthesis and Biological Evaluation of Novel 1,2,4-Triazole Derivatives Containing Amino Acid Fragments
Source: Molecules. 2025 Apr 10;30(8):1692. doi: 10.3390/molecules30081692 (PMC12029508; doi:10.3390/molecules30081692)
Supplement: Supplementary file 1 [file molecules-30-01692-s001.zip › Supporting Information-R1.pdf]

Supplementary Material

Synthesis and Biological Evaluation of Novel 1,2,4-Triazole Derivatives Containing Amino Acid Fragments

Haoran Shi [1,†], Mingxu Li [1,†], Zhenghong Zhou[3], Aidang Lu[1,*] and Ziwen Wang[2,*]
1. School of Chemical Engineering and Technology, Hebei University of Technology, Tianjin 300401, China; shr15130128422@163.com (H. S.); lmxlmx202412@163.com (M. L.); luaidang@hebut.edu.cn (A. L.)
2. Tianjin Key Laboratory of Structure and Performance for Functional Molecules, College of Chemistry,
Tianjin Normal University, Tianjin 300387, China; hxxywzw@tjnu.edu.cn (Z.W.)
3. State Key Laboratory of Elemento-Organic Chemistry, Research Institute of Elemento-Organic Chemistry, College of Chemistry, Nankai University, Tianjin 300071, China; z.h.zhou@nankai.edu.cn (Z. Z.)
*Correspondence: hxxywzw@tjnu.edu.cn (Z.W.); luaidang@hebut.edu.cn (A.L.); Tel.: +86-22-23766531 (Z.W.); +86-22-60202812 (A.L.)
† These authors contributed equally to this work.

Contents

Section S1. General synthetic procedures for target compounds amino acid derivatives 7a–7r 4

Section S2. Detailed bioassay procedures for the in vitro antifungal activities 6

Section S3. Calculation procedures for molecular docking research 6

Section S4. Figures S1–S66. ^{1}H, and ^{13}C NMR spectra of 2–4, 8–9 8

Figure S1. ^{1}H NMR spectrum of 2 8

Figure S2. ^{13}C NMR spectrum of 2 8

Figure S3. ^{1}H NMR spectrum of 3 9

Figure S4. ^{13}C NMR spectrum of 3 9

Figure S5. ^{1}H NMR spectrum of 4 10

Figure S6. ^{13}C NMR spectrum of 4 10

Figure S7. ^{1}H NMR spectrum of 8a 11

Figure S8. ^{13}C NMR spectrum of 8a 11

Figure S9. ^{1}H NMR spectrum of 8b 12
Figure S10. ^{13}C NMR spectrum of 8b 12
Figure S11. ^{1}H NMR spectrum of 8c 13
Figure S12. ^{13}C NMR spectrum of 8c 13
Figure S13. ^{1}H NMR spectrum of 8d 14
Figure S14. ^{13}C NMR spectrum of 8d 14
Figure S15. ^{1}H NMR spectrum of 8e 15
Figure S16. ^{13}C NMR spectrum of 8e 15
Figure S17. ^{1}H NMR spectrum of 8f 16
Figure S18. ^{13}C NMR spectrum of 8f 16
Figure S19. ^{1}H NMR spectrum of 8g 17
Figure S20. ^{13}C NMR spectrum of 8g 17
Figure S21. ^{1}H NMR spectrum of 8h 18
Figure S22. ^{13}C NMR spectrum of 8h 18
Figure S23. ^{1}H NMR spectrum of 8i 19
Figure S24. ^{13}C NMR spectrum of 8i 19
Figure S25. ^{1}H NMR spectrum of 8j 20
Figure S26. ^{13}C NMR spectrum of 8j 20
Figure S27. ^{1}H NMR spectrum of 8k 21
Figure S28. ^{13}C NMR spectrum of 8k 21
Figure S29. ^{1}H NMR spectrum of 8l 22
Figure S30. ^{13}C NMR spectrum of 8l 22
Figure S31. ^{1}H NMR spectrum of 9a 23
Figure S32. ^{13}C NMR spectrum of 9a 23
Figure S33. ^{1}H NMR spectrum of 9b 24
Figure S34. ^{13}C NMR spectrum of 9b 24
Figure S35. ^{1}H NMR spectrum of 9c 25
Figure S36. ^{13}C NMR spectrum of 9c 25
Figure S37. ^{1}H NMR spectrum of 9d 26

Figure S38. ^{13}C NMR spectrum of 9d 26
Figure S39. ^{1}H NMR spectrum of 9e 27
Figure S40. ^{13}C NMR spectrum of 9e 27
Figure S41. ^{1}H NMR spectrum of 9f 28
Figure S42. ^{13}C NMR spectrum of 9f 28
Figure S43. ^{1}H NMR spectrum of 9g 29
Figure S44. ^{13}C NMR spectrum of 9g 29
Figure S45. ^{1}H NMR spectrum of 9h 30
Figure S46. ^{13}C NMR spectrum of 9h 30
Figure S47. ^{1}H NMR spectrum of 9i 31
Figure S48. ^{13}C NMR spectrum of 9i 31
Figure S49. ^{1}H NMR spectrum of 9j 32
Figure S50. ^{13}C NMR spectrum of 9j 32
Figure S51. ^{1}H NMR spectrum of 9k 33
Figure S52. ^{13}C NMR spectrum of 9k 33
Figure S53. ^{1}H NMR spectrum of 9l 34
Figure S54. ^{13}C NMR spectrum of 9l 34
Figure S55. ^{1}H NMR spectrum of 9m 35
Figure S56. ^{13}C NMR spectrum of 9m 35
Figure S57. ^{1}H NMR spectrum of 9n 36
Figure S58. ^{13}C NMR spectrum of 9n 36
Figure S59. ^{1}H NMR spectrum of 9o 37
Figure S60. ^{13}C NMR spectrum of 9o 37
Figure S61. ^{1}H NMR spectrum of 9p 38
Figure S62. ^{13}C NMR spectrum of 9p 38
Figure S63. ^{1}H NMR spectrum of 9q 39
Figure S64. ^{13}C NMR spectrum of 9q 39
Figure S65. ^{1}H NMR spectrum of 9r 40
Figure S66. ^{13}C NMR spectrum of 9r 40

Section S1. General synthetic procedures for target compounds amino acid derivatives 7a–7r

Regarding the acquisition of α-amino acid derivatives: Obtained through commercial purchase.

General Synthetic Procedures for Target Compounds **6**. Various substituted aldehyde compounds **5** (40 mmol), malonic acid (8.325 g, 80 mmol), and ammonium acetate (6.166 g, 80 mmol) were added to anhydrous ethanol (60 mL) under nitrogen and stirred at 80 °C for 24 h. After cooling to room temperature, the filter cake was collected and dried to obtain compound **6a–6r**.

General Synthetic Procedures for Target Compounds **7a–7r**. Compound **6** (10 mmol) and various substituted chloroformate (12 mmol) were added to sodium hydroxide solution (2 N, 10 mL) at 0 °C. The reaction mixture was left stirring for 1 h at room temperature. After dissolving in EtOAc (4 mL), acidify the pH to 2–3 with hydrochloric acid, followed by extractions with EtOAc (3 × 5 mL). The combined organic phase was dried with anhydrous sodium sulfate and the solvent was evaporated to obtain compound **7a–7r**.

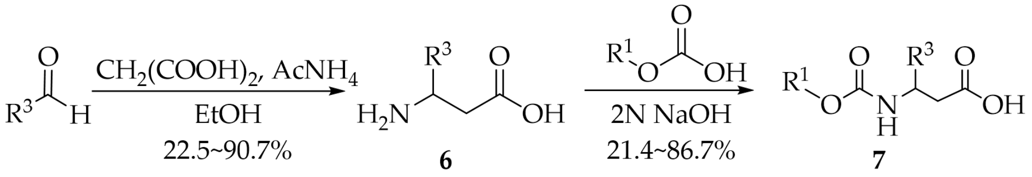

7a: $R^1 = {}^iPr$, $R^3 = H$;
7b: $R^1 = {}^iPr$, $R^3 = Ph$;
7c: $R^1 = {}^iPr$, $R^3 = 2\text{-}ClC_6H_4$;
7d: $R^1 = {}^iPr$, $R^3 = 3\text{-}ClC_6H_4$;
7e: $R^1 = {}^iPr$, $R^3 = 4\text{-}ClC_6H_4$;
7f: $R^1 = {}^iBu$, $R^3 = 4\text{-}ClC_6H_4$;
7g: $R^1 = {}^tBu$, $R^3 = 4\text{-}ClC_6H_4$;
7h: $R^1 = {}^iPr$, $R^3 = 2\text{-}BrC_6H_4$;
7i: $R^1 = {}^iPr$, $R^3 = 3\text{-}BrC_6H_4$;
7j: $R^1 = {}^iPr$, $R^3 = 4\text{-}BrC_6H_4$;
7k: $R^1 = {}^iPr$, $R^3 = 4\text{-}FC_6H_4$;
7l: $R^1 = {}^iPr$, $R^3 = 4\text{-}CH_3C_6H_4$;
7m: $R^1 = {}^iPr$, $R^3 = 4\text{-}CF_3C_6H_4$;
7n: $R^1 = {}^iPr$, $R^3 = 3\text{-}CH_3OC_6H_4$;
7o: $R^1 = {}^iPr$, $R^3 = 2,3\text{-di-}CH_3OC_6H_3$;
7p: $R^1 = {}^iPr$, $R^3 = 3,4\text{-di-}CH_3OC_6H_3$;
7q: $R^1 = {}^iPr$, $R^3 = $ 2-furanyl;
7r: $R^1 = {}^iPr$, $R^3 = $ 2-thiopheneyl

Scheme S1. Synthesis of compounds **7a–7r**.

3-((Isopropoxycarbonyl)amino)propanoic acid (**7a**). Viscous liquid, 58.77% yield. ^1H NMR (400 MHz, DMSO-d_6) δ 12.18 (br s, 1H), 7.0 (s, 1H), 4.81–4.70 (m, 1H), 3.22–3.13 (m, 2H), 2.37–2.18 (m, 2H), 1.14 (d, J = 6.0 Hz, 6H).

3-((Isopropoxycarbonyl)amino)-3-phenylpropanoic acid (**7b**). White solid, 56.61% yield, m.p. 96–98 °C; ^1H NMR (400 MHz, DMSO-d_6) δ 12.36 (br s, 1H), 7.64 (d, J = 8.4 Hz, 1H), 7.31–7.21 (m, 5H), 4.94–4.89 (m, 1H), 4.74–4.65 (m, 1H), 2.68–2.58 (m, 2H), 1.15 (d, J = 5.6 Hz, 3H), 1.13 (d, J = 5.6 Hz, 3H).

3-(2-Chlorophenyl)-3-((isopropoxycarbonyl)amino)propanoic acid (**7c**). White solid, 32.63% yield, m.p. 170–172 °C; ^1H NMR (400 MHz, DMSO-d_6) δ 12.36 (br s, 1H), 7.79 (d, J = 8.4 Hz, 1H), 7.48 (d, J = 5.6 Hz, 1H), 7.40 (d, J = 8.0 Hz, 1H), 7.34 (t, J = 7.2 Hz, 1H), 7.27 (d, J = 7.2 Hz, 1H), 5.36–5.31 (m, 1H), 4.72–4.66 (m, 1H), 2.76–2.58 (m, 2H), 1.15 (d, J = 5.6 Hz, 3H), 1.13 (d, J = 5.6 Hz, 3H).

3-(3-Chlorophenyl)-3-((isopropoxycarbonyl)amino)propanoic acid (**7d**). White solid, 53.28% yield, m.p. 96–98 °C; ^{1}H NMR (400 MHz, DMSO-d_6) δ 12.41 (s, 1H), 7.70 (d, J = 8.4 Hz, 1H), 7.36 (s, 1H), 7.36–7.26 (m,3H), 4.93–4.87 (m, 1H), 4.71–4.66 (m, 1H), 2.69–2.58 (m, 2H), 1.15 (d, J = 5.6 Hz, 3H), 1.11 (d, J = 5.6 Hz, 3H).

3-(4-Chlorophenyl)-3-((isopropoxycarbonyl)amino)propanoic acid (**7e**). White solid, 59.18% yield, m.p. 150–152 °C; ^{1}H NMR (400 MHz, DMSO-d_6) δ 7.71 (d, J = 8.6 Hz, 1H), 7.38 (d, J = 8.8 Hz, 2H), 7.38 (d, J = 8.2 Hz, 2H), 4.92–4.86 (m, 1H), 4.71–4.66 (m, 1H), 2.71–2.56 (m, 2H), 1.15 (d, J = 5.6 Hz, 3H), 1.11 (d, J = 5.6 Hz, 3H).

3-(4-Chlorophenyl)-3-((isobutoxycarbonyl)amino)propanoic acid (**7f**). White solid, 86.68% yield, m.p. 126–129 °C; ^{1}H NMR (400 MHz, DMSO-d_6) δ 12.30 (s, 1H), 7.77 (d, J = 8.3 Hz, 1H), 7.38 (d, J = 8.0 Hz, 2H), 7.33 (d, J = 8.4 Hz, 2H), 4.92–4.86 (m, 1H), 3.70–3.64 (m, 2H), 2.72–2.58 (m, 2H), 1.84–1.76 (m, 1H), 0.85 (d, J = 7.0 Hz, 6H).

3-((Tert-butoxycarbonyl)amino)-3-(4-chlorophenyl)propanoic acid (**7g**). Obtained by commercial purchase.

3-(2-Bromophenyl)-3-((isopropoxycarbonyl)amino)propanoic acid (**7h**). White solid, 24.86% yield, m.p. 135–137 °C; ^{1}H NMR (400 MHz, DMSO-d_6) δ 7.82 (d, J = 8.0 Hz, 1H), 7.57 (d, J = 7.6 Hz, 1H), 7.44 (d, J = 7.2 Hz, 1H), 7.38 (t, J = 7.6 Hz, 1H), 7.18 (t, J = 7.6 Hz, 1H), 5.31–5.25 (m, 1H), 4.71–4.67 (m, 1H), 2.76–2.58 (m, 2H), 1.15 (d, J = 5.6 Hz, 3H), 1.13 (d, J = 5.6 Hz, 3H).

3-(3-Bromophenyl)-3-((isopropoxycarbonyl)amino)propanoic acid (**7i**). White solid, 64.37% yield, m.p. 89–92 °C; ^{1}H NMR (400 MHz, DMSO-d_6) δ 12.36 (br s, 1H), 7.69 (d, J = 8.0 Hz, 1H), 7.50 (s, 1H), 7.43 (d, J = 6.8 Hz, 1H), 7.32–7.28 (m, 2H), 4.91–4.85 (m, 1H), 4.72–4.66 (m, 1H), 2.68–2.58 (m, 2H), 1.14 (d, J = 5.6 Hz, 3H), 1.12 (d, J = 5.6 Hz, 3H).

3-(4-Bromophenyl)-3-((isopropoxycarbonyl)amino)propanoic acid (**7j**). White solid, 21.41% yield, m.p. 122–125 °C; ^{1}H NMR (400 MHz, DMSO-d_6) δ 12.49 (br s, 1H), 7.68 (d, J = 8.0 Hz, 1H), 7.51 (d, J = 8.0 Hz, 2H), 7.26 (d, J = 8.2 Hz, 2H), 4.90–4.84 (m, 1H), 4.72–4.66 (m, 1H), 2.70–2.56 (m, 2H), 1.16 (d, J = 5.6 Hz, 3H), 1.13 (d, J = 5.6 Hz, 3H).

3-(4-Fluorophenyl)-3-((isopropoxycarbonyl)amino)propanoic acid (**7k**). White solid, 38.32% yield, m.p. 125–127 °C; ^{1}H NMR (400 MHz, DMSO-d_6) δ 7.64 (d, J = 8.6 Hz, 1H), 7.33 (t, J = 7.0 Hz, 2H), 7.12 (t, J = 8.7 Hz, 2H), 4.90–4.84 (m, 1H), 4.72–4.67 (m, 1H), 2.70–2.55 (m, 2H), 1.14 (d, J = 5.6 Hz, 3H), 1.09 (d, J = 5.6 Hz, 3H).

3-((Isopropoxycarbonyl)amino)-3-(p-tolyl)propanoic acid (**7l**). White solid, 49.43% yield, m.p. 89–91 °C; ^{1}H NMR (400 MHz, DMSO-d_6) δ 12.40 (br s, 1H), 7.59 (d, J = 8.8 Hz, 1H), 7.18 (d, J = 7.7 Hz, 2H), 7.10 (d, J = 7.5 Hz, 2H), 4.93–4.87 (m, 1H), 4.71–4.64 (m, 1H), 2.68–2.53 (m, 2H), 2.26 (s, 3H), 1.14 (d, J = 6.0 Hz, 3H), 1.10 (d, J = 6.0 Hz, 3H).

3-((Isopropoxycarbonyl)amino)-3-(4-(trifluoromethyl)phenyl)propanoic acid (**7m**). White solid, 62.92% yield, m.p. 130–133 °C; ^{1}H NMR (400 MHz, DMSO-d_6) δ 12.44 (br s, 1H), 7.78 (d, J = 8.6 Hz, 1H), 7.69 (d, J = 7.6 Hz, 2H), 7.53 (d, J = 8.4 Hz, 2H), 5.01–4.95 (m, 1H), 4.72–4.67 (m, 1H), 2.75–2.61 (m, 2H), 1.14 (d, J = 5.6 Hz, 3H), 1.09 (d, J = 5.6 Hz, 3H).

3-((Isopropoxycarbonyl)amino)-3-(3-methoxyphenyl)propanoic acid (**7n**). Yellow viscous liquid, 49.56% yield; ^{1}H NMR (400 MHz, DMSO-d_6) δ 12.13 (br s, 1H), 7.62 (d, J = 8.8 Hz, 1H), 7.22 (t, J = 7.6 Hz, 1H), 6.88 (s, 1H), 6.87 (d, J = 6.8 Hz, 1H), 6.79 (d, J = 8.8 Hz, 1H), 4.92–4.86 (m, 1H), 4.72–4.66 (m, 1H), 3.73 (s, 3H), 2.68–2.55 (m, 2H), 1.14 (d, J = 6.0 Hz, 3H), 1.11 (d, J = 6.0 Hz, 3H).

3-(2,3-Dimethoxyphenyl)-3-((isopropoxycarbonyl)amino)propanoic acid (**7o**). Yellow viscous liquid, 22.52% yield; ^{1}H NMR (400 MHz, DMSO-d_6) δ 12.25 (br s, 1H), 7.55 (d, J = 8.8 Hz, 1H), 7.02 (t, J = 7.6 Hz, 1H), 6.91 (d, J = 7.6 Hz, 1H), 5.33–5.25 (m, 1H), 4.70–4.64 (m, 1H), 3.78 (s, 3H), 3.76 (s, 3H), 2.57–2.40 (m, 2H), 1.15 (d, J = 6.0 Hz, 3H), 1.10 (d, J = 6.0 Hz, 3H).

3-(3,4-Dimethoxyphenyl)-3-((isopropoxycarbonyl)amino)propanoic acid (**7p**). White solid, 81.25% yield, m.p. 84–87 °C; ^{1}H NMR (400 MHz, DMSO-d_6) δ 12.39 (br s, 1H), 7.54 (d, J = 8.8 Hz, 1H), 6.93 (s, 1H), 6.86 (d, J = 8.0 Hz, 1H), 6.80 (d, J = 8.4 Hz, 1H), 4.89–4.83 (m, 1H), 4.70–4.63 (m, 1H), 3.73 (s, 3H), 3.71 (m, 3H), 2.67–2.54 (m, 2H), 1.14 (d, J = 6.0 Hz, 3H), 1.11 (d, J = 6.0 Hz, 3H).

3-(Furan-2-yl)-3-((isopropoxycarbonyl)amino)propanoic acid (**7q**). Grey solid, 29.92% yield, m.p. 82–84 °C; ^{1}H NMR (400 MHz, DMSO-d_6) δ 12.32 (br s, 1H), 7.55 (s, 1H), 7.52 (d, J = 8.8 Hz, 1H), 6.36 (t, J = 2.4 Hz, 1H), 6.17 (d, J = 2.4 Hz, 1H), 5.03–4.97 (m, 1H), 4.79–4.69 (m, 1H), 2.76–2.62 (m, 2H), 1.15 (d, J = 6.0 Hz, 6H).

3-((Isopropoxycarbonyl)amino)-3-(thiophen-2-yl)propanoic acid (**7r**). White solid, 32.18% yield, m.p. 59–62 °C; ^{1}H NMR (400 MHz, DMSO-d_6) δ 12.42 (br s, 1H), 7.69 (d, J = 8.8 Hz, 1H), 7.36 (s, 1H), 6.94 (s, 2H), 5.19–5.13 (m, 1H), 4.73–4.67 (m, 1H), 2.76 (d, J = 7.4 Hz, 2H), 1.14 (d, J = 3.6 Hz, 6H).

Section S2. Detailed bioassay procedures for the in vitro antifungal activities

The fungicidal activities of compounds were evaluated in mycelial growth tests conducted in artificial media against 6 plant pathogens at a rate of 50 μg/mL. Each test compound was dissolved in a suitable amount of acetone and diluted with water containing 0.1% TW-80 to a concentration of 500 μg/mL. To each petri dish was added 1 mL of the test solution and 9 mL of culture medium to make a 50 μg/mL concentration of the test compound, while in another petri dish was added 1 mL distilled water containing 0.1% TW-80 and 9 mL of culture medium as a blank control. A 4 mm diameter of hyphal growth was cut using a hole puncher on a growing fungal culture and the hyphae were moved to the petri dish containing the test compound. Each assay was performed three times. The dishes were stored in controlled environment cabinets (24 ± 1 °C) for 4 days, after which the diameter of mycelial growth was measured and the percentage inhibition was calculated using the following equation: Percentage inhibition (%) = (averaged diameter of mycelia in blank controls—averaged diameter of mycelia in medicated tablets)/(averaged diameter of mycelia in blank controls—4 mm) × 100 [32].

Section S3. Calculation procedures for molecular docking research

The calculation procedures for molecular docking research consist of four steps [33].

Receptor Preparation. The 3D crystal structure of C-14α demethylase (PDB code: 3L4D) was downloaded from the protein data bank (PDB) and this was used as the receptor for molecular docking. Water molecules were removed from the target protein and hydrogen atoms were added using Auto Dock Tools prior to molecular docking.

Ligand preparation. Target compounds are drawn using Chem Office 2015 as ligands followed by management of its conformer and the minimisation process.

Molecular Docking Using Auto Dock Vina. The input files for Auto Dock Vina were prepared using Auto Dock Tools. The protein was placed in a grid box (grid parameters:

center x = 38.01, center y =-33.901, center z = -28.152, size x = 102.63, size y = 76.506, size z = 117.58), using Auto Dock Vina at1.00 Å to define the binding site. The docking procedure was performed using the instructed command prompts.

Analyzing and Output Visualisation using PyMOL. The docking poses were ranked according to their docking scores. The scoring function in Auto Dock was used to predict the binding affinity of one ligand to the receptor molecule. The conformation with the lowest binding affinity was selected for further analysis after the docking process. The docking results included the locations of hydrogen bonds and closely interacting residues were performed by PyMOL software.

References.

32. Zhao, H.P.; Liu, Y.X.; Cui, Z.P.; Beattie, D.; Gu, Y.C.; Wang, Q.M. Design, synthesis, and biological activities of arylmethylamine substituted chlorotriazine and methylthiotriazine compounds. *J. Agric. Food. Chem.* **2011**, *59*, 11711-11717. doi:10.1021/jf203383s.
33. Seyedi, S.S.; Shukri, M.; Hassandarvish, P.; Oo, A.; Shankar, E.M.; Abubakar, S.; Zandi, K. Computational approach towards exploring potential anti-chikungunya activity of selected flavonoids. *Sci. Rep.* **2016**, *6*, 24027. doi:10.1038/srep24027.

Section S4. Figures S1–S66. ^{1}H, and ^{13}C NMR spectra of 2–4, 8–9

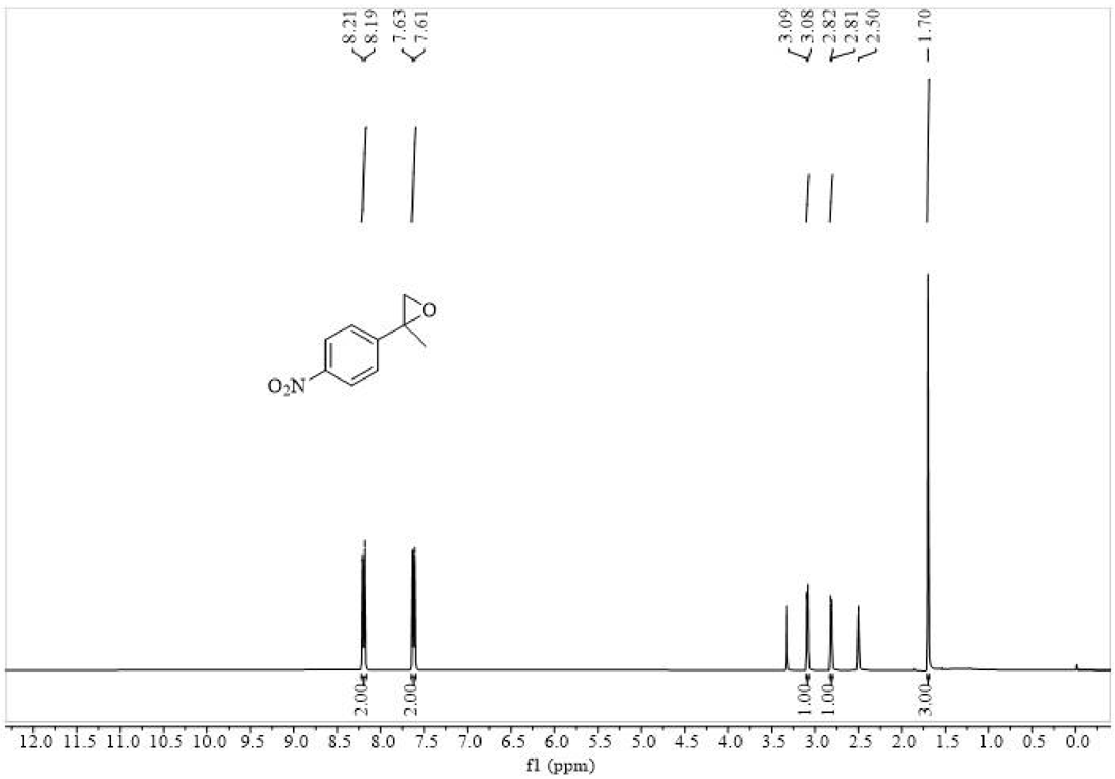

Figure S1. ^{1}H NMR spectrum of **2**

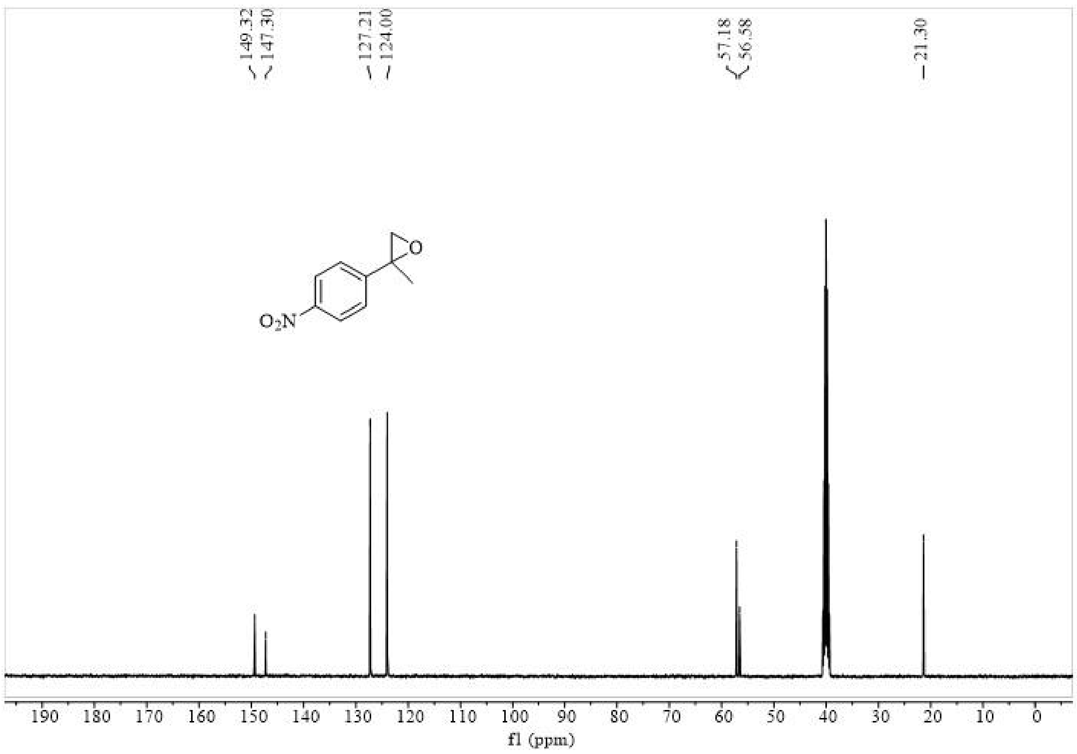

Figure S2. ^{13}C NMR spectrum of **2**

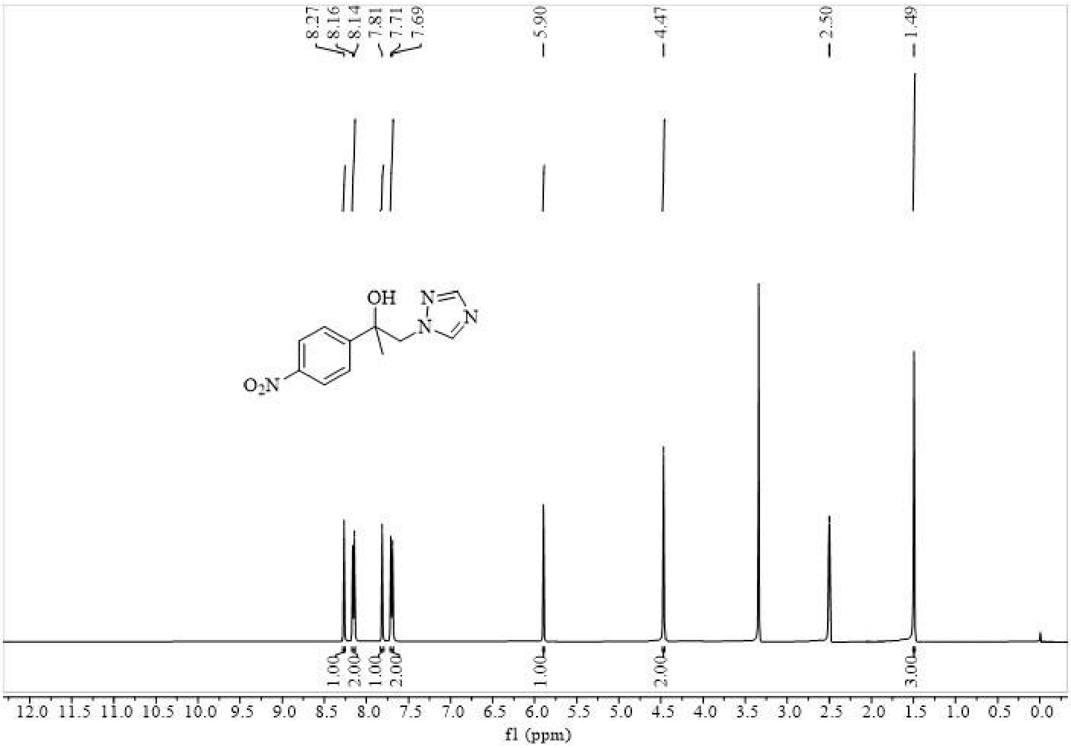

Figure S3. ^{1}H NMR spectrum of **3**

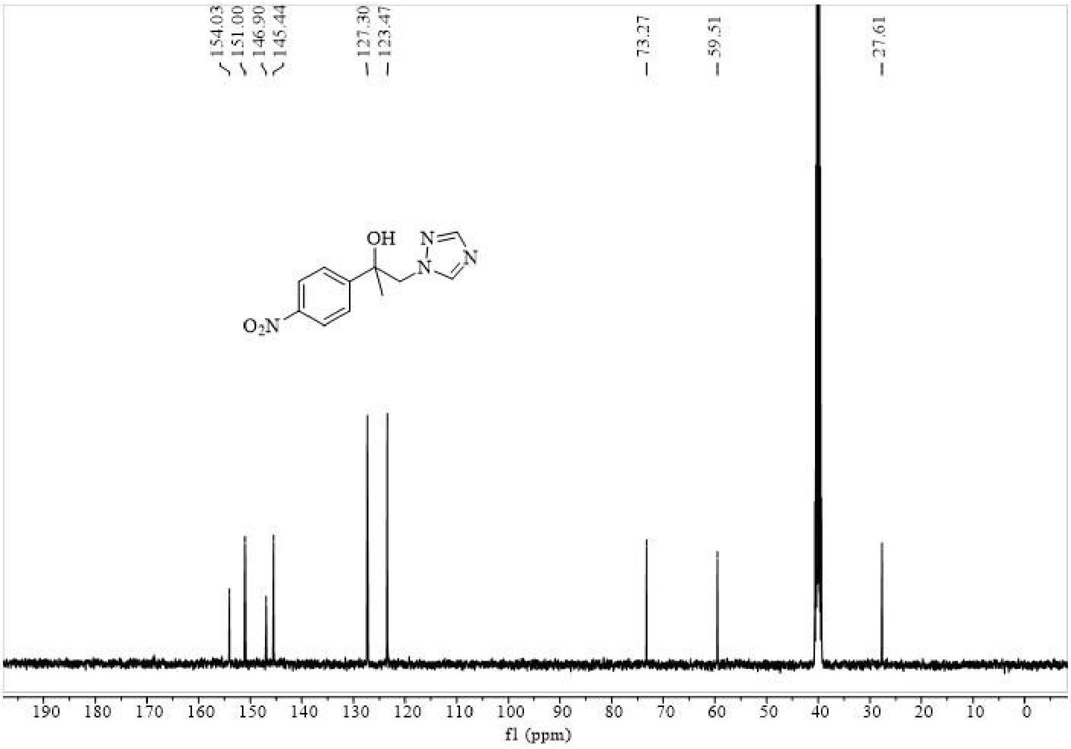

Figure S4. ^{13}C NMR spectrum of **3**

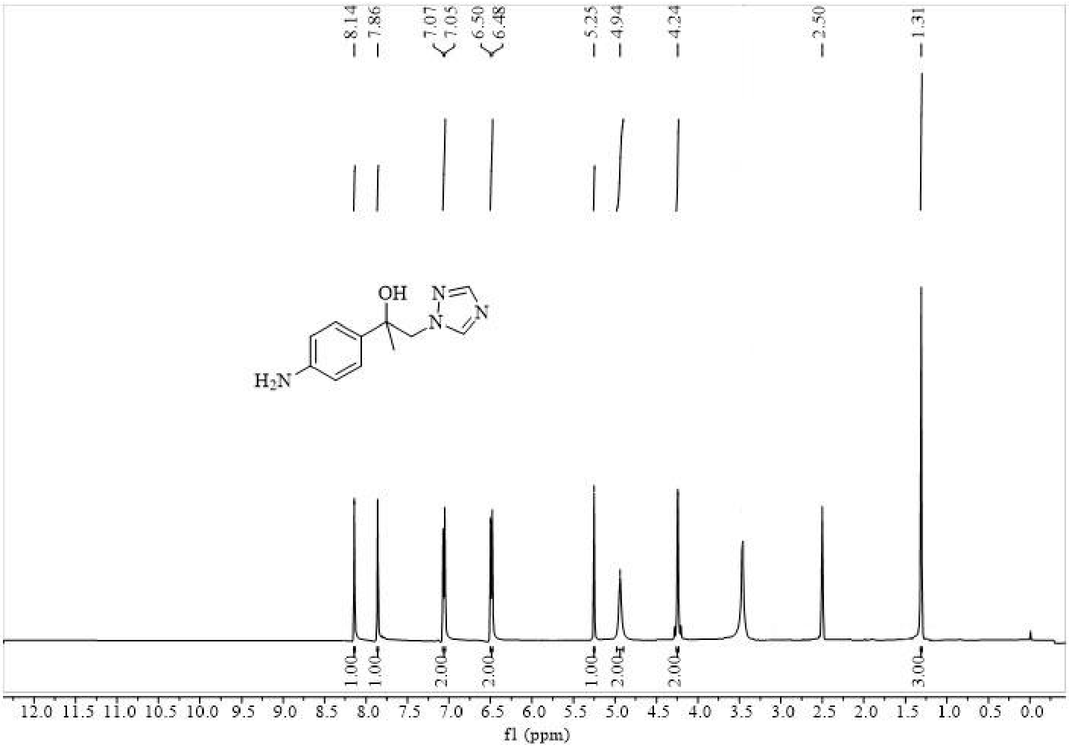

Figure S5. ^{1}H NMR spectrum of **4**

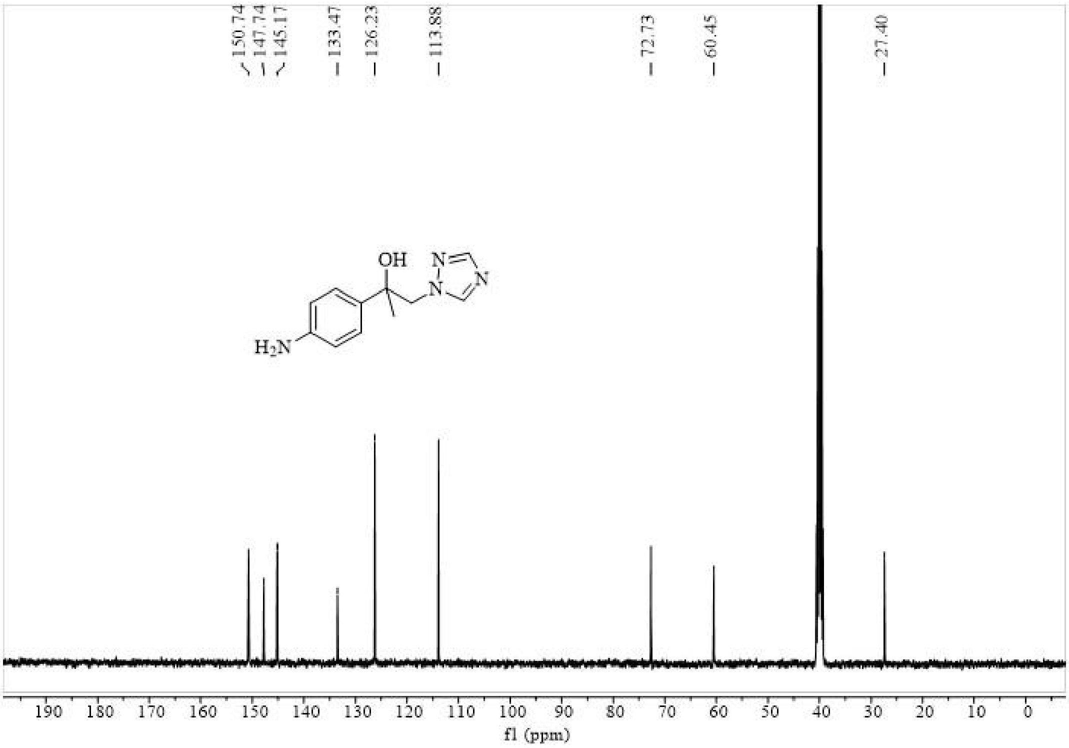

Figure S6. ^{13}C NMR spectrum of **4**

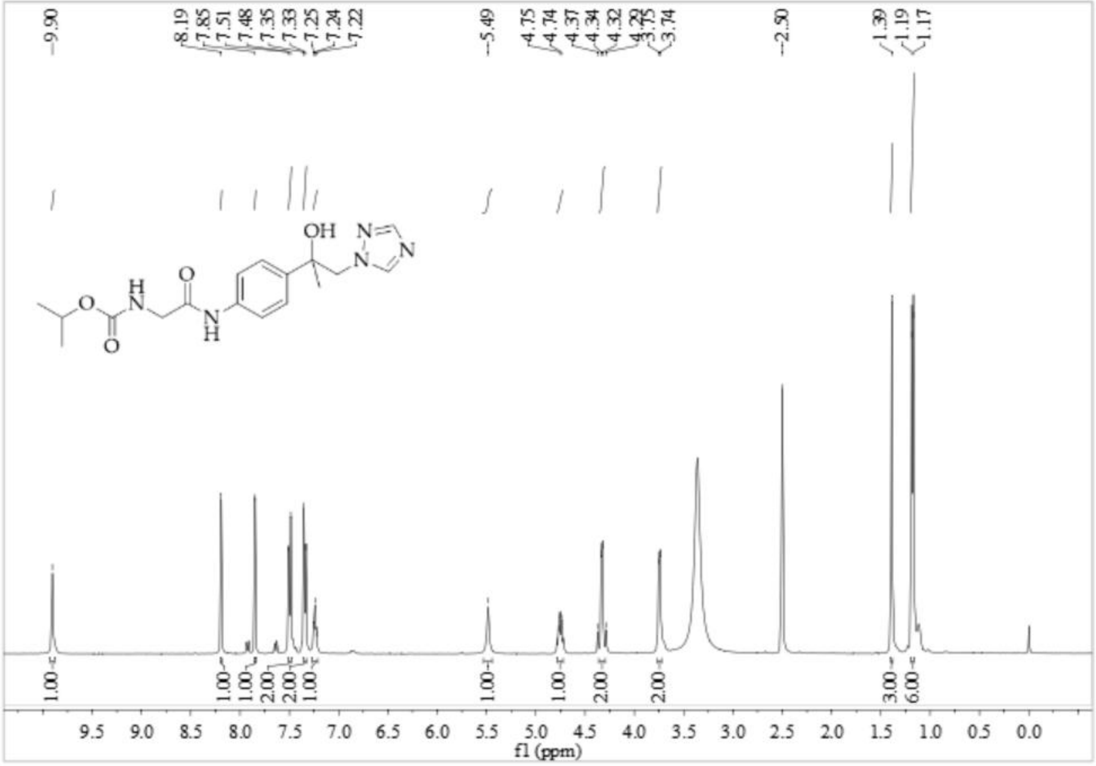

Figure S7. ^{1}H NMR spectrum of **8a**

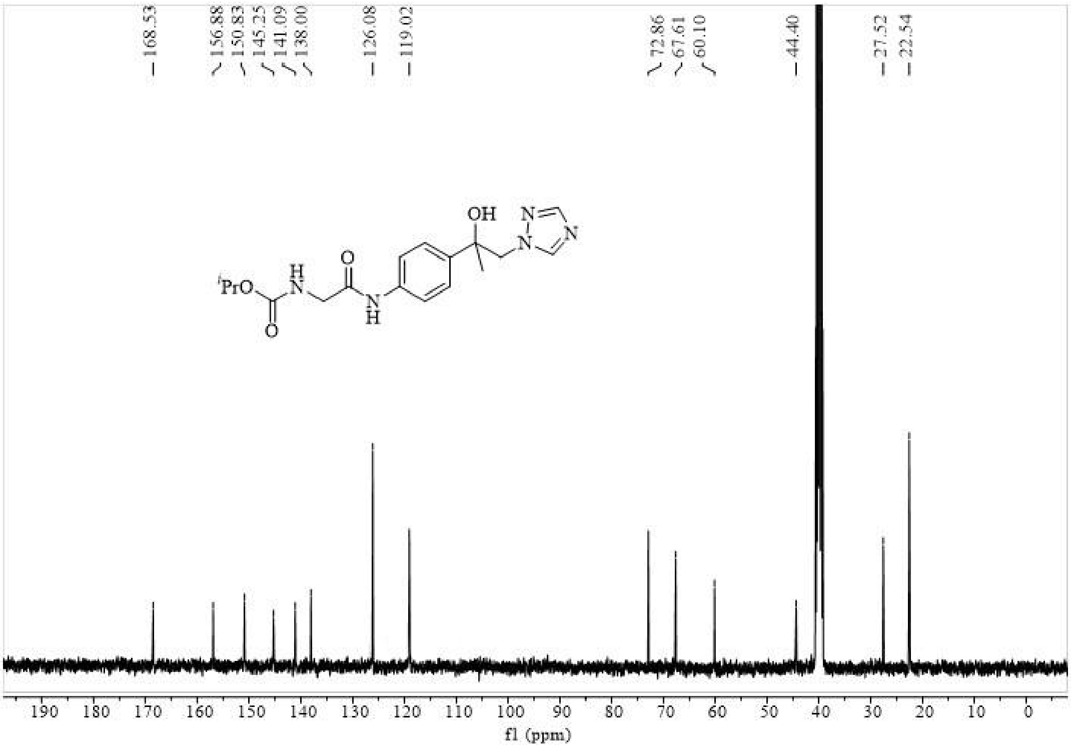

Figure S8. ^{13}C NMR spectrum of **8a**

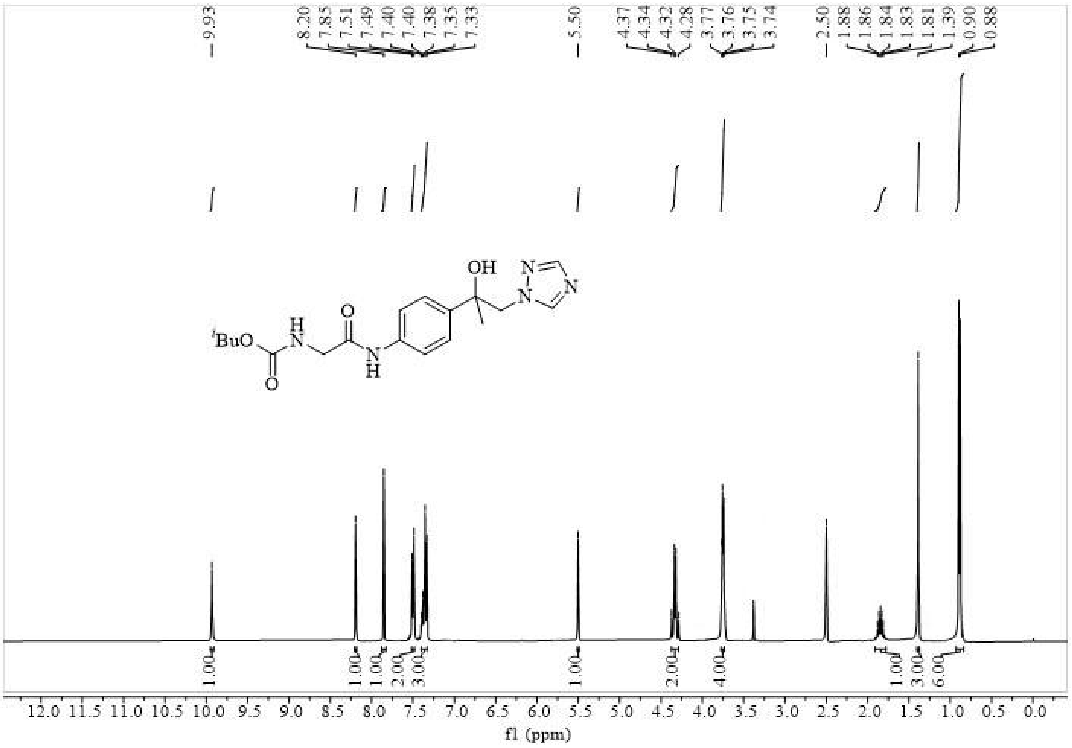

Figure S9. ^{1}H NMR spectrum of **8b**

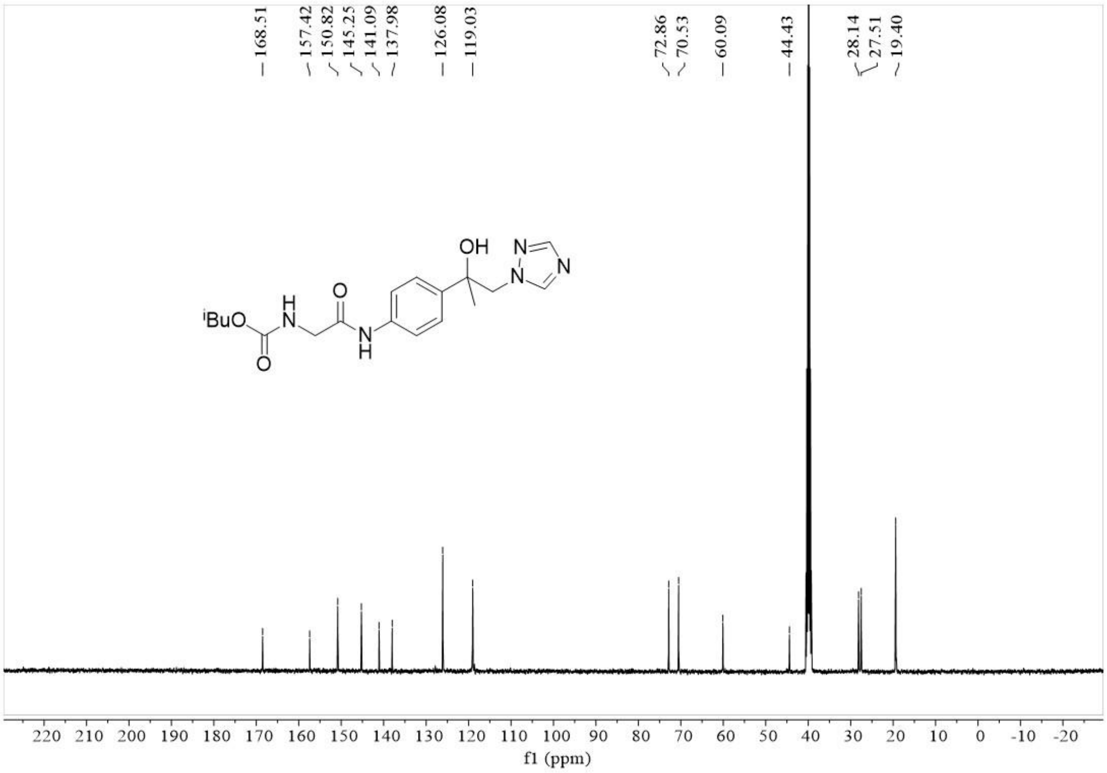

Figure S10. ^{13}C NMR spectrum of **8b**

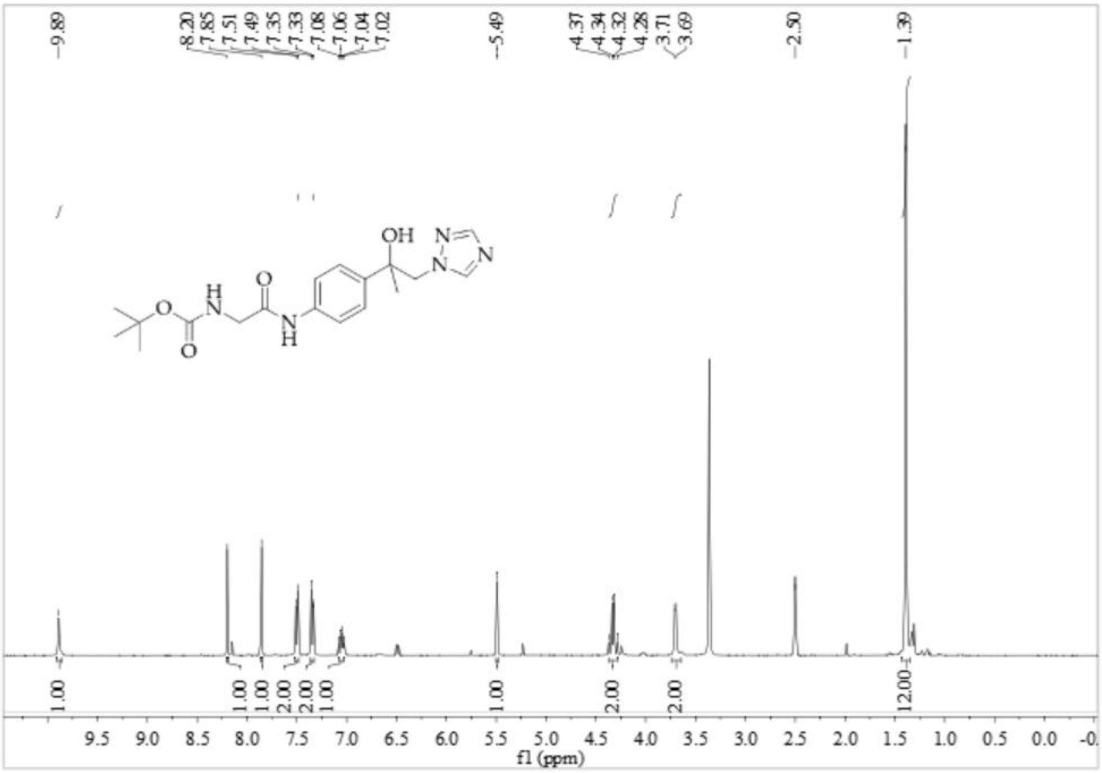

Figure S11. ^{1}H NMR spectrum of **8c**

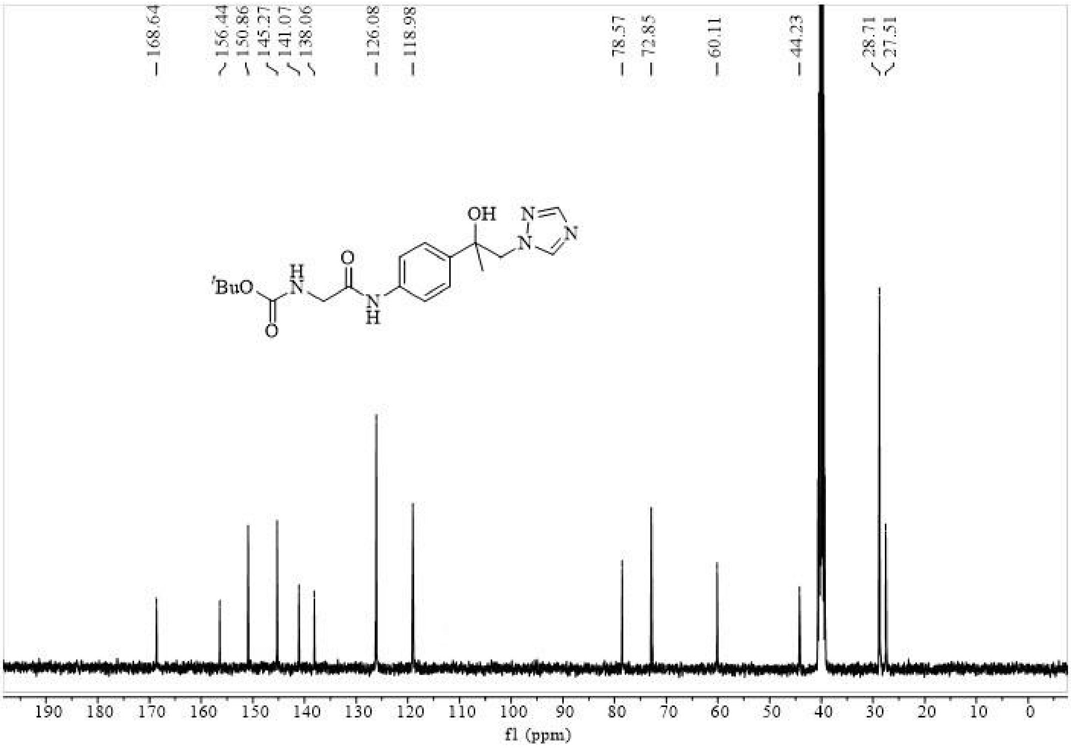

Figure S12. ^{13}C NMR spectrum of **8c**

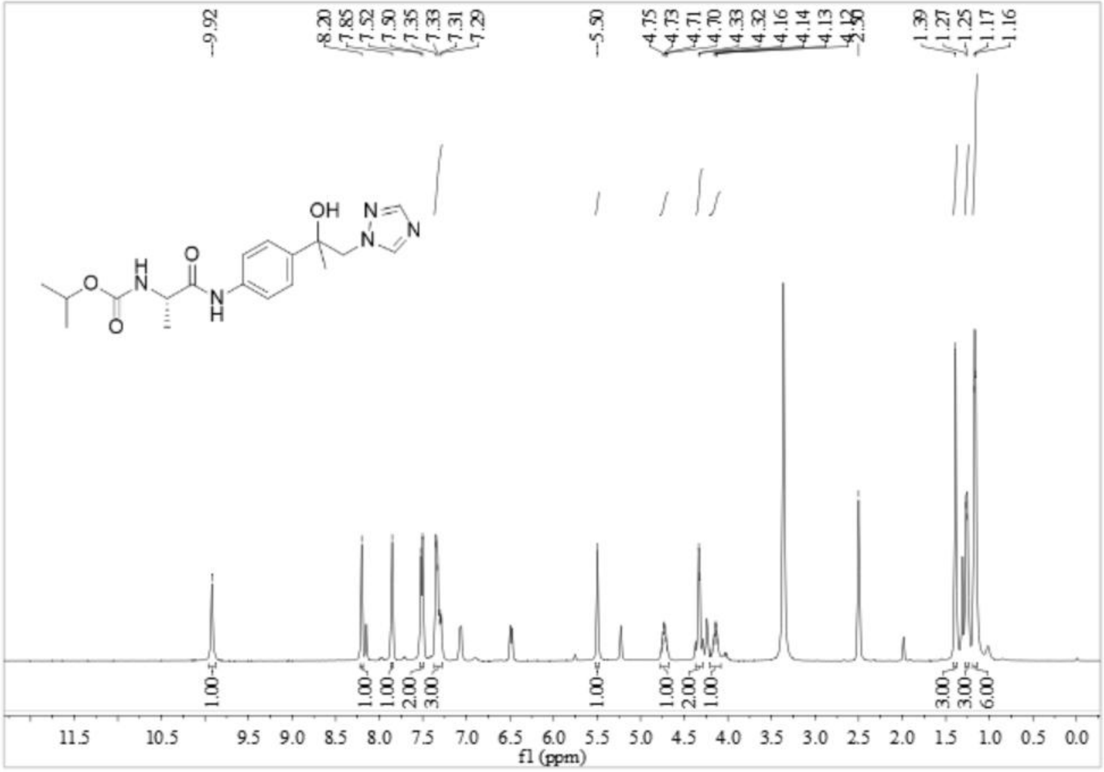

Figure S13. ^{1}H NMR spectrum of **8d**

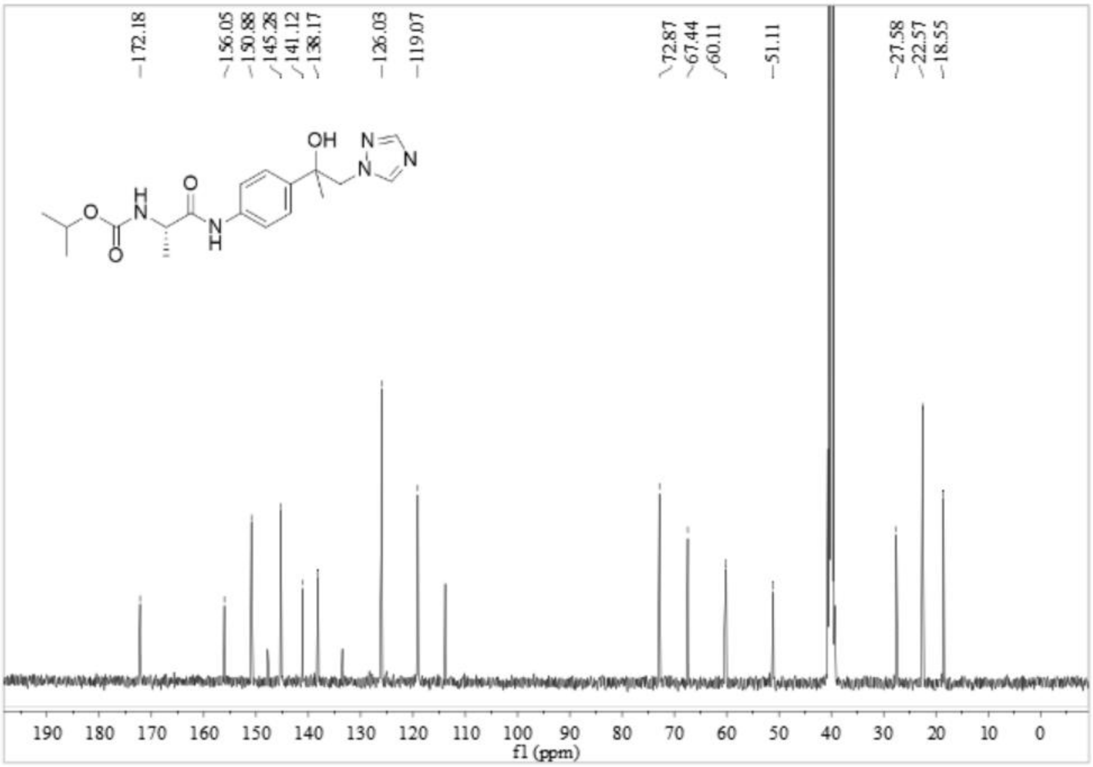

Figure S14. ^{13}C NMR spectrum of **8d**

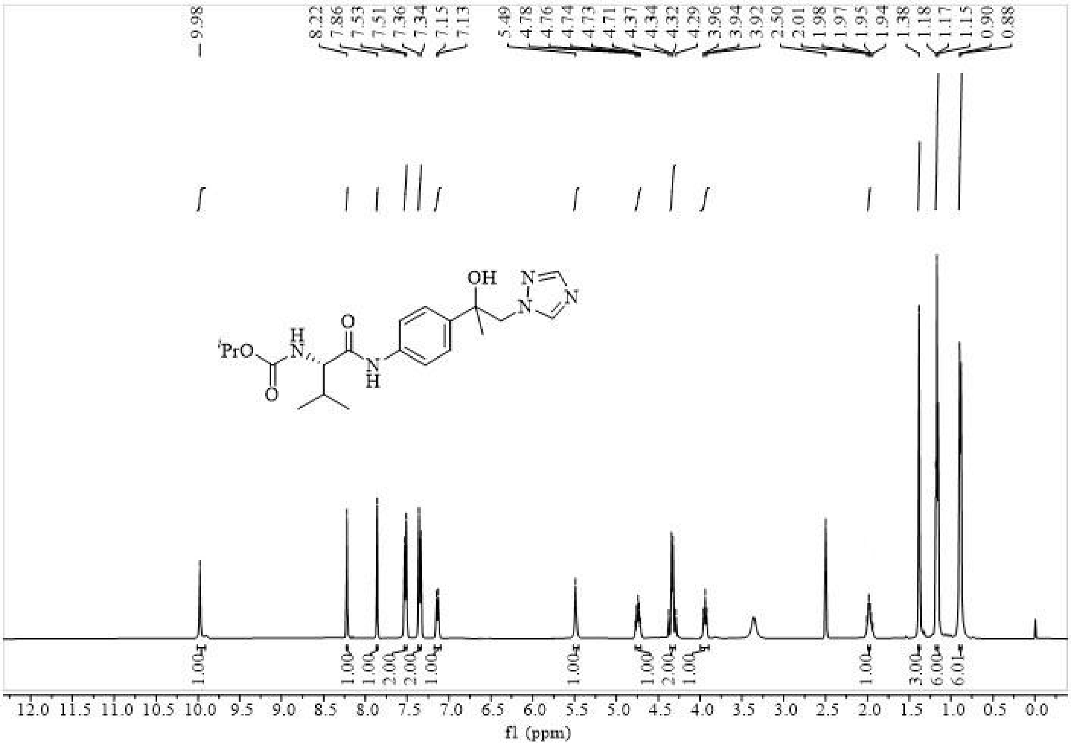

Figure S15. ^{1}H NMR spectrum of **8e**

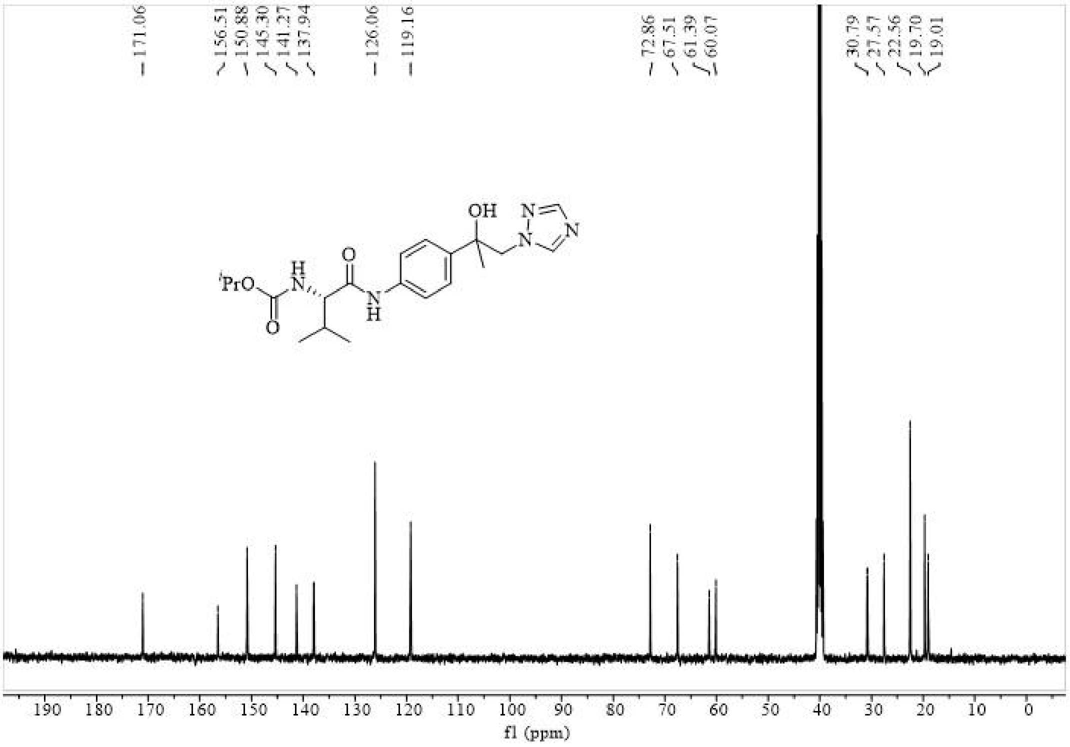

Figure S16. ^{13}C NMR spectrum of **8e**

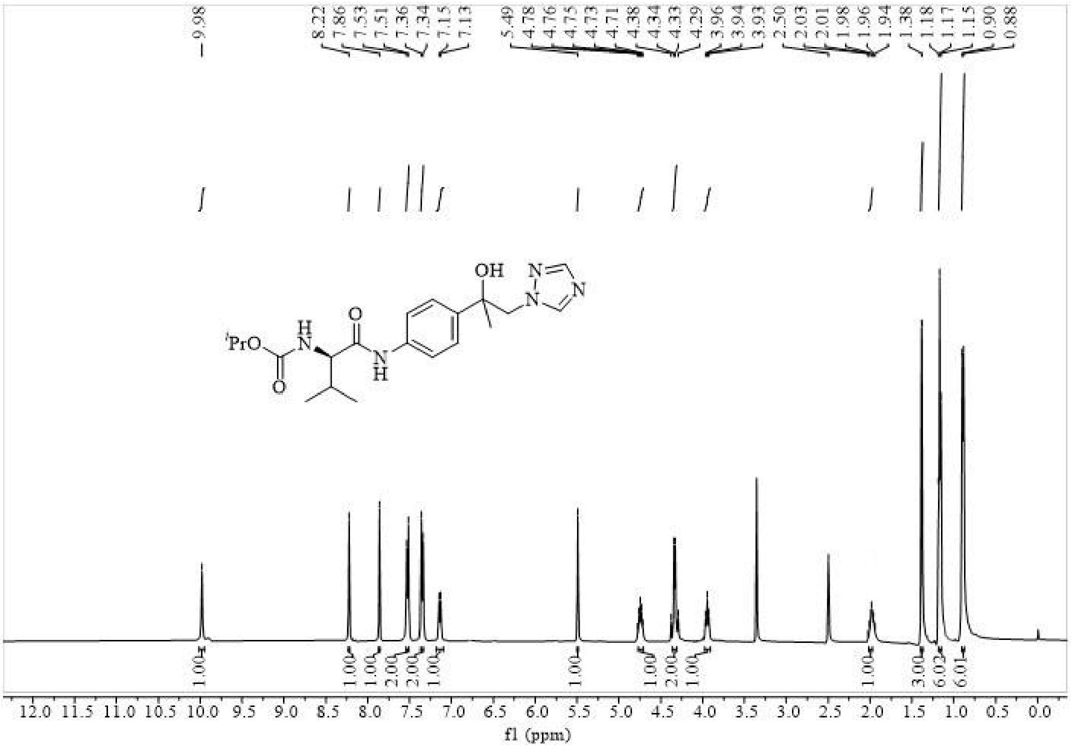

Figure S17. ^{1}H NMR spectrum of **8f**

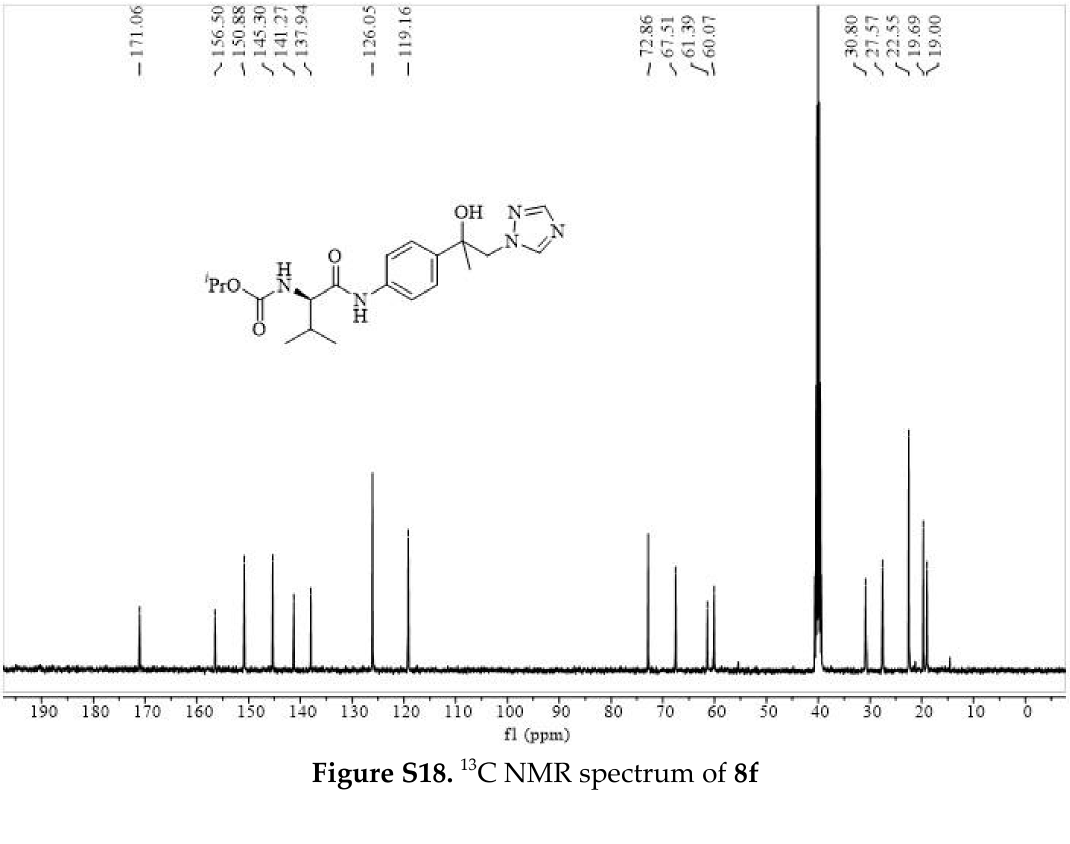

Figure S18. ^{13}C NMR spectrum of **8f**

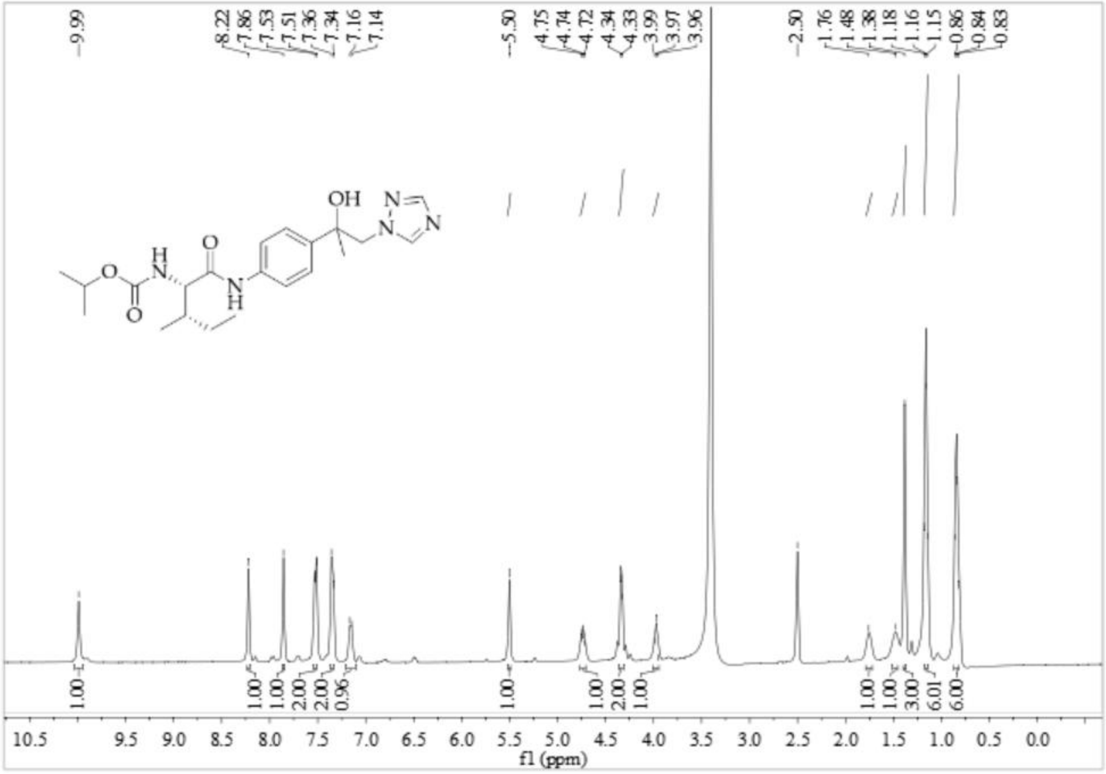

Figure S19. ^{1}H NMR spectrum of **8g**

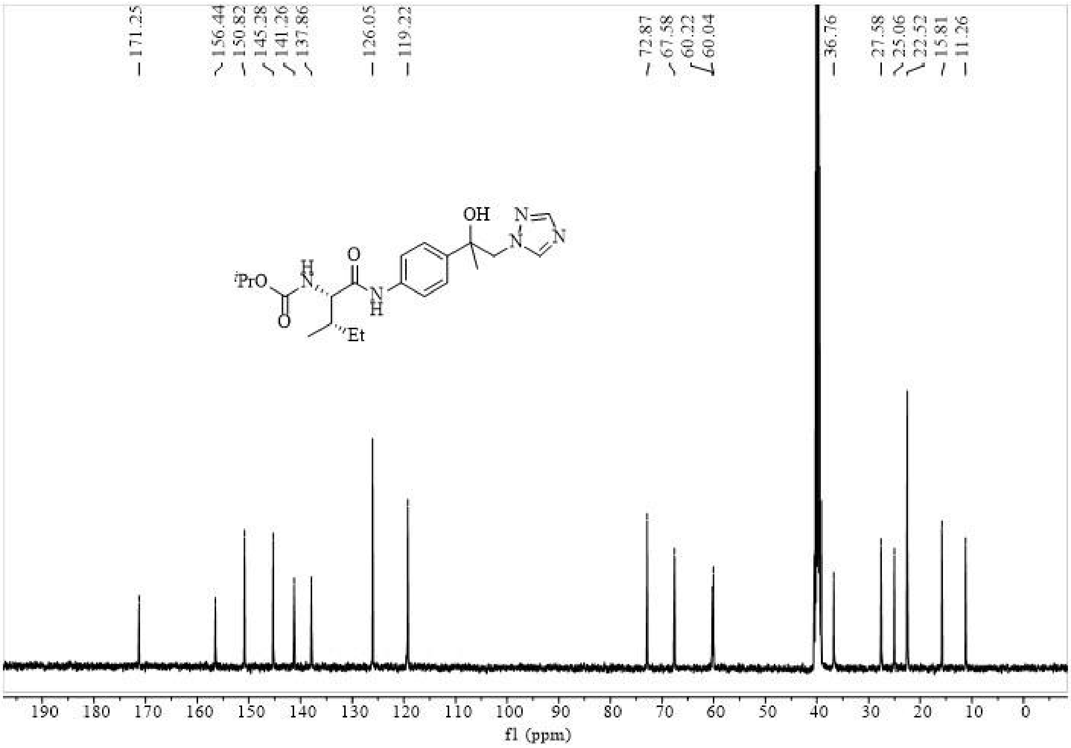

Figure S20. ^{13}C NMR spectrum of **8g**

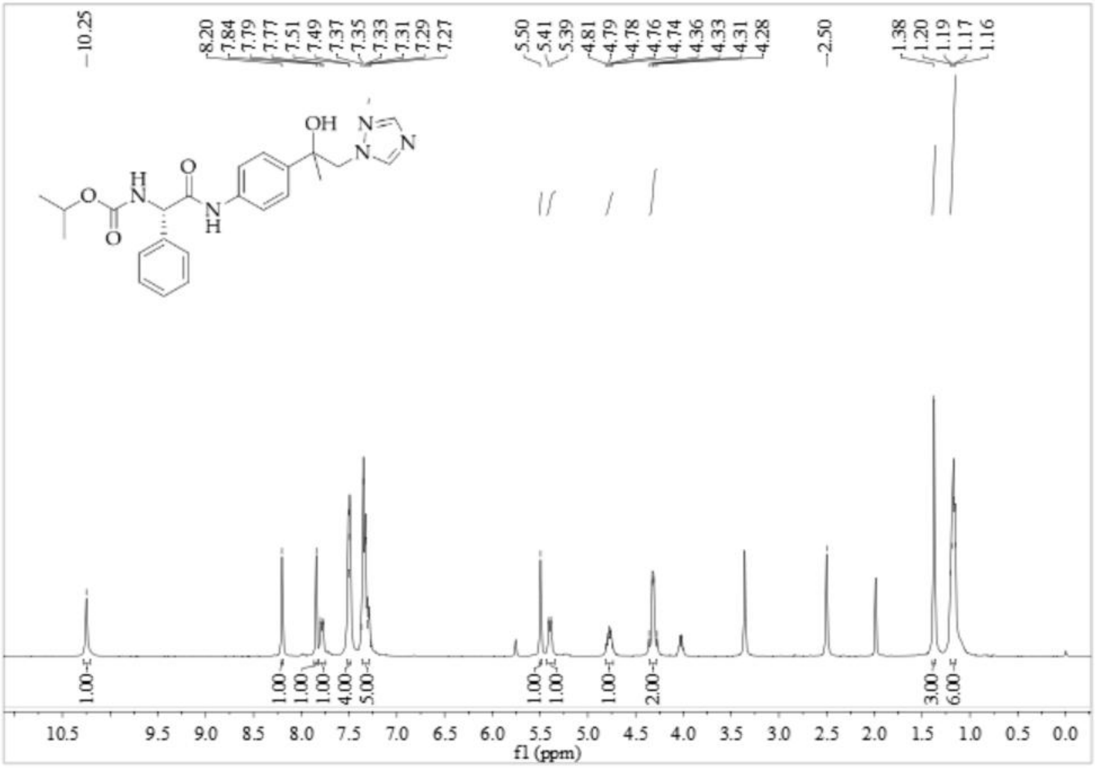

Figure S21. ^{1}H NMR spectrum of **8h**

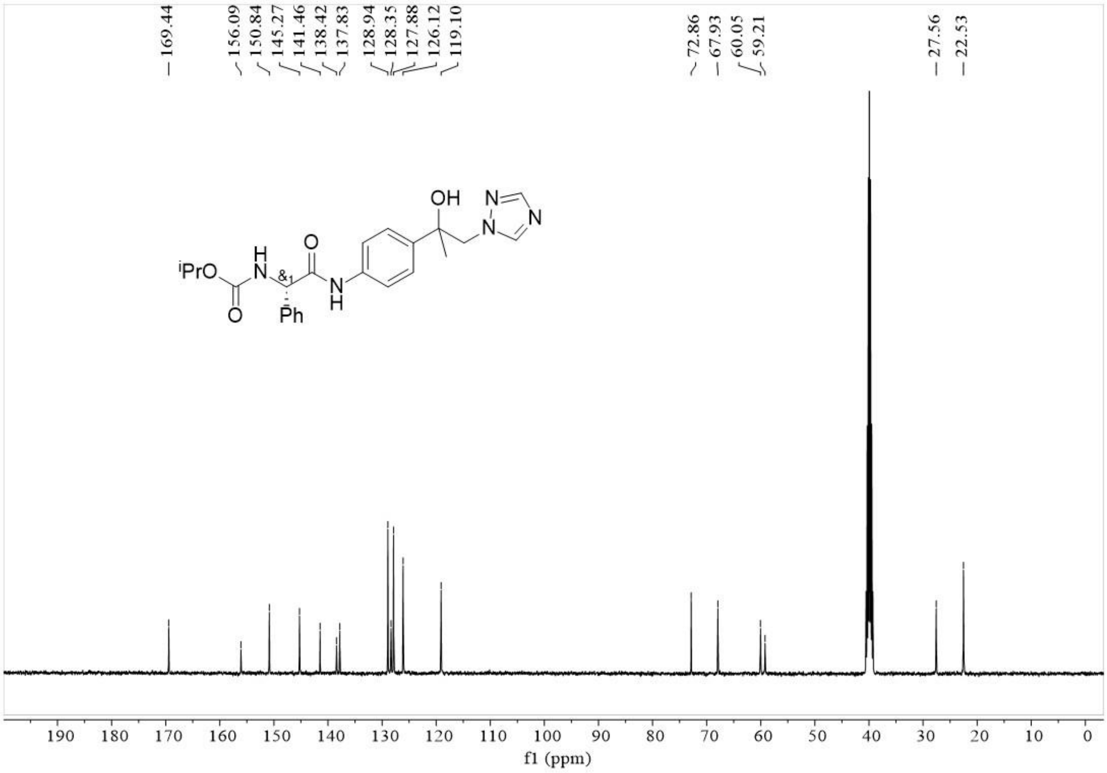

Figure S22. ^{13}C NMR spectrum of **8h**

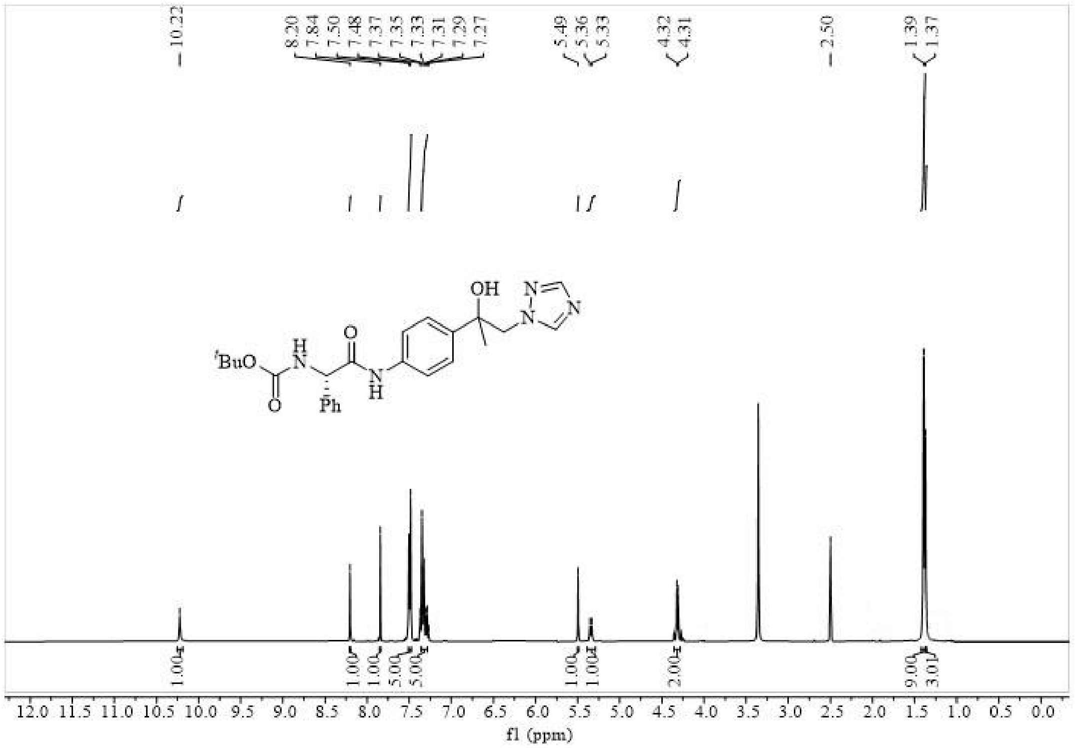

Figure S23. ^{1}H NMR spectrum of **8i**

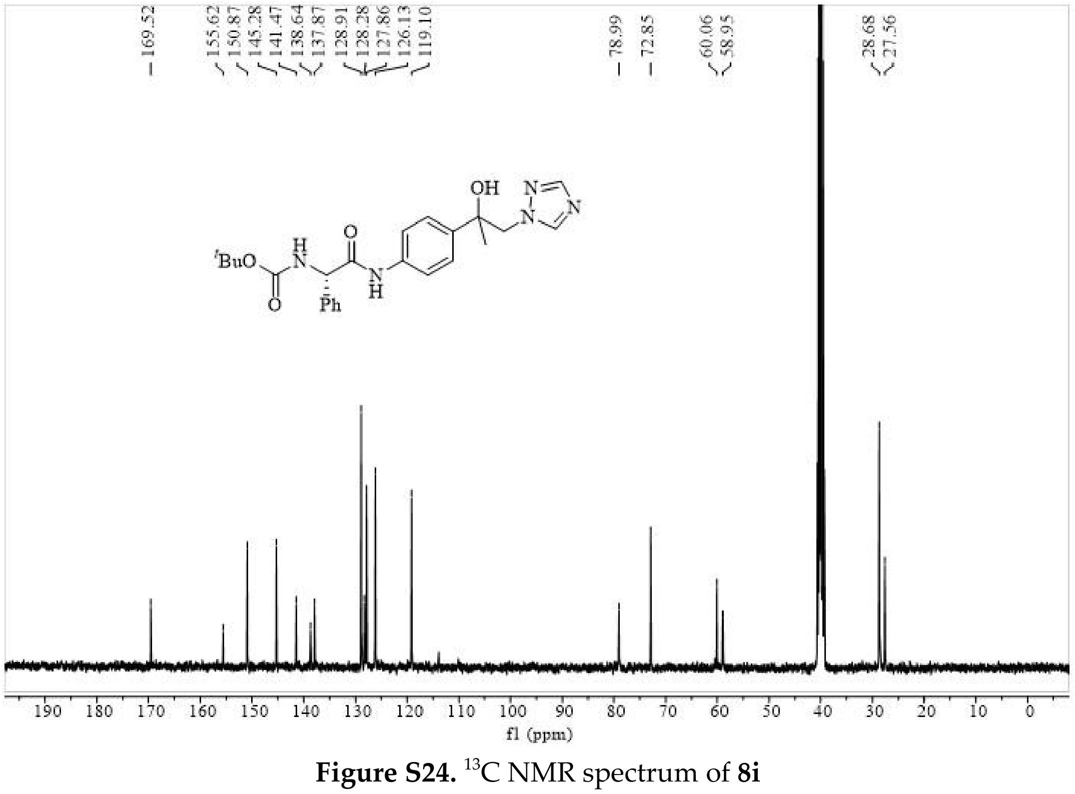

Figure S24. ^{13}C NMR spectrum of **8i**

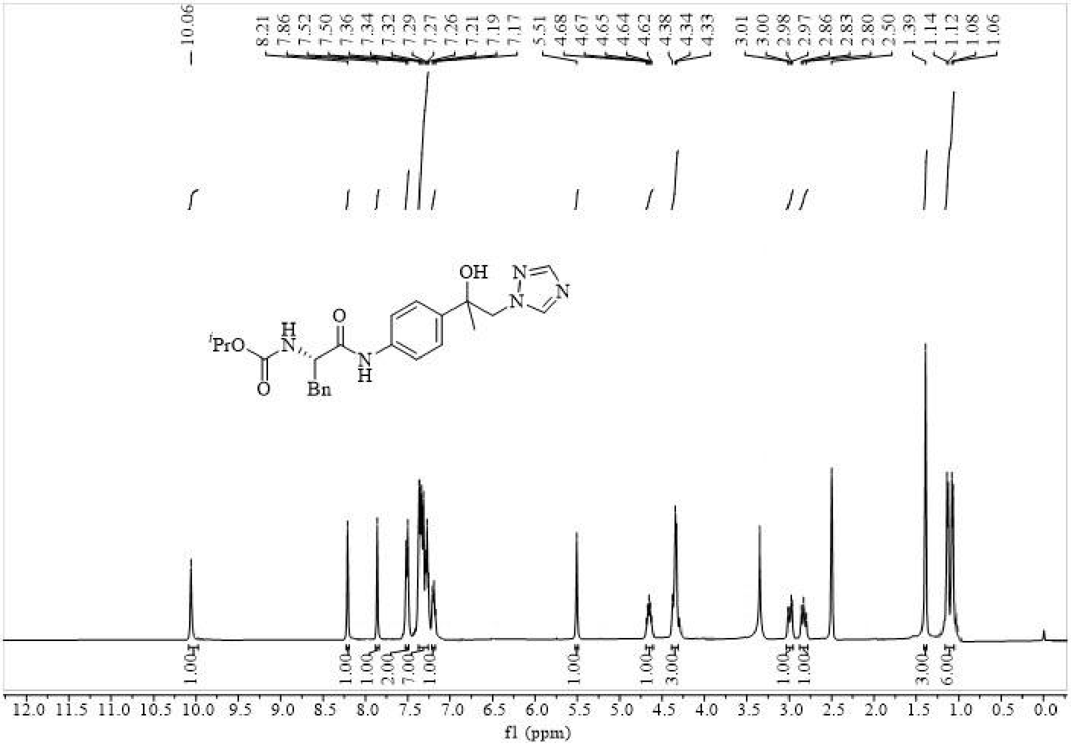

Figure S25. ^{1}H NMR spectrum of **8j**

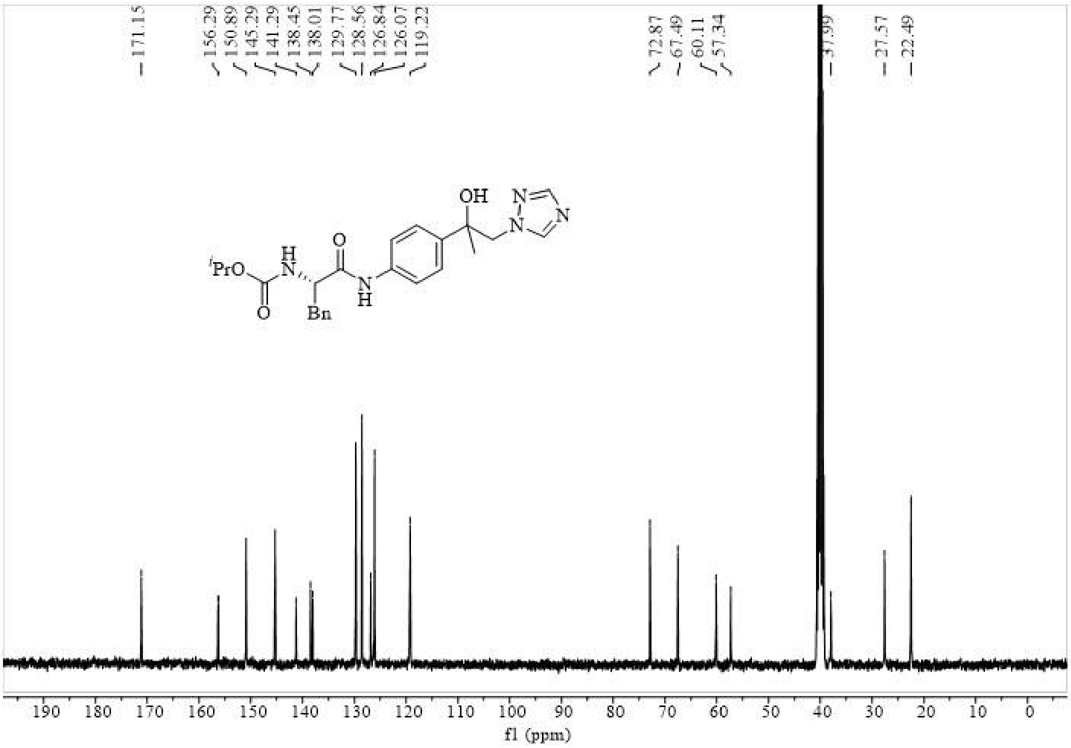

Figure S26. ^{13}C NMR spectrum of **8j**

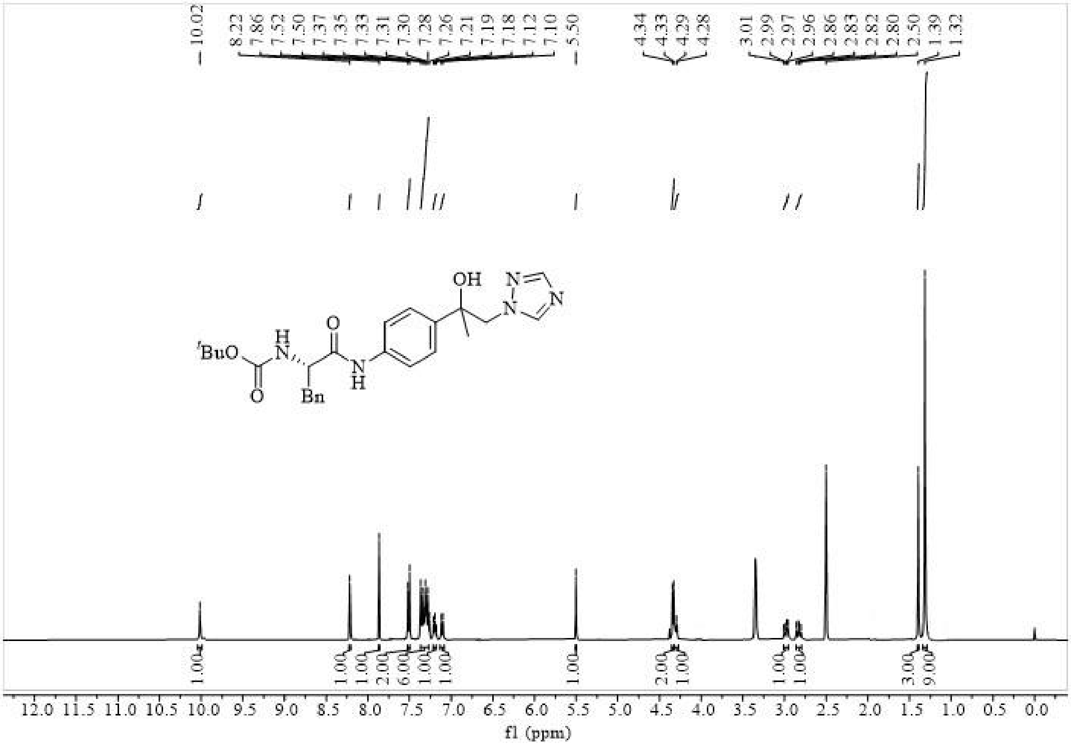

Figure S27. ^{1}H NMR spectrum of **8k**

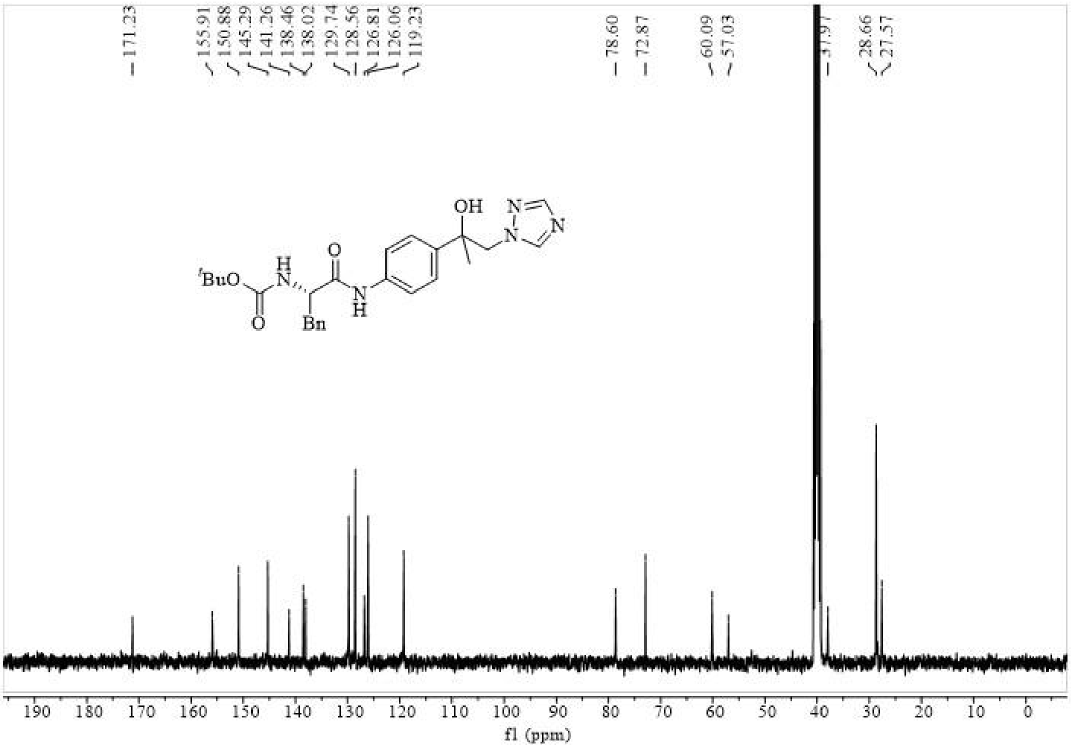

Figure S28. ^{13}C NMR spectrum of **8k**

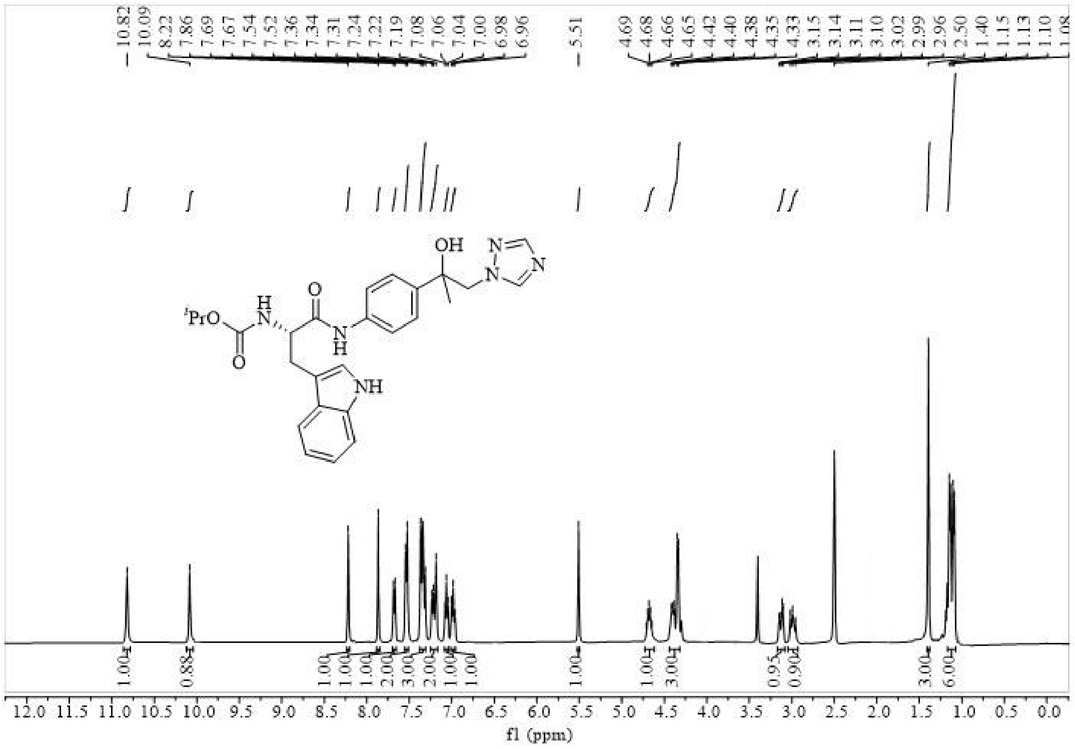

Figure S29. ^{1}H NMR spectrum of **8l**

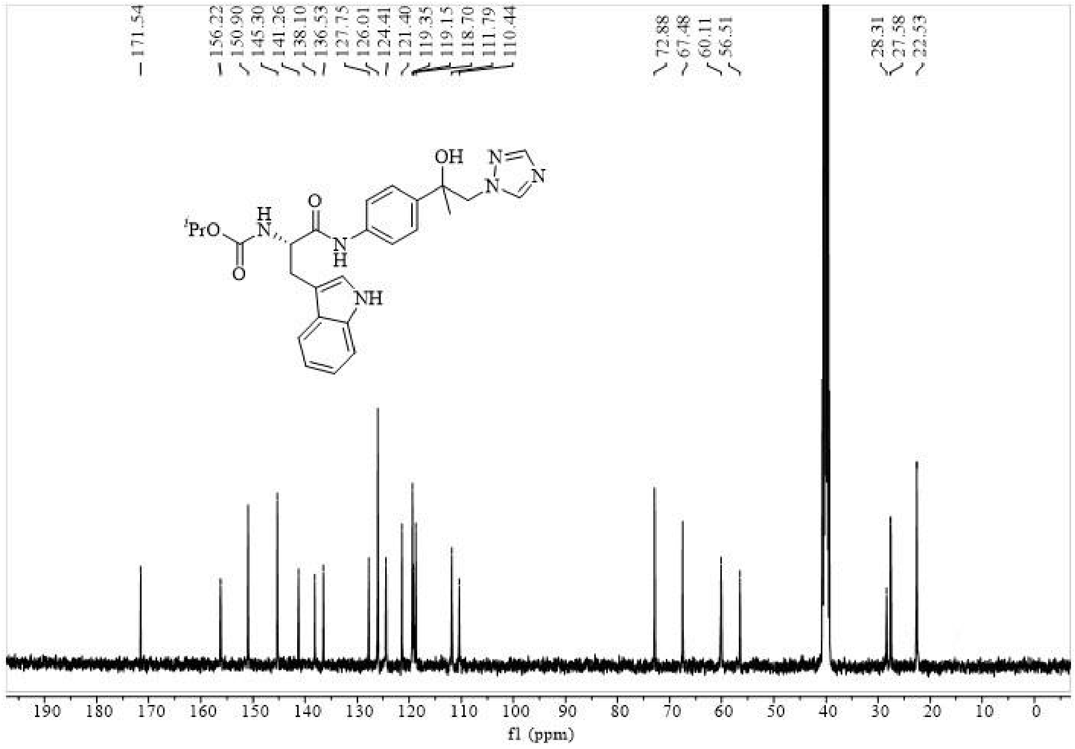

Figure S30. ^{13}C NMR spectrum of **8l**

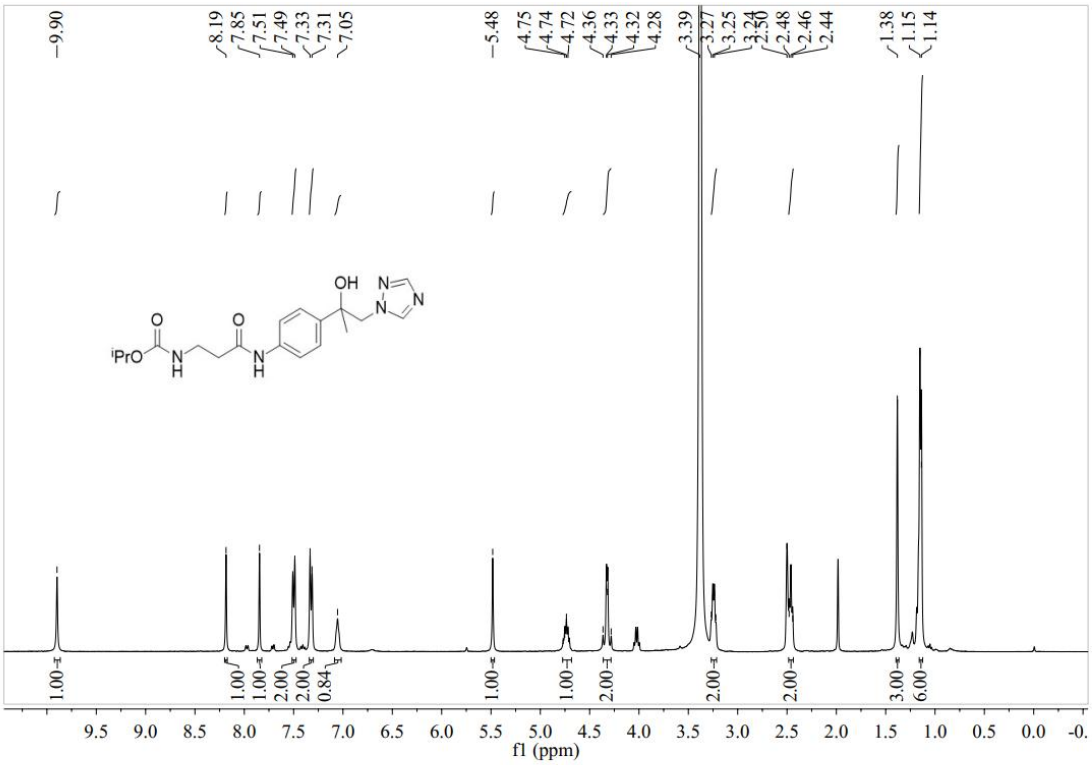

Figure S31. ^{1}H NMR spectrum of **9a**

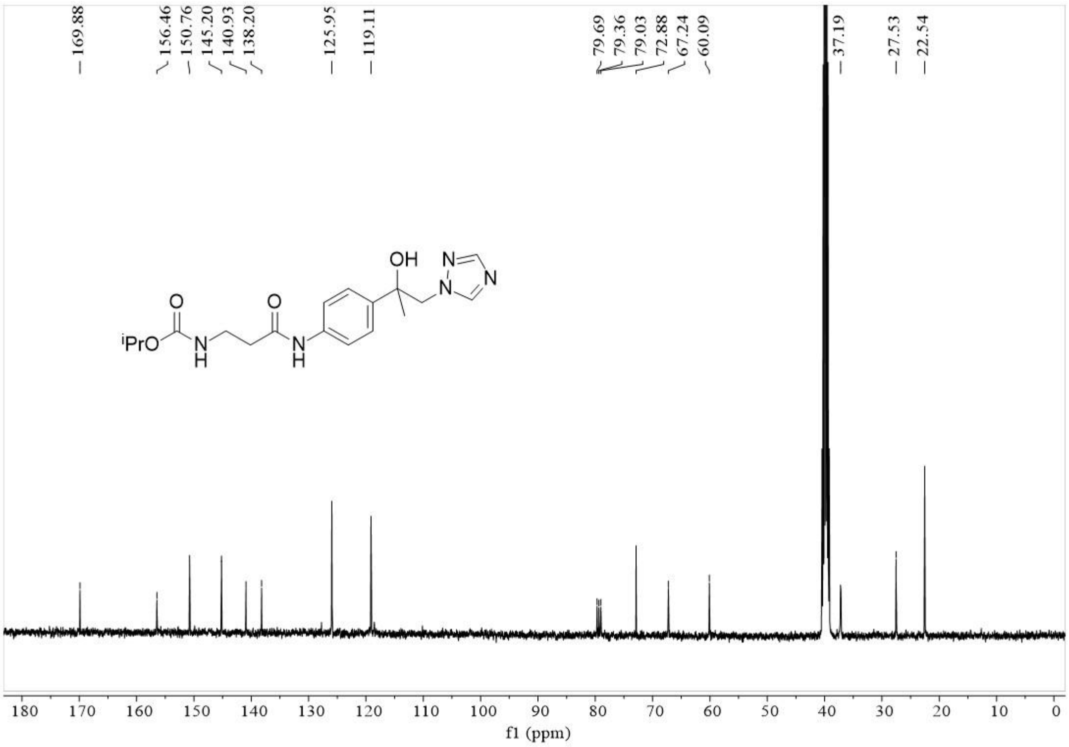

Figure S32. ^{13}C NMR spectrum of **9a**

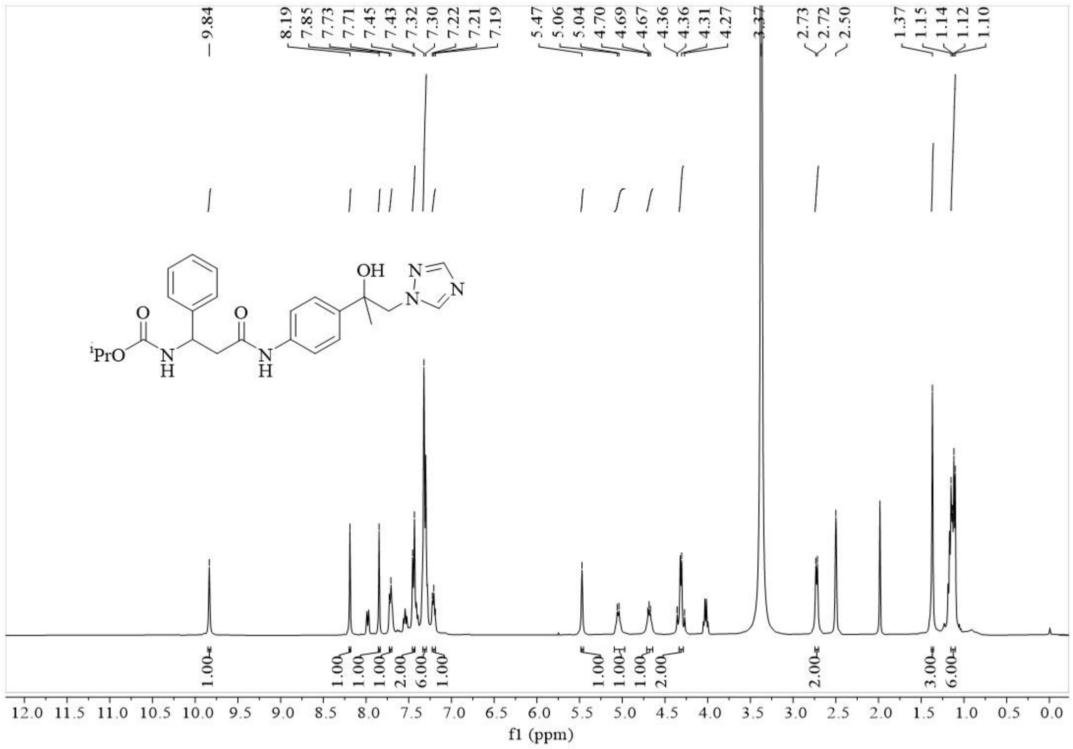

Figure S33. ^{1}H NMR spectrum of **9b**

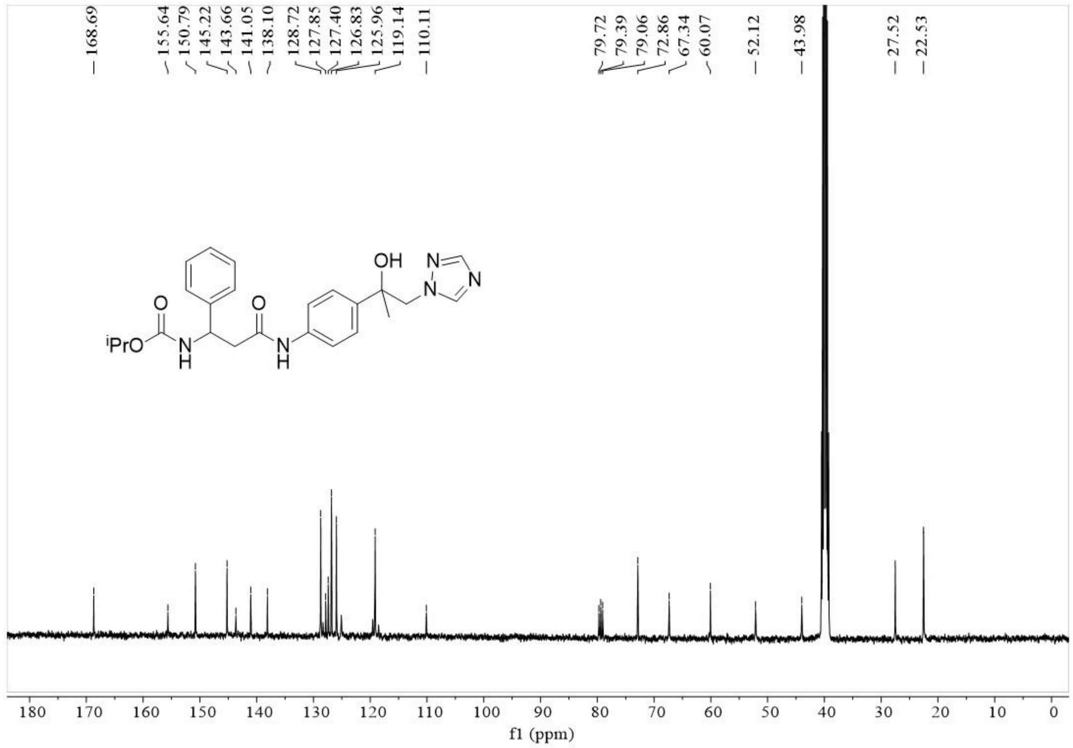

Figure S34. ^{13}C NMR spectrum of **9b**

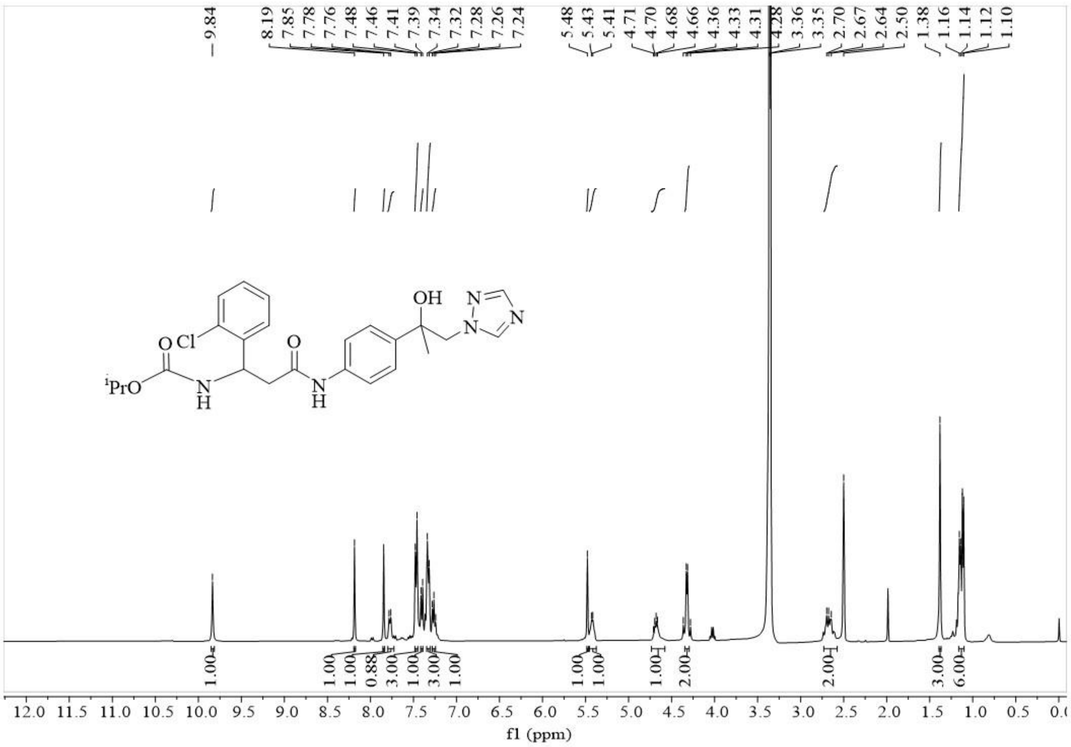

Figure S35. ^{1}H NMR spectrum of **9c**

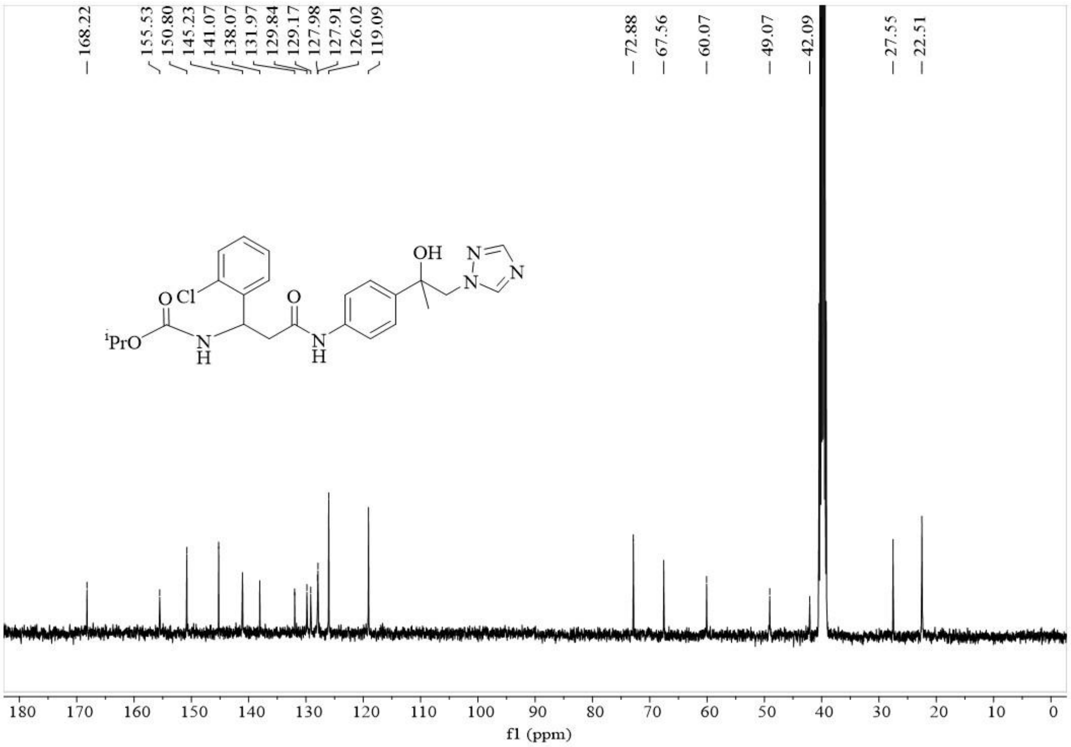

Figure S36. ^{13}C NMR spectrum of **9c**

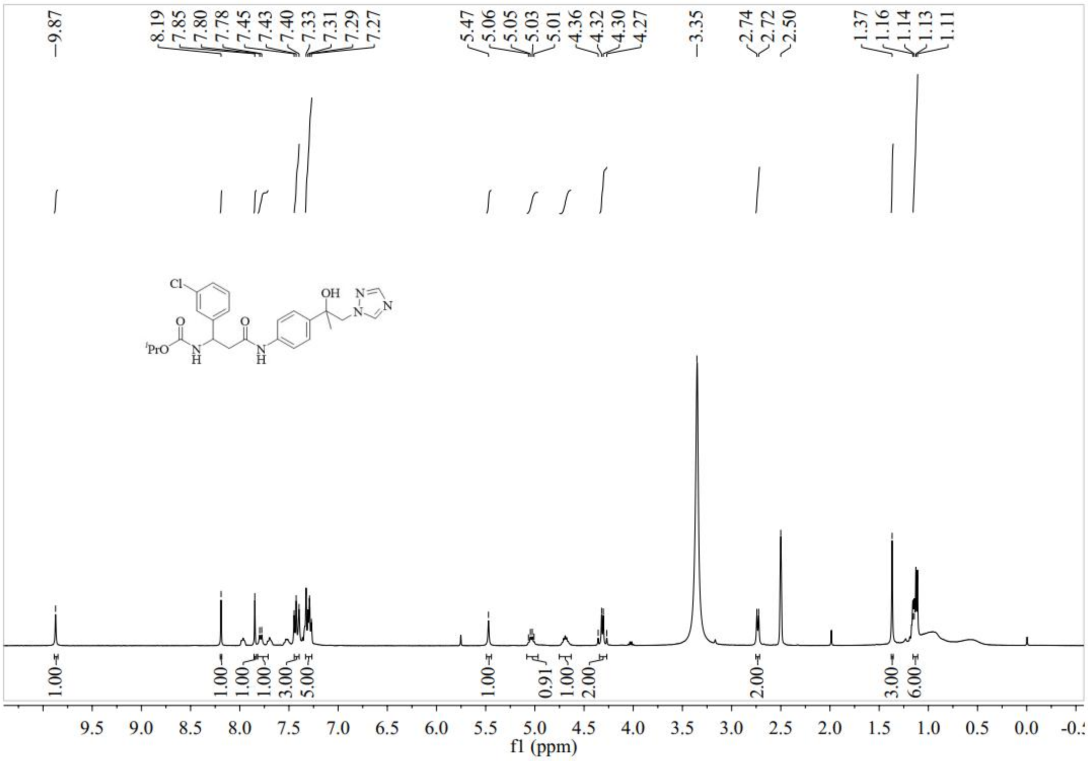

Figure S37. ^{1}H NMR spectrum of **9d**

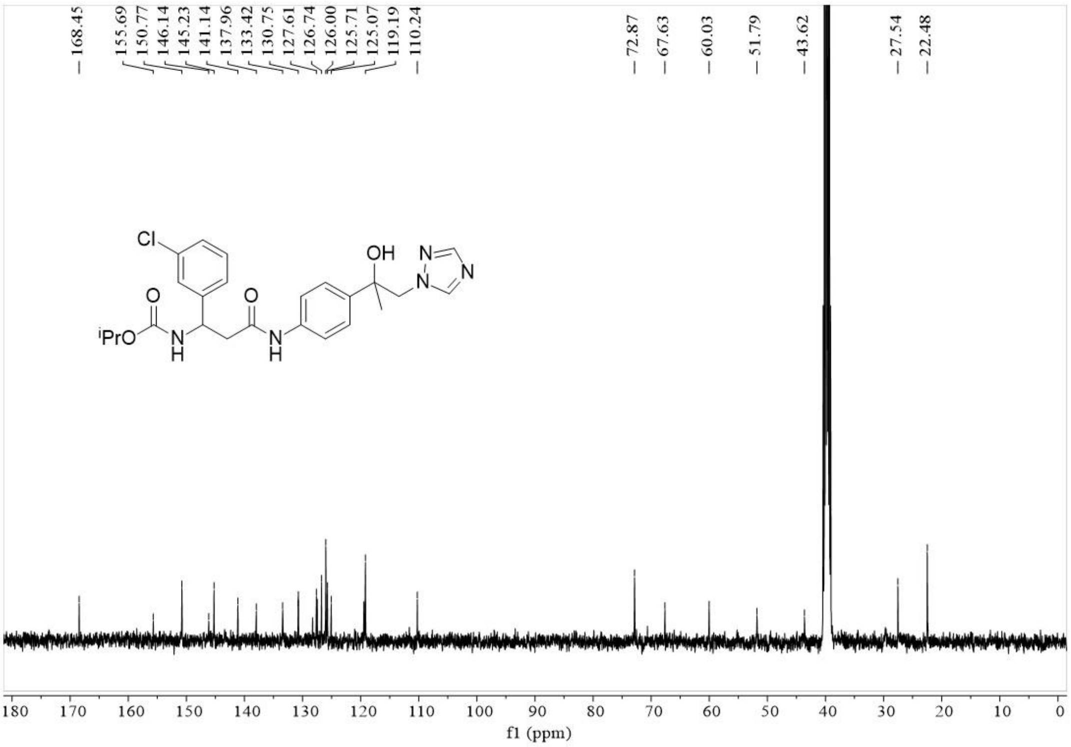

Figure S38. ^{13}C NMR spectrum of **9d**

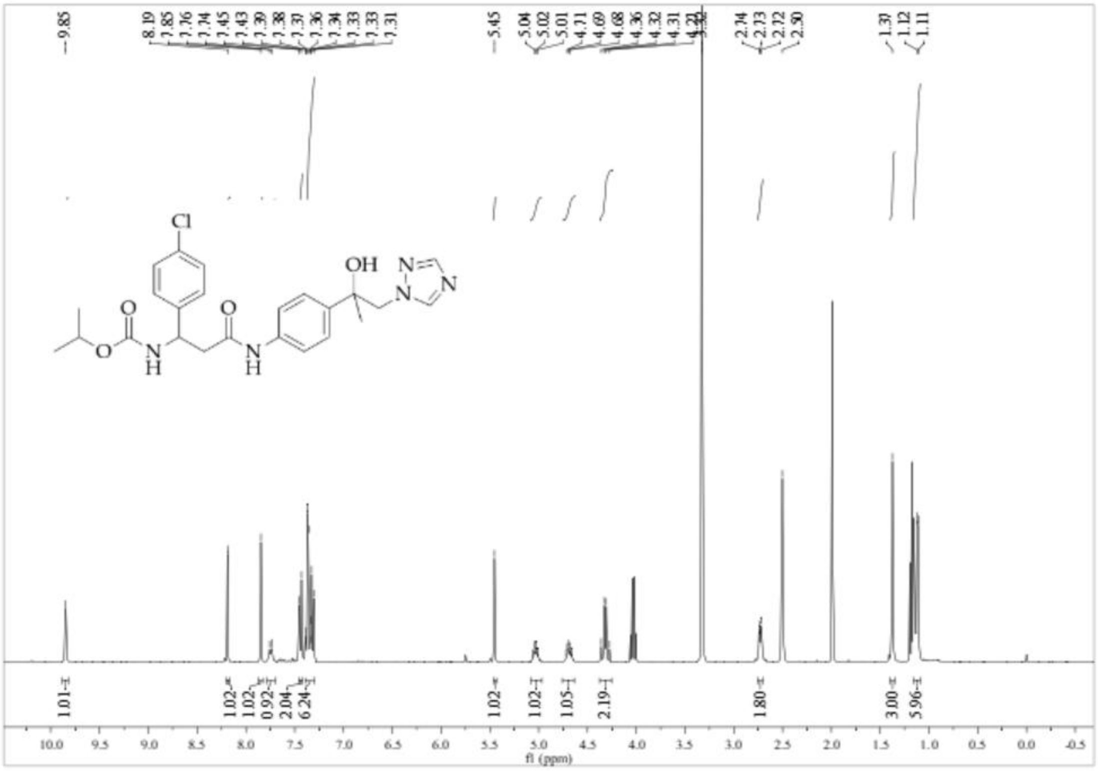

Figure S39. ^{1}H NMR spectrum of **9e**

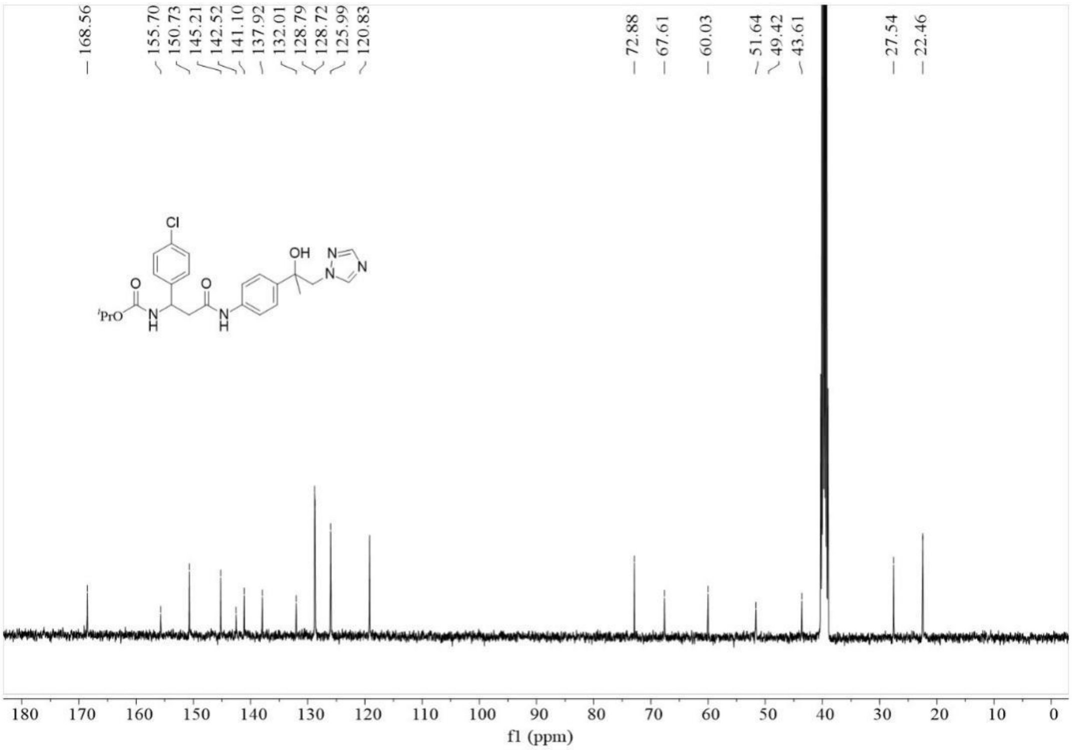

Figure S40. ^{13}C NMR spectrum of **9e**

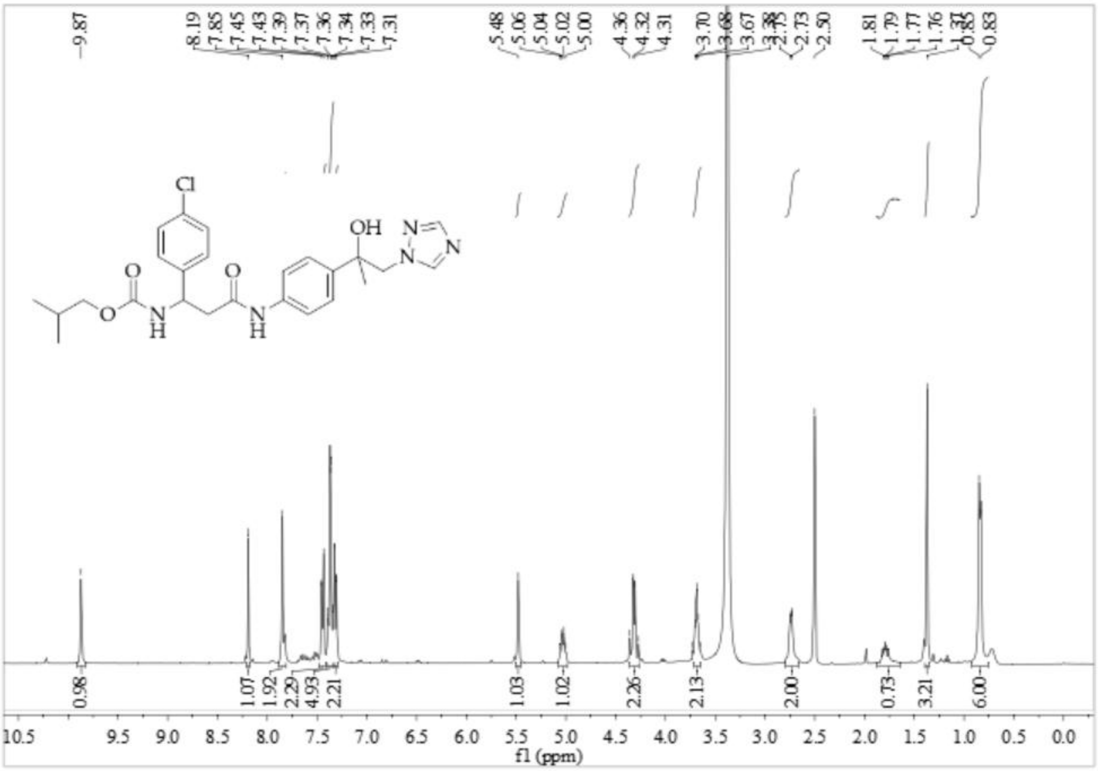

Figure S41. ^{1}H NMR spectrum of **9f**

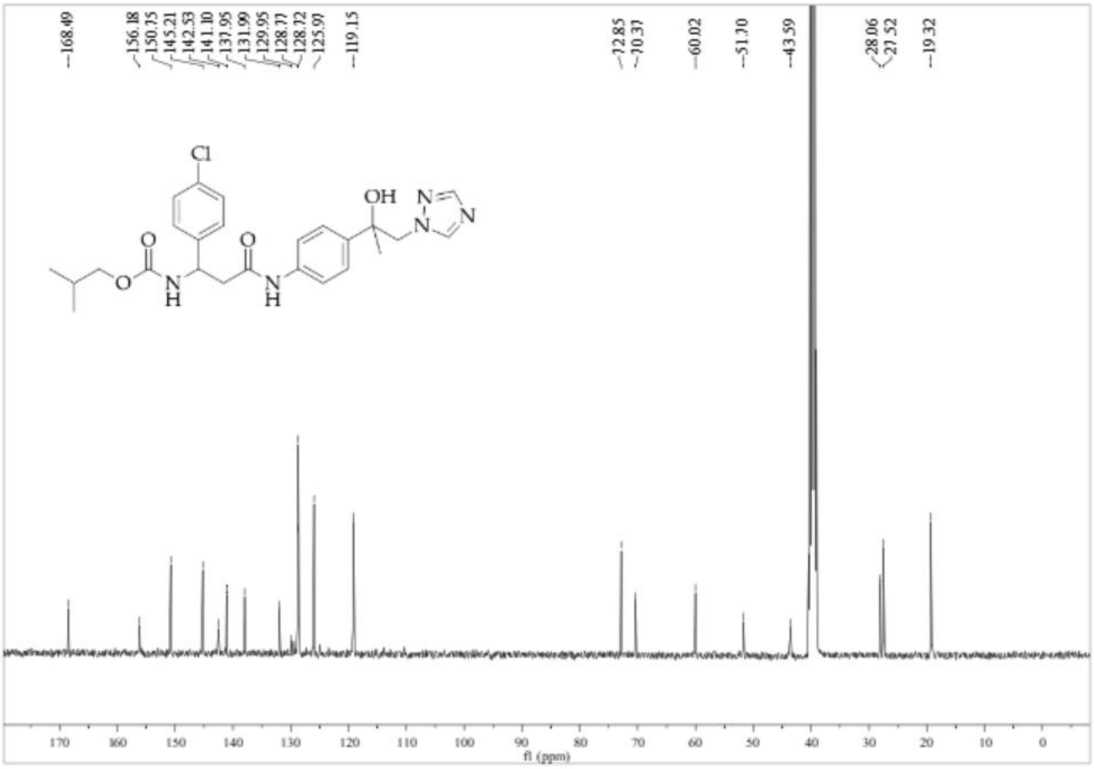

Figure S42. ^{13}C NMR spectrum of **9f**

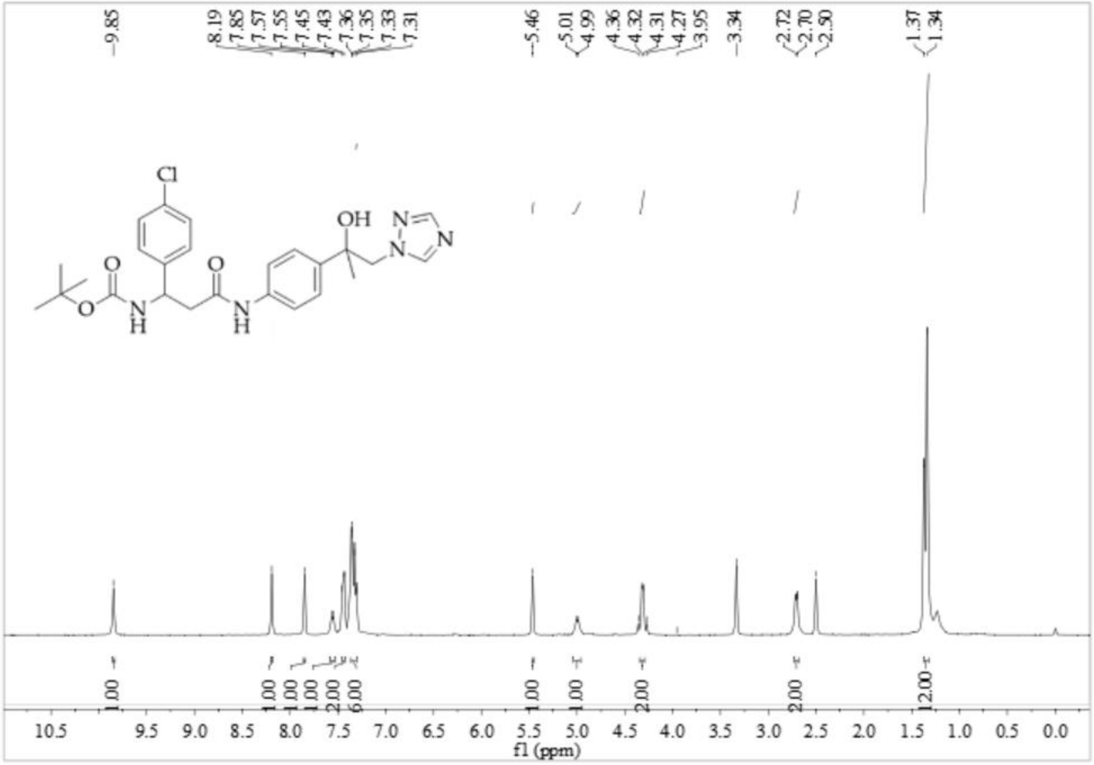

Figure S43. ^{1}H NMR spectrum of **9g**

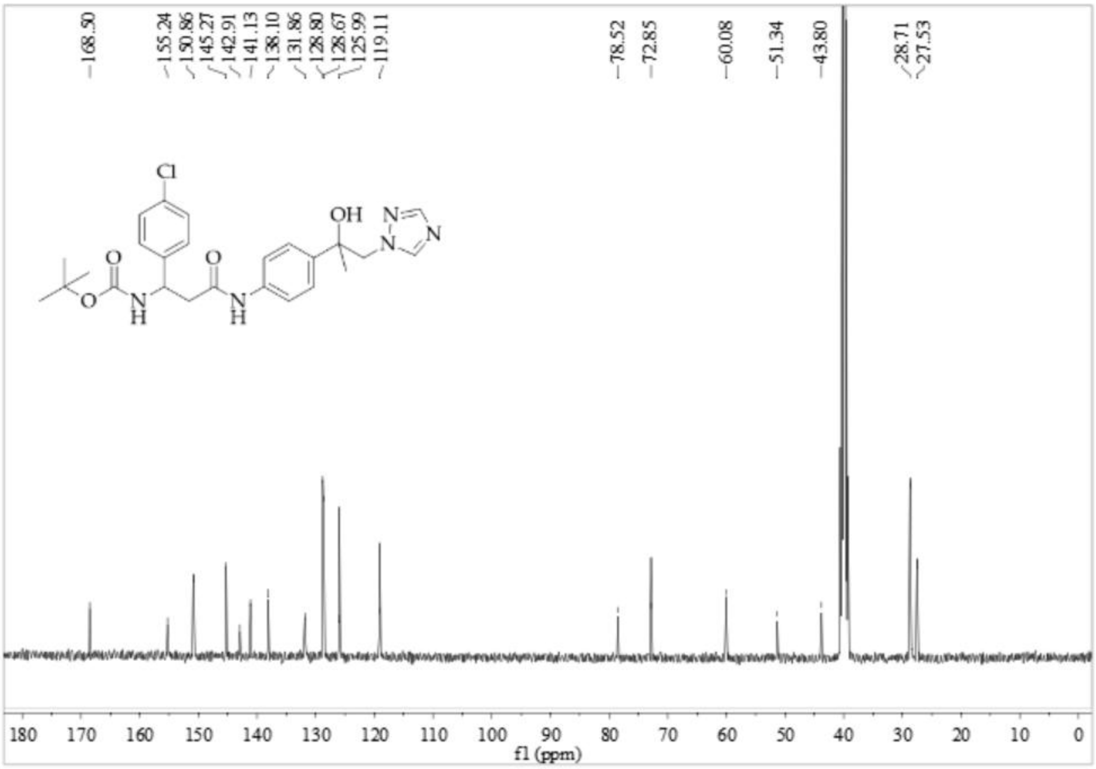

Figure S44. ^{13}C NMR spectrum of **9g**

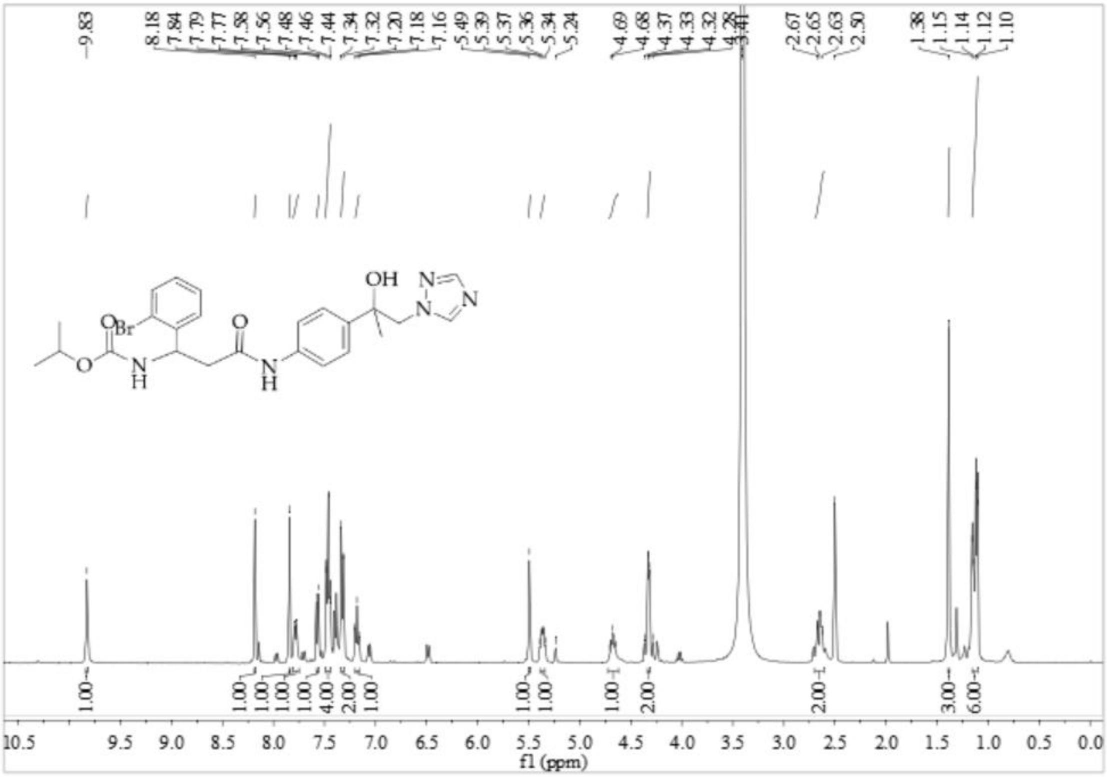

Figure S45. ^{1}H NMR spectrum of **9h**

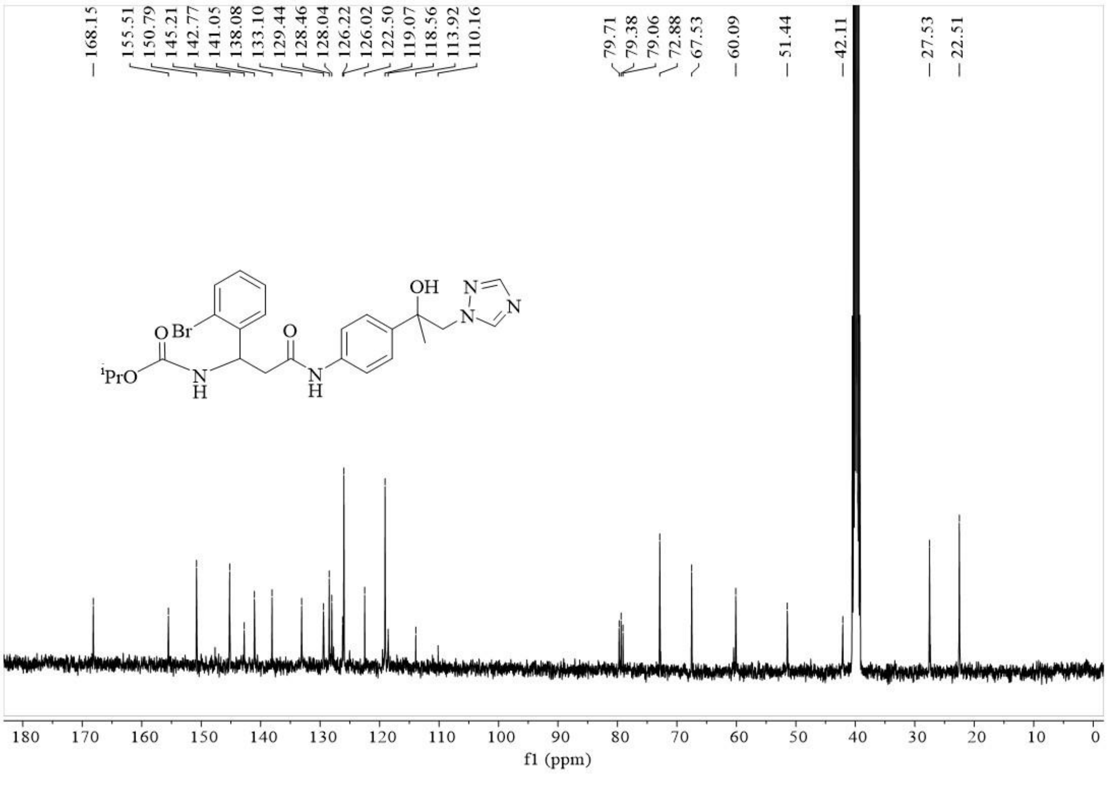

Figure S46. ^{13}C NMR spectrum of **9h**

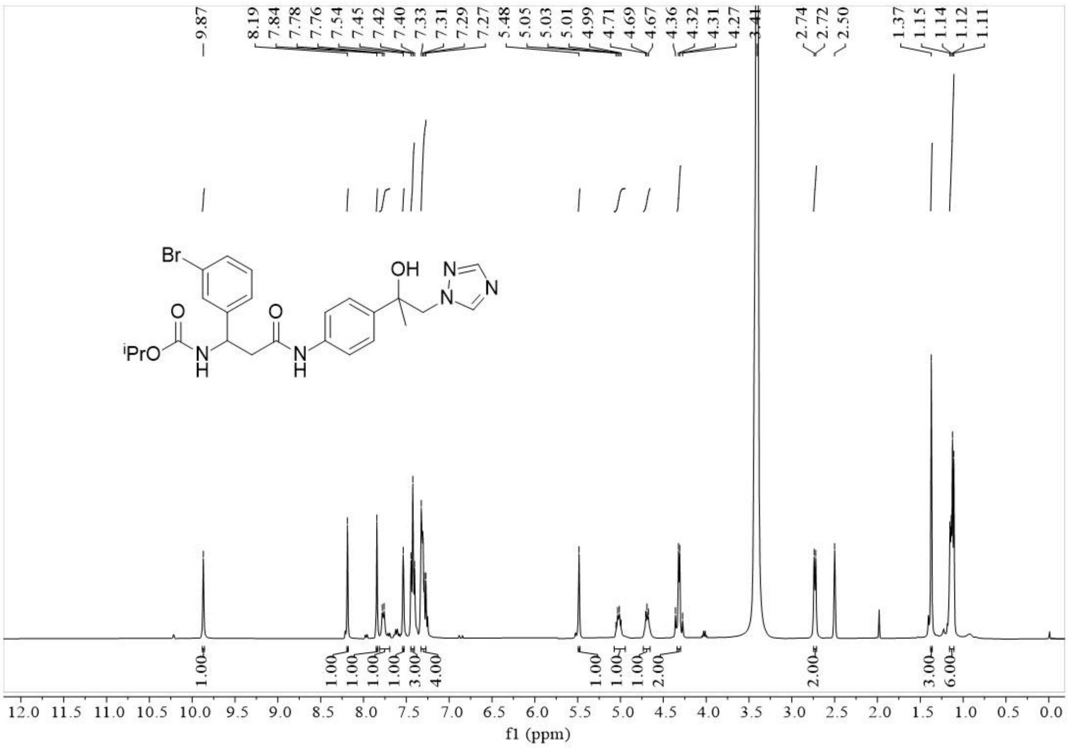

Figure S47. ^{1}H NMR spectrum of **9i**

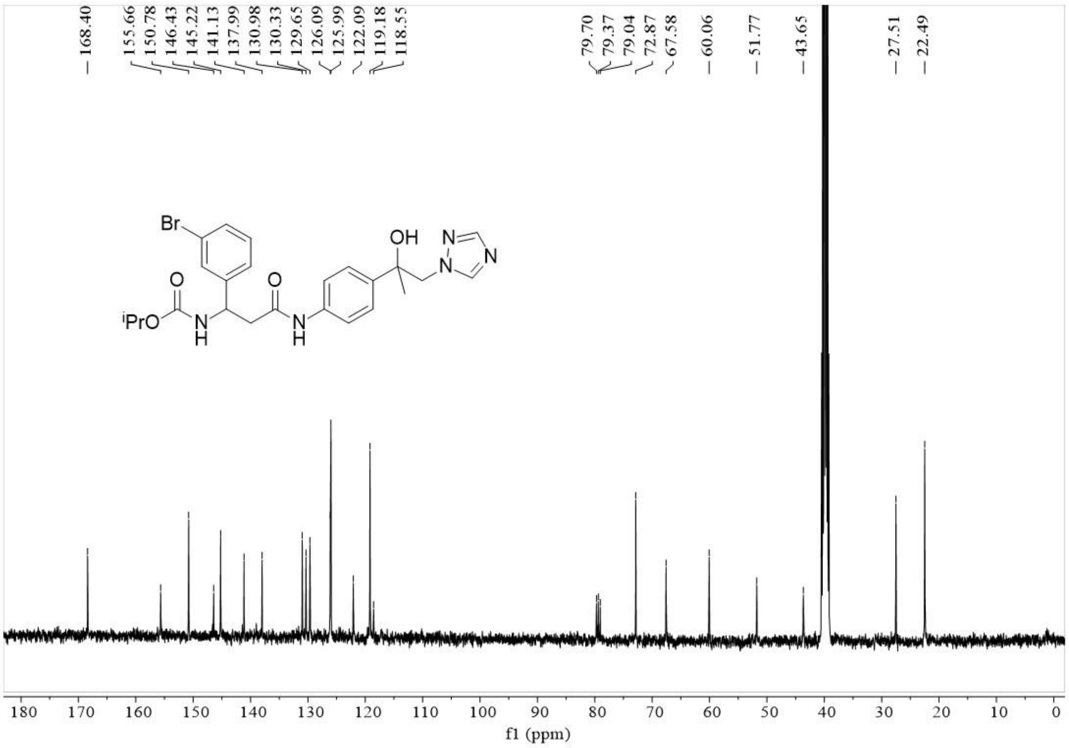

Figure S48. ^{13}C NMR spectrum of **9i**

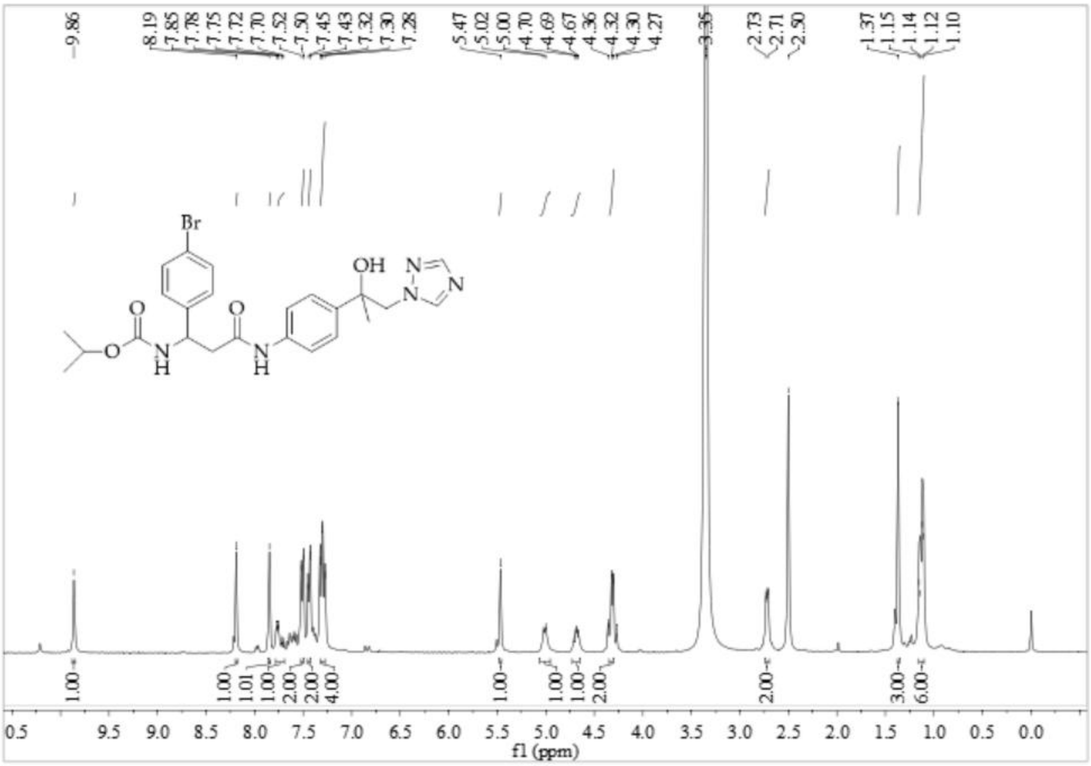

Figure S49. ^{1}H NMR spectrum of **9j**

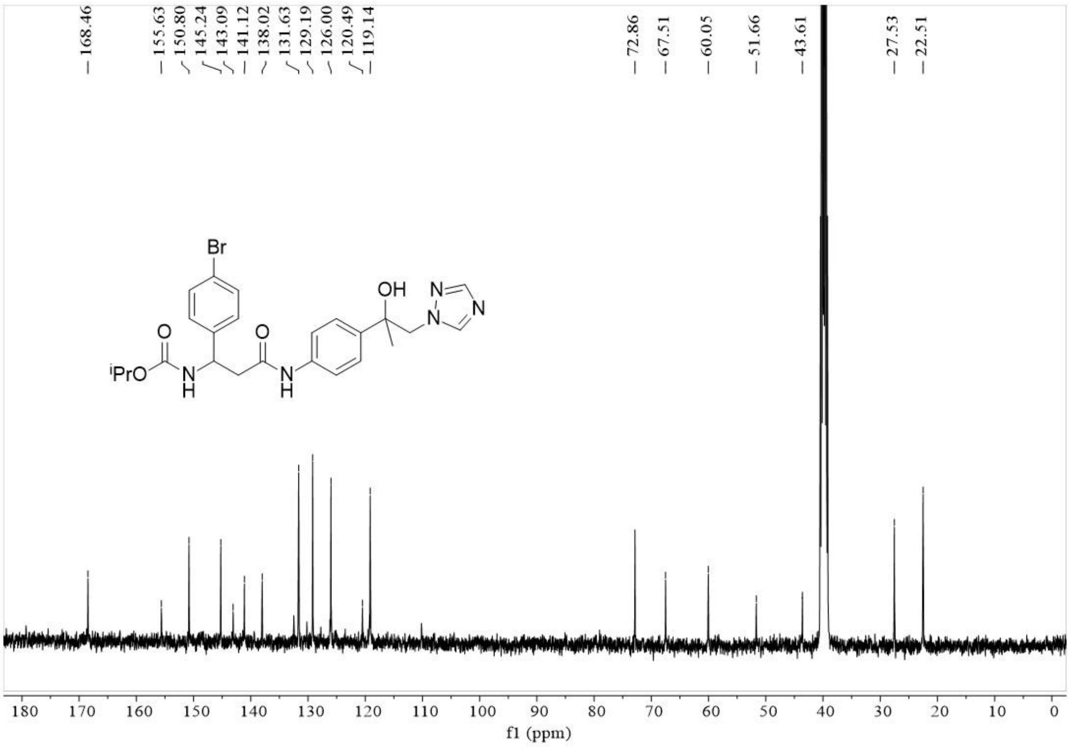

Figure S50. ^{13}C NMR spectrum of **9j**

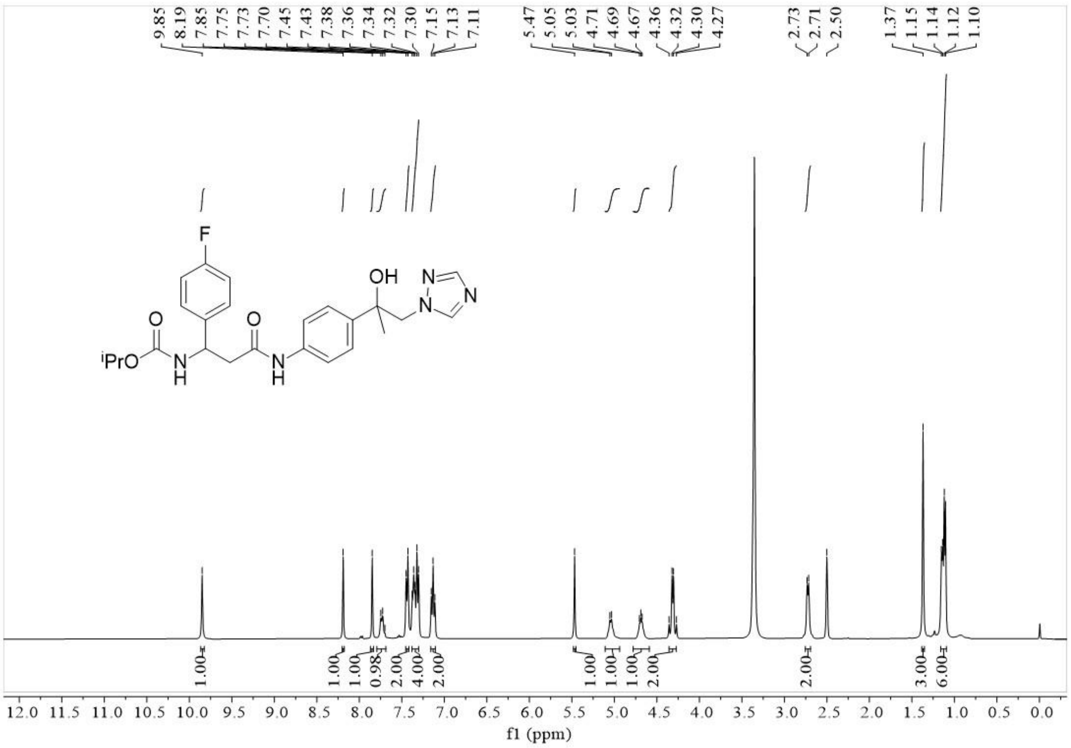

Figure S51. ^{1}H NMR spectrum of **9k**

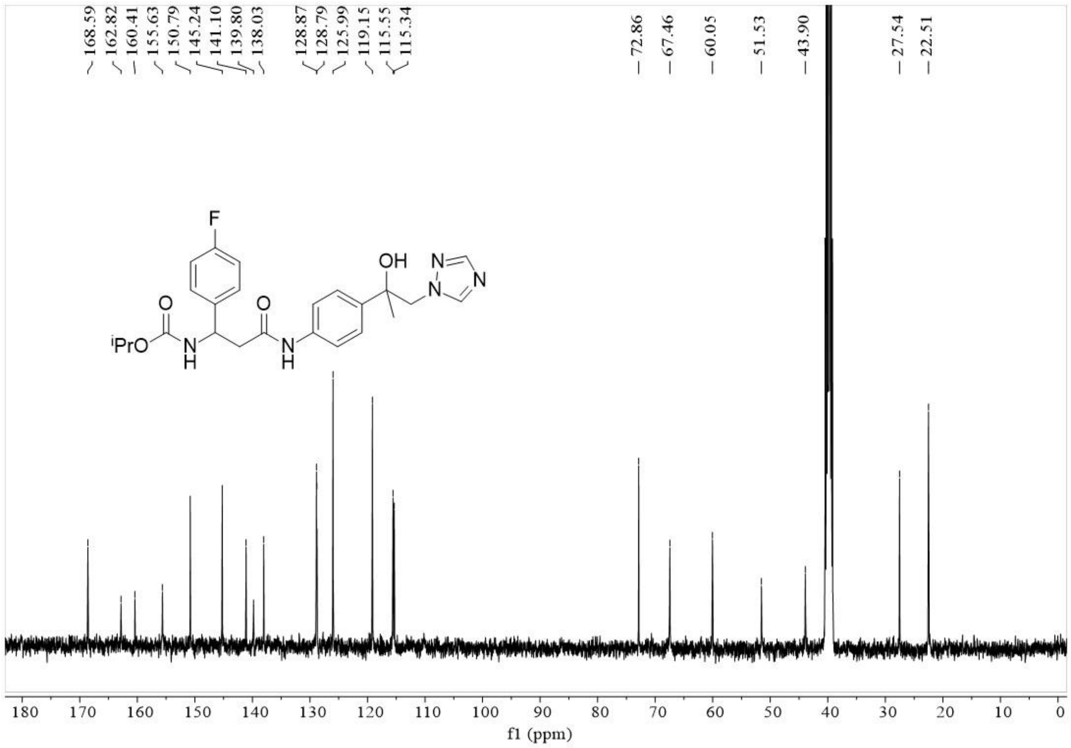

Figure S52. ^{13}C NMR spectrum of **9k**

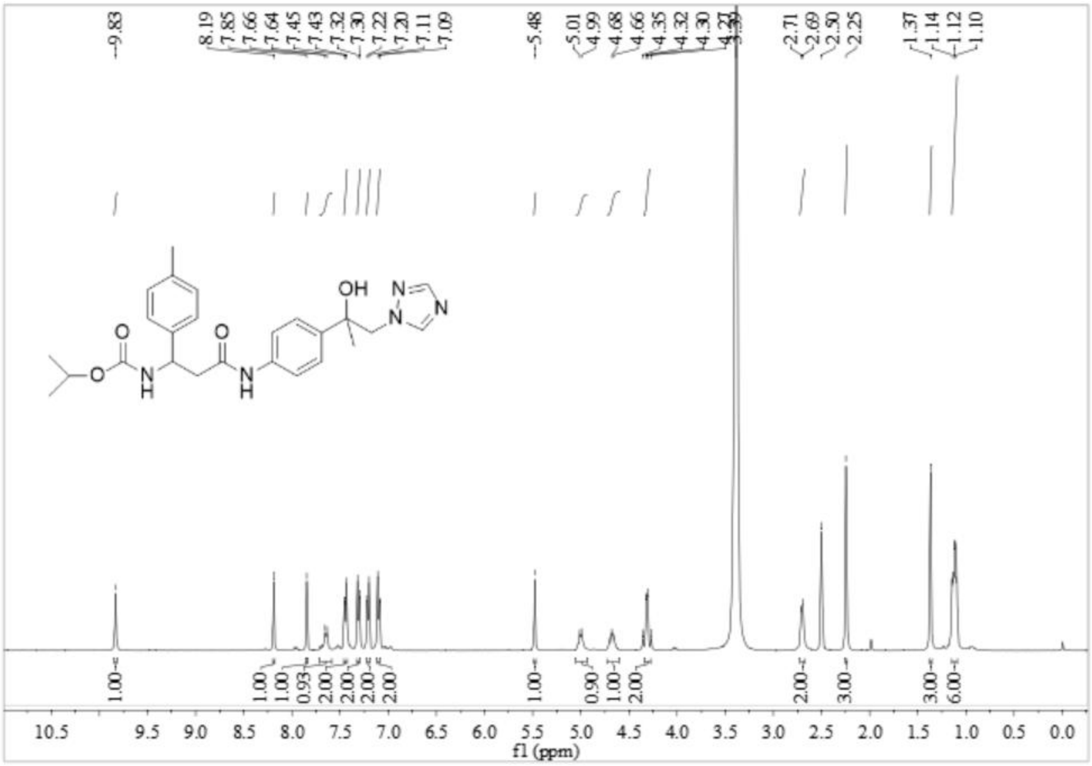

Figure S53. ^{1}H NMR spectrum of **9l**

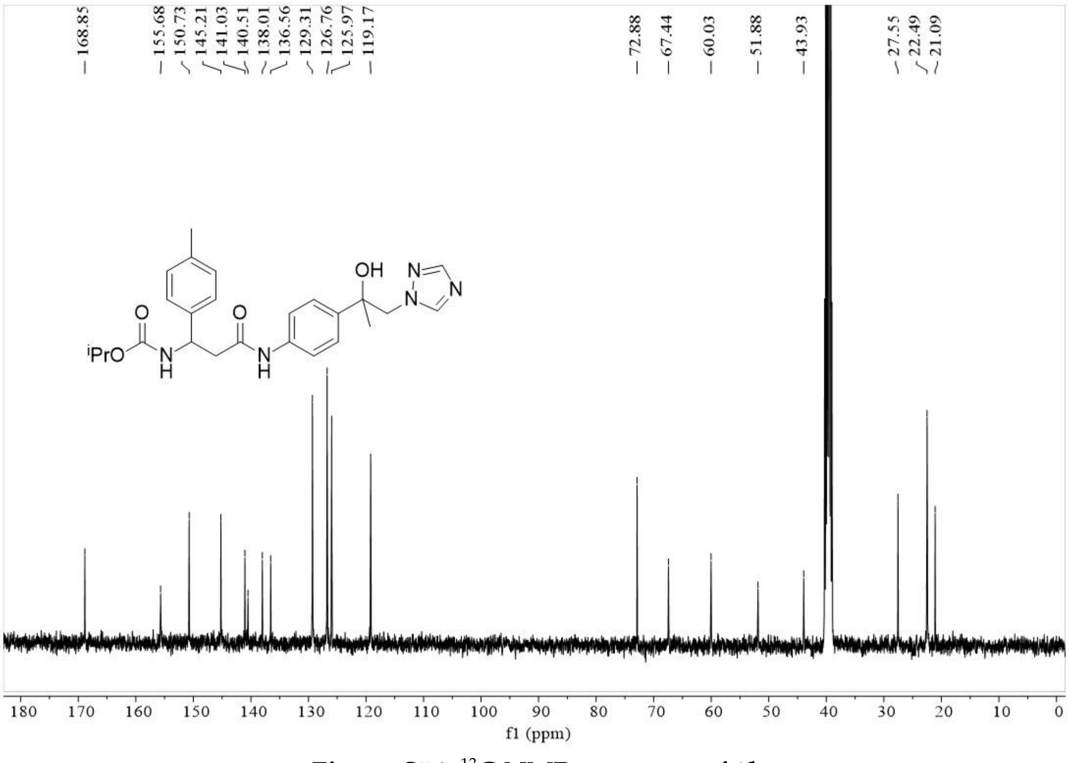

Figure S54. ^{13}C NMR spectrum of **9l**

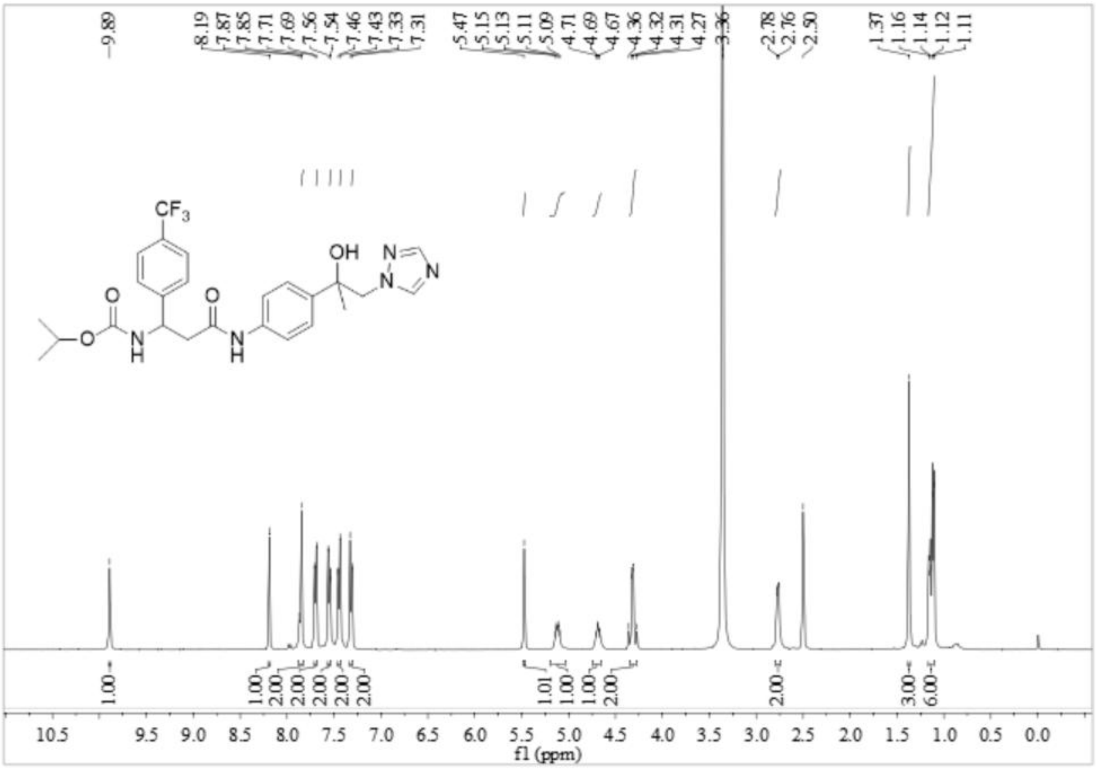

Figure S55. ^{1}H NMR spectrum of **9m**

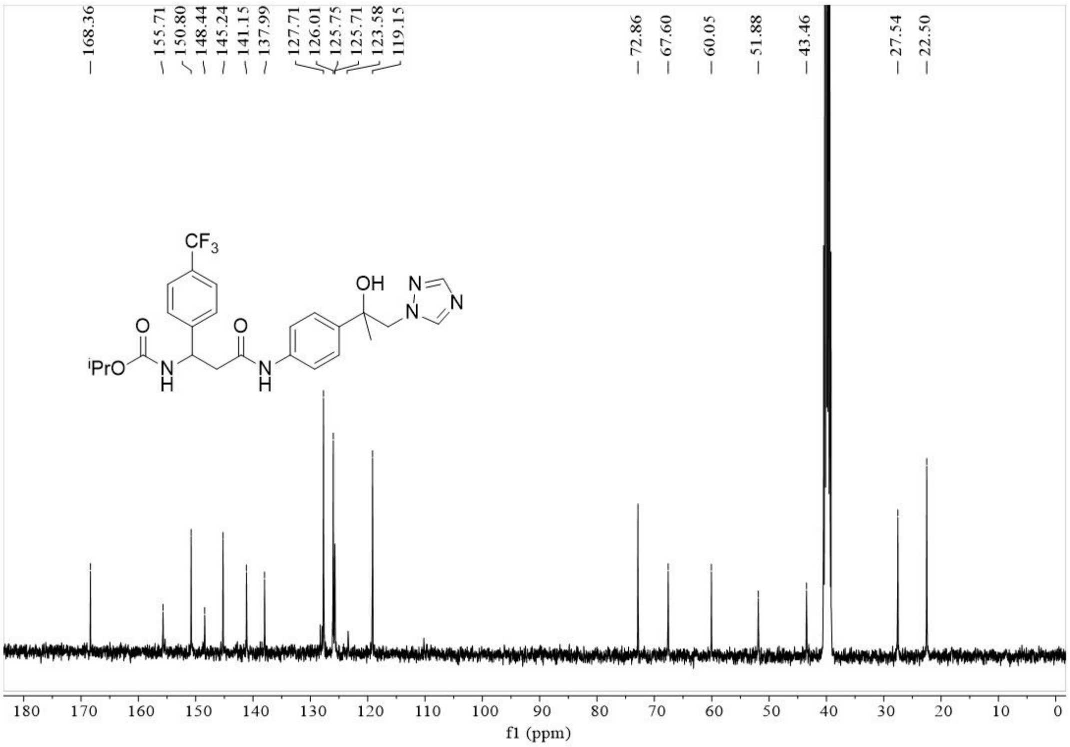

Figure S56. ^{13}C NMR spectrum of **9m**

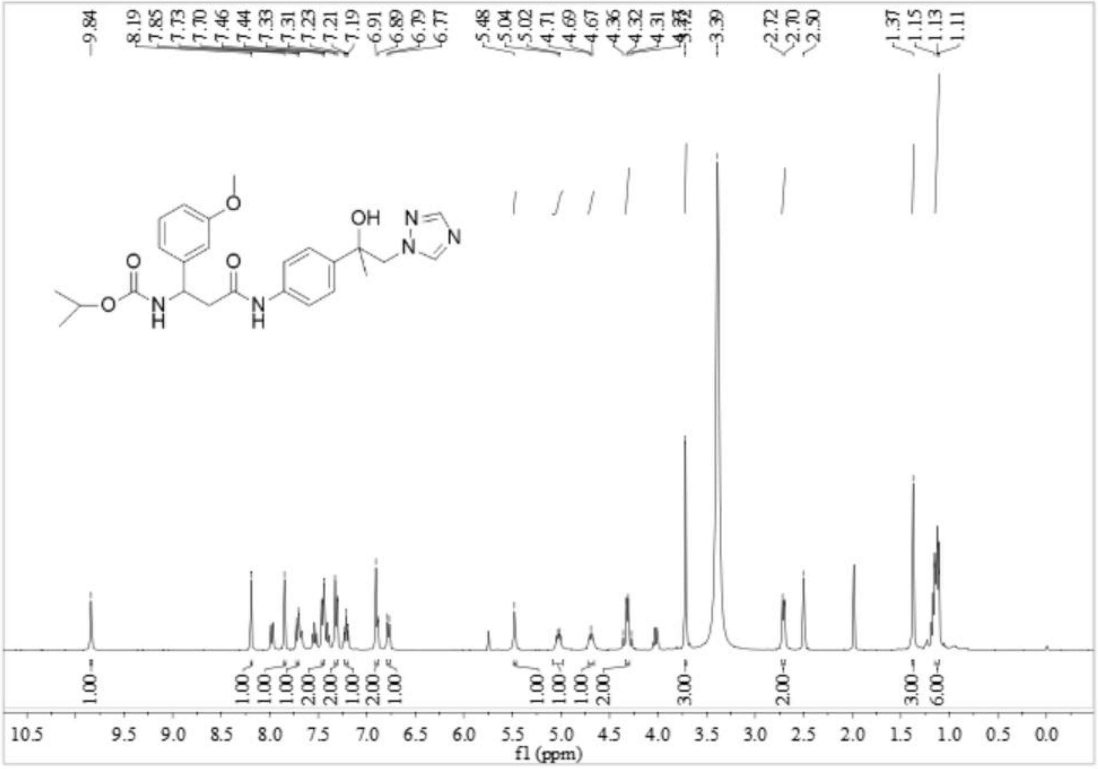

Figure S57. ^{1}H NMR spectrum of **9n**

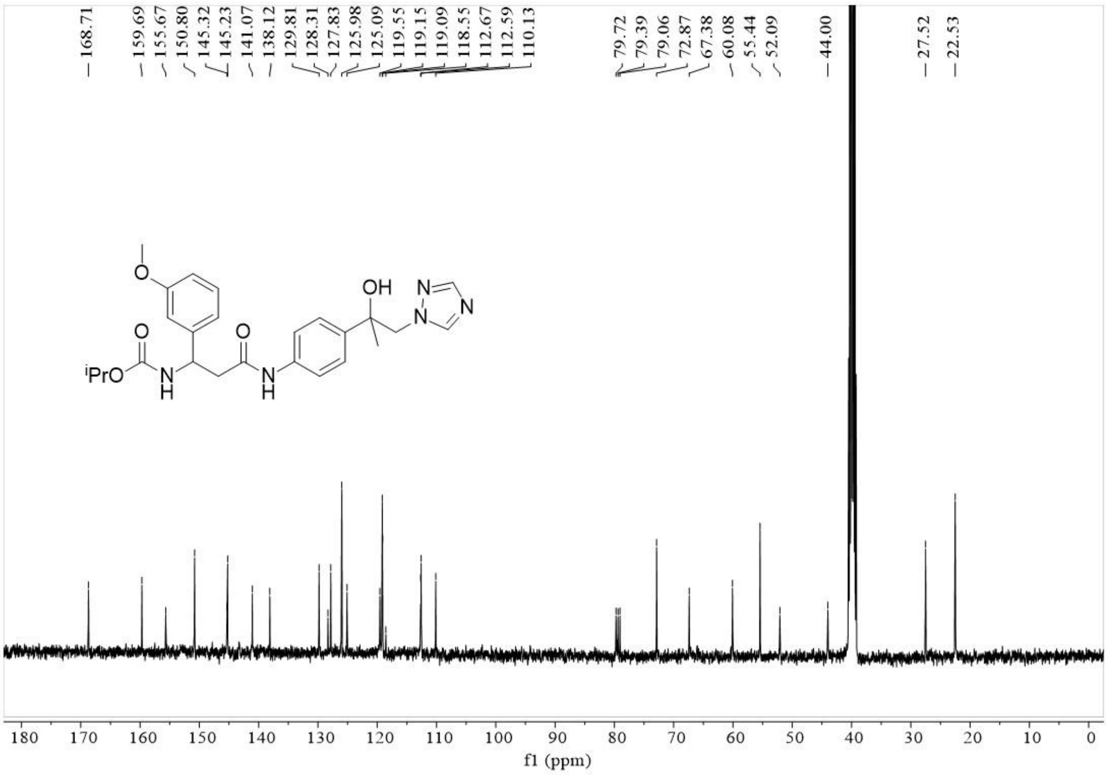

Figure S58. ^{13}C NMR spectrum of **9n**

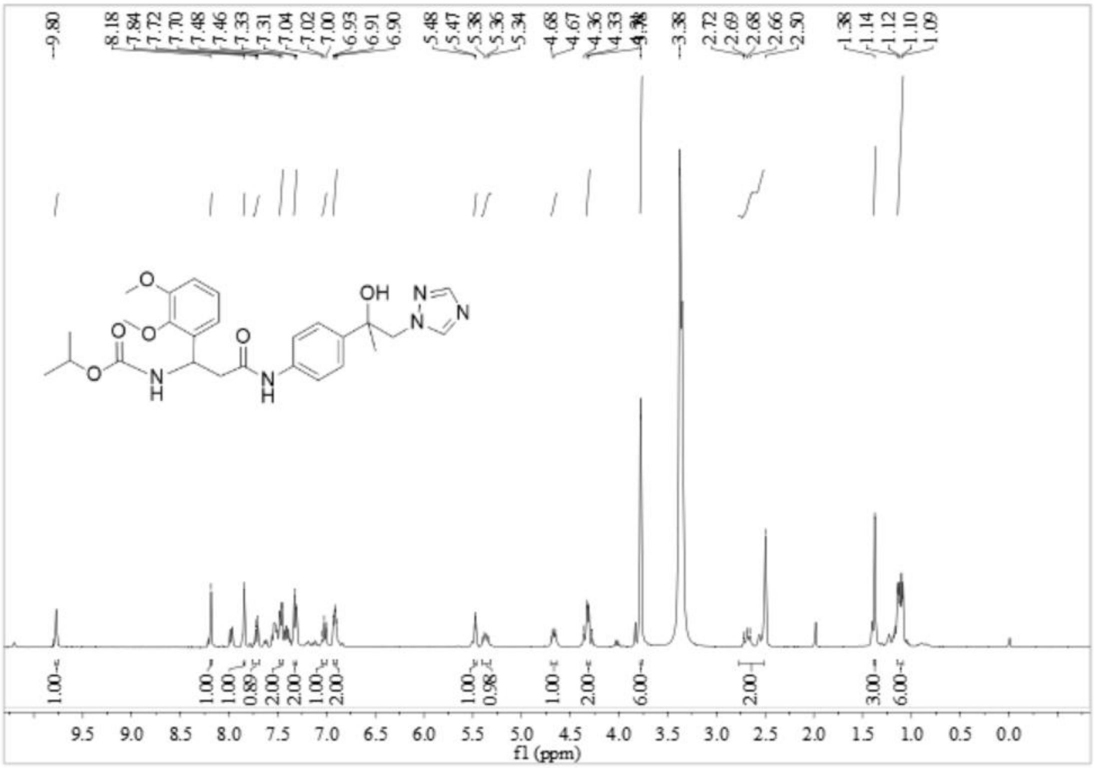

Figure S59. ^{1}H NMR spectrum of **9o**

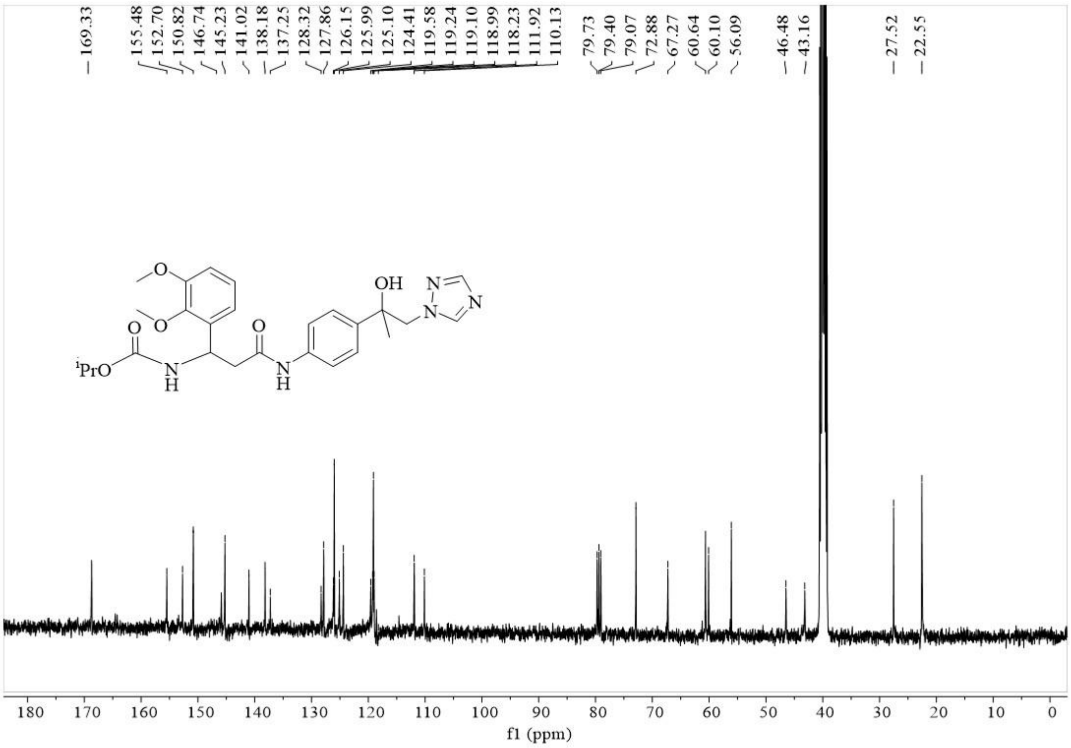

Figure S60. ^{13}C NMR spectrum of **9o**

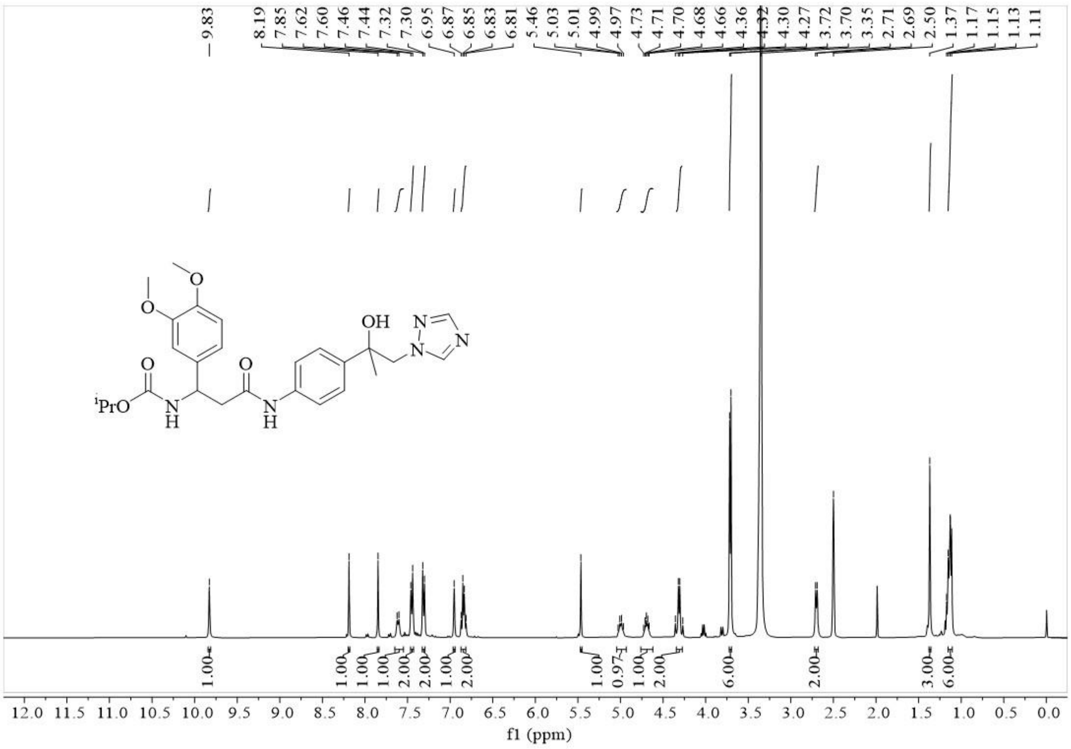

Figure S61. ^{1}H NMR spectrum of **9p**

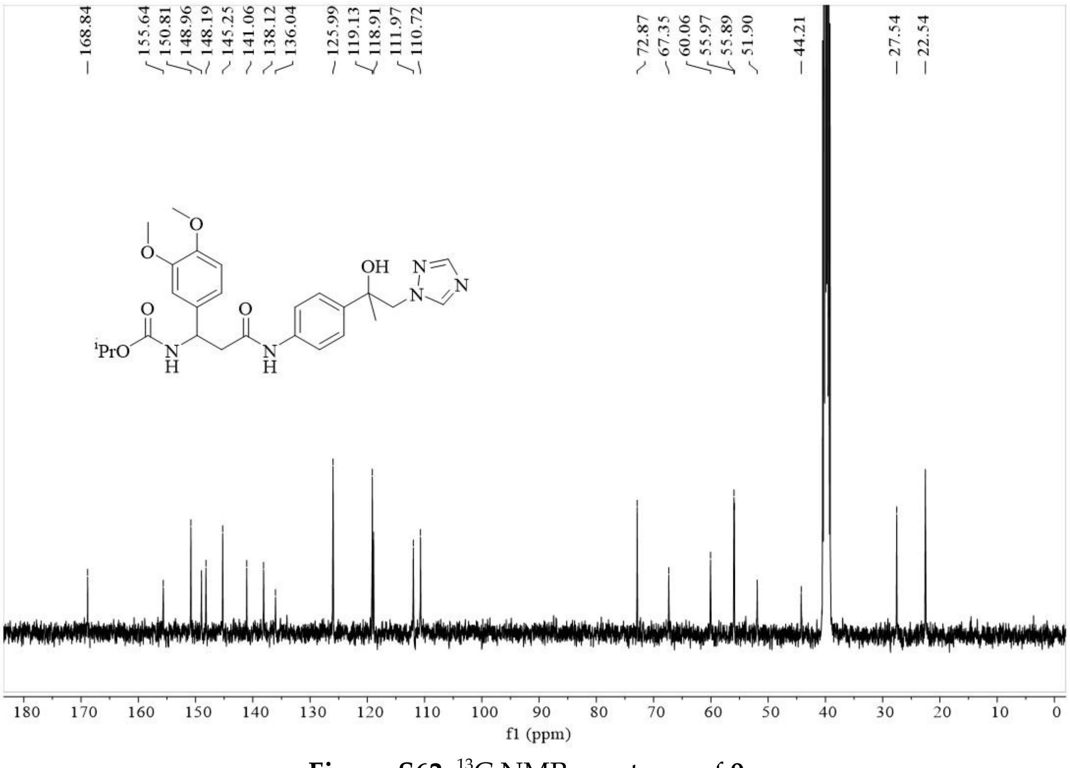

Figure S62. ^{13}C NMR spectrum of **9p**

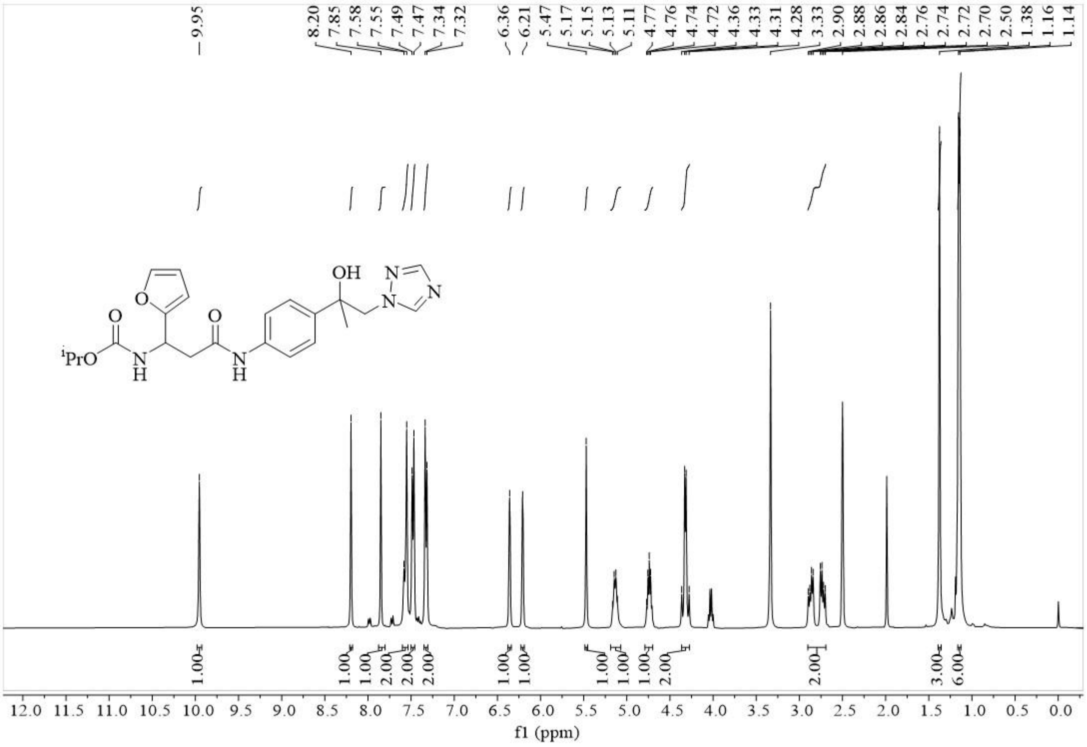

Figure S63. ^{1}H NMR spectrum of **9q**

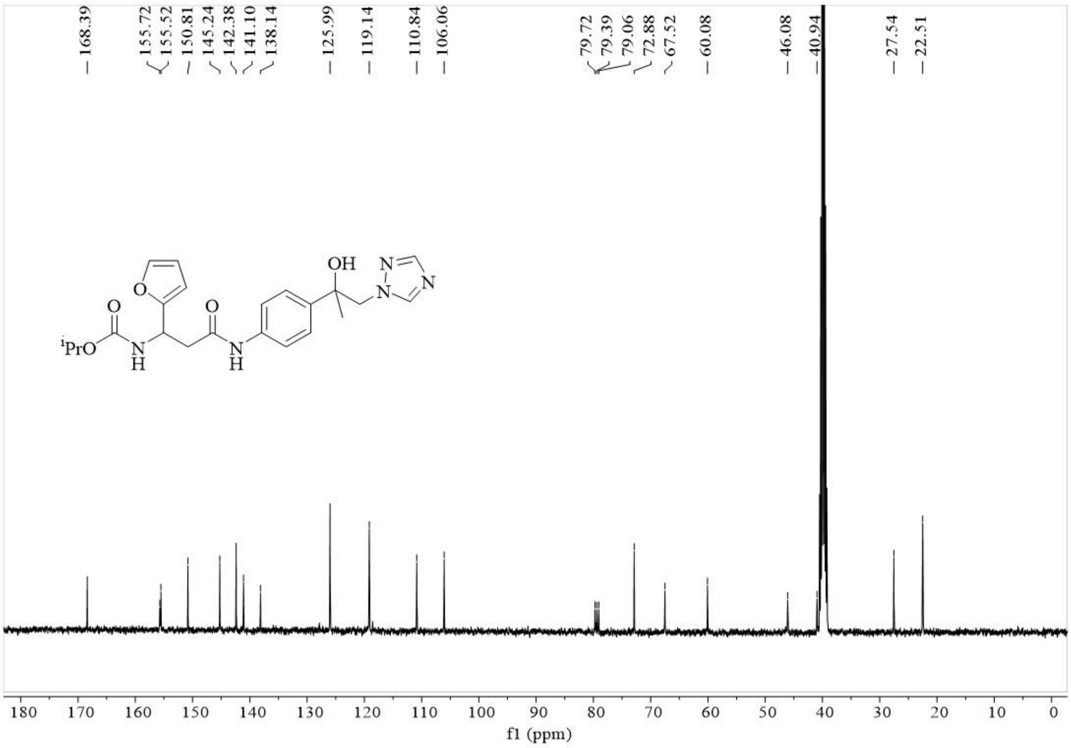

Figure S64. ^{13}C NMR spectrum of **9q**

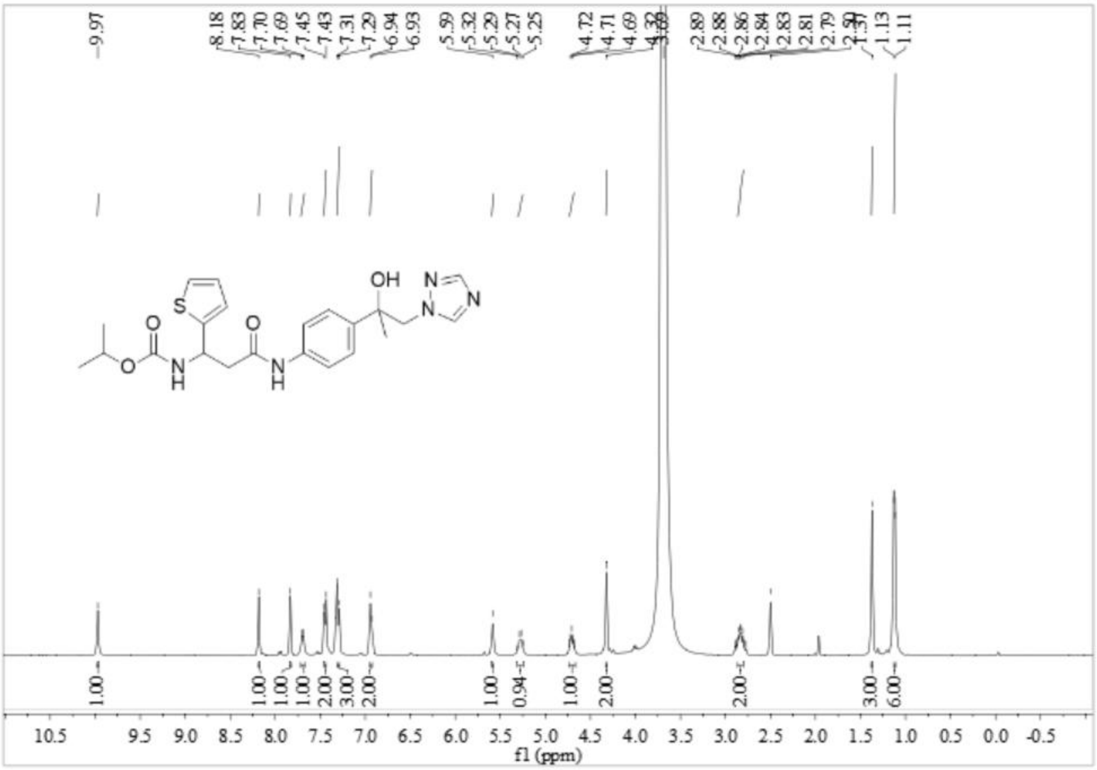

Figure S65. ^{1}H NMR spectrum of **9r**

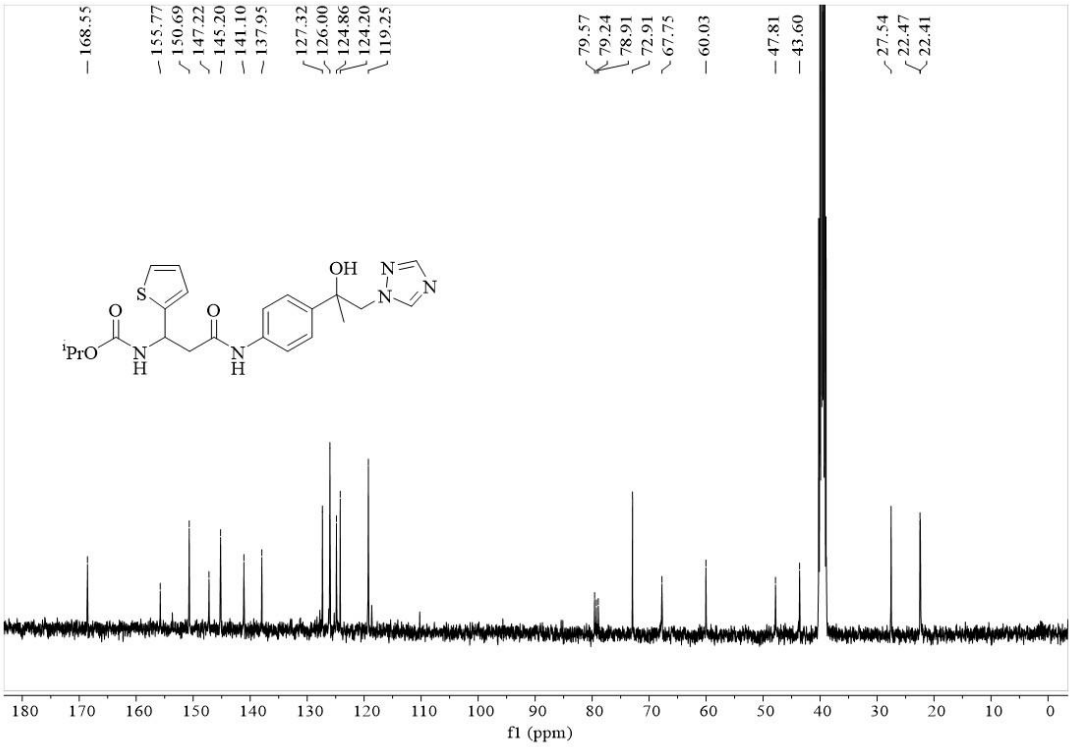

Figure S66. ^{13}C NMR spectrum of **9r**
